# Development of a flow photochemical process for a π-Lewis acidic metal-catalyzed cyclization/radical addition sequence: in situ-generated 2-benzopyrylium as photoredox catalyst and reactive intermediate

**DOI:** 10.3762/bjoc.20.173

**Published:** 2024-08-13

**Authors:** Masahiro Terada, Zen Iwasaki, Ryohei Yazaki, Shigenobu Umemiya, Jun Kikuchi

**Affiliations:** 1 Department of Chemistry, Graduate School of Science, Tohoku University, Aoba-ku, Sendai 980-8578, Japan; 2 Graduate School of Pharmaceutical Science, Tohoku University, Aoba-ku, Sendai 980-8578, Japan

**Keywords:** 2-benzopyrylium, flow chemistry, isocromene, photochemical reaction, π-Lewis acidic metal

## Abstract

A flow photochemical reaction system for a π-Lewis acidic metal-catalyzed cyclization/radical addition sequence was developed, which utilizes in situ-generated 2-benzopyrylium intermediates as the photoredox catalyst and electrophilic substrates. The key 2-benzopyrylium intermediates were generated in the flow reaction system through the intramolecular cyclization of *ortho*-carbonyl alkynylbenzene derivatives by the π-Lewis acidic metal catalyst AgNTf_2_ and the subsequent proto-demetalation with trifluoroacetic acid. The 2-benzopyrylium intermediates underwent further photoreactions with benzyltrimethylsilane derivatives as the donor molecule in the flow photoreactor to provide 1*H*-isochromene derivatives in higher yields in most cases than the batch reaction system.

## Introduction

Flow chemistry has been actively studied in recent years as a method to run a reaction continuously using a flow path or tube, rather than in a flask [1–16]. This method has attracted much attention because, unlike a batch reaction system, it allows for rapid generation of unstable chemical species by controlling parameters such as flow velocity and mixing properties, and in some cases makes it possible to achieve reactions that are difficult to perform using batch chemistry [17–21]. In general, efficient two-phase mixing and heat transfer, as well as ease of scale-up, are the advantages of using a flow system. In addition, reproducibility in a liquid–liquid flow system is improved because the flow velocity and temperature can be precisely controlled by using a syringe pump and a temperature control unit, respectively. Moreover, as the reaction mixture continues to flow and the reaction can be quenched immediately when necessary, the decomposition of an unstable product under the reaction conditions can be avoided [22–25]. Furthermore, when a photoreaction is performed in a flow system, there is an advantage that the light irradiation efficiency [26–29] is increased. Thus, the flow photochemical process is crucial and beneficial to product formation.

**Scheme 1:**
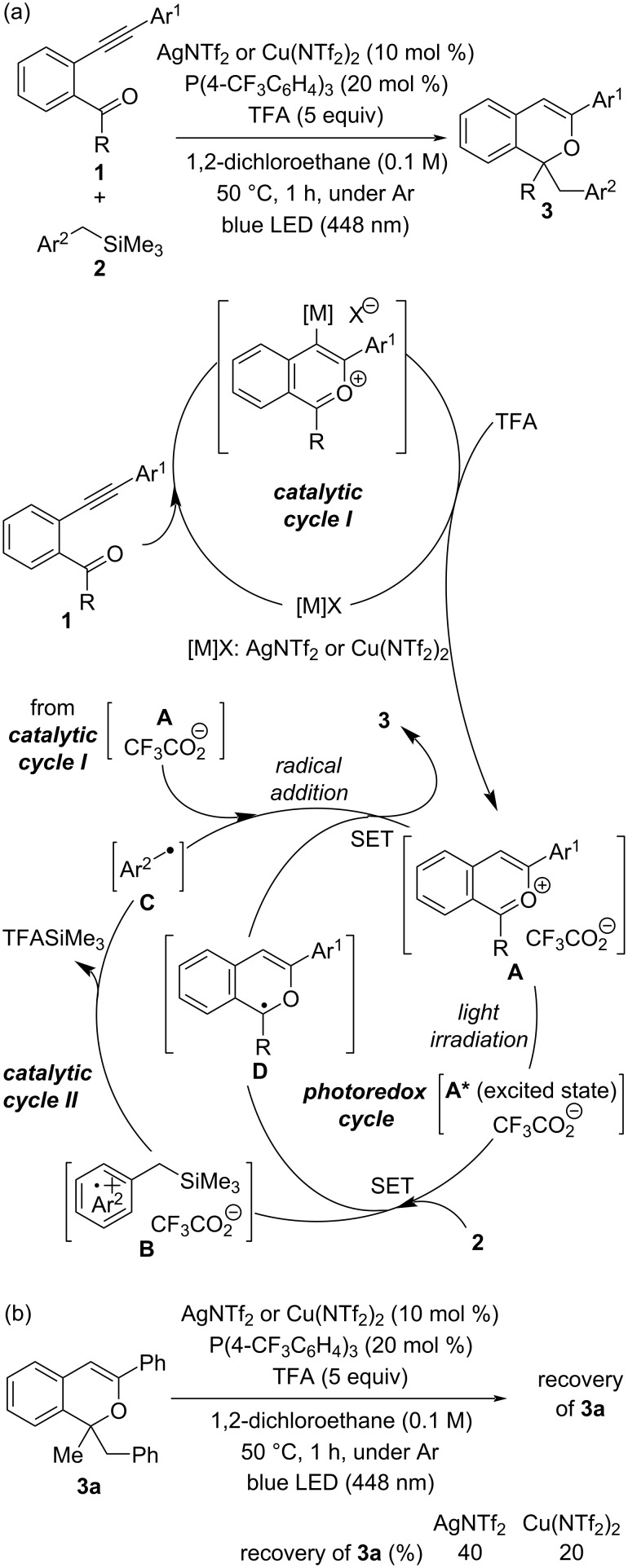
(a) Sequential π-Lewis acidic metal-catalyzed cyclization/photochemical radical addition for the formation of 1*H*-isochromene derivatives **3** and its plausible catalytic cycles. (b) Stability of **3a** under the optimal reaction conditions of the batch reaction.

Recently, we reported a sequential transformation consisting of a π-Lewis acidic metal-catalyzed cyclization [30–45] and subsequent photochemical radical addition [46–54], which affords 1*H*-isochromene derivatives **3** through three catalytic cycles (Scheme 1a) [55]: catalytic cycles I and II and a photoredox cycle of the photocatalyst [56–57] (see Supporting Information File 1 for the overall catalytic cycles). In catalytic cycle I, the key cationic components, 2-benzopyrylium intermediates **A**, are generated in situ by the activation of the alkyne moiety of *ortho*-carbonyl alkynylbenzene derivatives **1** in the presence of the π-Lewis acidic metal catalyst [M]X [AgNTf_2_ or Cu(NTf_2_)_2_] and subsequent intramolecular cyclization followed by proto-demetalation with trifluoroacetic acid (TFA). In catalytic cycle II, photoexcitation of the generated 2-benzopyrylium intermediates **A** under light irradiation facilitates single-electron transfer (SET) from benzyltrimethylsilane derivatives **2** as the donor molecule, initiating further radical reactions through the formation of radical cations **B**. Nucleophilic arylmethyl radicals **C**, which are generated from radical cations **B** by desilylation, undergo an addition reaction with 2-benzopyrylium intermediates **A**, giving rise to the corresponding radical cation. Catalytic cycle II is completed through a SET from **D**, a reduced form of the photoredox catalyst 2-benzopyrylium intermediates **A**, to the generated radical cation, affording 1*H*-isochromene derivatives **3**. The photoredox cycle is also completed with the regeneration of cations **A** through SET from **D**.

The most distinctive feature of this sequential transformation is that the in situ-generated 2-benzopyrylium intermediates **A** are used not only as an electrophile but also as a photoredox catalyst. However, as this reaction is carried out under relatively harsh conditions (i.e., light irradiation, use of an excess amount of TFA), the stability of products **3** was a concern. Indeed, subjecting product **3a** to the optimal reaction conditions with either AgNTf_2_ or Cu(NTf_2_)_2_ resulted in the significant degradation of **3a**, although the degradation of **3a** was partially suppressed when AgNTf_2_ was used (Scheme 1b). Accordingly, we envisioned that the characteristics of the flow photochemical process, i.e., efficient light irradiation and immediate separation of the formed product from the reaction system, would be suitable for this sequential reaction. Here, we report the results of our investigation on the use of a flow photochemical reaction system to improve the yield of the present sequential transformation.

## Results and Discussion

At the outset of our studies to optimize the flow reaction conditions, we employed AgNTf_2_ as the π-Lewis acidic metal catalyst because of its high solubility in 1,2-dichloroethane (1,2-DCE) [58] and ability to partially suppress the degradation of the product formed (Scheme 1b). When designing a flow reaction system for the present sequential transformation, we considered the fact that the transformation involves three catalytic cycles. In particular, given that catalytic cycle I (see Scheme 1a) generates, e.g., key cationic components, 2-benzopyrylium intermediates **A** without light irradiation, it is necessary to ensure that the reaction time of catalytic cycle I is not affected by the timescale of the flow reaction. Therefore, we adopted a dual syringe system in which two solutions are mixed before being introduced into the photoreactor (volume: 1.0 mL) (Table 1, top right). After several trials, we decided to fill syringe A with *o-*alkynylacetophenone **1a** and syringe B with AgNTf_2_, P(4-CF_3_C_6_H_4_)_3_, benzyltrimethylsilane (**2a**, TMSBn), and TFA (see Supporting Information File 1 for details). At this time, the volumes of the solutions placed in the two syringes were adjusted to be approximately the same. The initial conditions of the flow reaction were based on those of the batch reaction [0.1 mmol of **1a**, 10 μmol (10 mol %) of AgNTf_2_, 20 μmol (20 mol %) of P(4-CF_3_C_6_H_4_)_3_, 1.0 mmol (10 equiv) of **2a**, and 0.5 mmol (5 equiv) of TFA under light irradiation (blue LED: λ_max_ = 448 nm) at 50 °C for 1 h in 1 mL (total volume) of 1,2-DCE] [55] with a flow rate of 3 mL/h (light irradiation time: 20 min in the flow reaction, 1 h in the batch reaction). As shown in Table 1, product **3a** was obtained in moderate yield (entry 1: 42%, cf. batch reaction: 76%). Lowering the reaction temperature to 25 °C reduced the yield (Table 1, entry 2: 35%), but decreasing the amount of the phosphine ligand from 20 mol % to 5 mol % markedly improved the yield (Table 1, entry 3: 53%). Even when the flow rate was increased from 3 mL/h to 24 mL/h (light irradiation time was shortened from 20 min to 2.5 min), the yield of **3a** was maintained (Table 1, entry 4: 53%). Under these conditions, no improvement in yield was observed when the premixing zone (0.7 mL) was provided (Table 1, entry 5: 52%); however, the effect of adding the premixing zone was remarkable when the amount of AgNTf_2_ was reduced by half (5 mol %; Table 1, entry 6 vs entry 7: 26% vs 49%). These results suggest that the generation of 2-benzopyrylium intermediates **A**, (i.e., catalytic cycle I) requires a certain reaction time (at this flow rate: ca. 2 min). Moreover, the yield of **3a** was improved when the concentration of **1a** was lowered from 0.1 M to 0.05 M (Table 1, entry 8: 61%). Meanwhile, further reducing the catalyst loading from 5 mol % to 2 mol % resulted in a significant decrease in yield (Table 1, entry 9: 28%). When the reaction was scaled up from 0.1 mmol to 0.5 mmol of **1a** in consideration of the dead volume of the flow reactor, the product **3a** was obtained in markedly improved yield (Table 1, entry 10: 77%) [59] which was comparable to that of the batch reaction (76%). Notably, however, the present flow reaction was performed at 25 °C (batch: 50 °C) with half the amount of AgNTf_2_ (flow: 5 mol %, batch: 10 mol %), and the light irradiation time was shortened to only 2.5 minutes (batch: 1 h). Thus, under the optimal conditions (Table 1, entry 10), the flow reaction system proved extremely useful for improving the efficiency of the present photochemical sequential transformation.

**Table 1:** Screening of reaction conditions in the flow reaction system^a^.

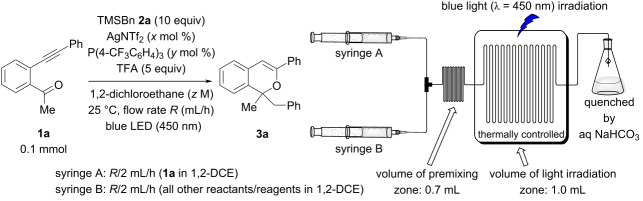							

Entry	AgNTf_2_ (*x* mol %)	P(4-CF_3_C_6_H_4_)_3_ (*y* mol %)	Conc. of **1a** (*z* M)	Flow rate (*R* mL/h)	Premixing zone (mL)	Yield of **3a** (%)^b^	Recovery of **1a** (%)^b^

1^c^	10	20	0.1	3	none	42	0
2	10	20	0.1	3	none	35	22
3	10	5	0.1	3	none	53	1
4	10	5	0.1	24	none	53	0
5	10	5	0.1	24	0.7	52	9
6	5	2.5	0.1	24	none	26	14
7	5	2.5	0.1	24	0.7	49	0
8	5	2.5	0.05	24	0.7	61	0
9	2	1	0.05	24	0.7	28	28
10^d^	5	2.5	0.05	24	0.7	77	0

^a^Unless otherwise noted, all reactions were carried out in a flow photochemical reactor (volume: 1.0 mL, λ_max_ = 450 nm) using a dual syringe system, as shown in the table scheme. Syringe A: 0.1 mmol of **1a** in 1,2-DCE (0.55 mL). Syringe B: AgNTf_2_, P(4-CF_3_C_6_H_4_)_3_, TMSBn **(2a**), and TFA in 1,2-DCE (0.45 mL); ^b^Yield was determined by NMR analysis using 1,1,2,2-tetrabromoethane as an internal standard; ^c^At 50 °C. ^d^Reaction was conducted on a 0.5 mmol scale. Syringe A: 0.5 mmol of **1a** in 1,2-DCE (5.4 mL). Syringe B: 25 μmol (5 mol %) of AgNTf_2_, 12.5 μmol (2.5 mol %) of P(4-CF_3_C_6_H_4_)_3_, 5 mmol (10 equiv) of TMSBn **(2a**), and 2.5 mmol (5 equiv) of TFA in 1,2-DCE (4.6 mL).

With the optimal flow reaction conditions in hand, we next investigated the scope of substrates **1** by introducing a series of substituents to the terminal phenyl group. The results of the batch reaction system are also shown for comparison in Table 2 (right-hand side) [55]. As expected, the use of the flow reaction system significantly increased yields, although the yields obtained in the reactions of substrates having an electron-donating methoxy group were low to moderate regardless of the substitution pattern (Table 2, entries 1, 2, and 6). Indeed, when a methoxy group was introduced at the *para*-position, product **3b** was obtained in low yield (Table 2, entry 1: 14%). Because this yield was lower than that obtained in the batch reaction (48%), the flow reaction conditions for **1b** were thoroughly reconsidered (see Supporting Information File 1 for details). As a result, extending the premixing time and the light irradiation time (Table 2, entry 2) led to an improved yield; the obtained yield was higher than that of the batch reaction system even when half the amount of AgNTf_2_ was used with the temperature reduced to 25 °C (flow: 54% vs batch: 48%). Meanwhile, the reaction of substrate **1c** having a methyl group as a weak electron-donating group at the *para*-position afforded product **3c** in high yield (Table 2, entry 3: 76%). In addition, the reaction of **1d** having a bromo group resulted in a moderate yield, but with a significant improvement compared with the batch reaction (Table 2, entry 4: 57% vs 10%). The reaction of **1e** substituted with a strong electron-withdrawing trifluoromethyl group afforded the product **3e** in high yield (Table 2, entry 5: 77%), again confirming the high efficiency of the flow reaction system (batch: 54%). Next, the effects of the substituent at the *meta*-position were investigated. Substrate **1f** having a methoxy group afforded compound **3f** in only moderate yield (Table 2, entry 6: 39%), similar to the batch reaction (40%). The reactions of substrates having a methyl, bromo, or trifluoromethyl group gave the corresponding products **3g**–**i**, respectively, in good yields (Table 2, entries 7–9). The *ortho*-methyl-substituted substrate **1j** was also compatible, affording product **3j** in good yield (Table 2, entry 10: 76%). This yield was comparable to that of the substrate having a methyl group at the *para*- or *meta*-position, despite the steric hindrance of the *ortho*-substituent (Table 2, entry 10 vs entries 3 and 7). When a fluoro group was introduced to the tethering phenyl backbone, a high yield was obtained regardless of whether it was introduced at the 6- or 7-position (Table 2, entries 11 and 12).

**Table 2:** Scope of substrates^a^.

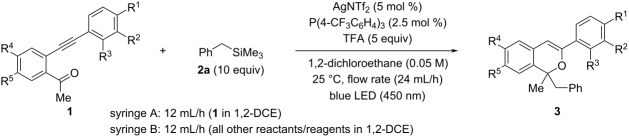									

Entry	**1**	R^1^	R^2^	R^3^	R^4^	R^5^	**3**	Yield of **3** (%)^b^	Batch reaction using AgNTf_2_. Yield of **3** (%)^b,c^

1	**1b**	MeO	H	H	H	H	**3b**	14	48^d^
2^e^	**1b**						**3b**	54	
3	**1c**	Me	H	H	H	H	**3c**	76	21
4	**1d**	Br	H	H	H	H	**3d**	57	10
5	**1e**	CF_3_	H	H	H	H	**3e**	77	65
6	**1f**	H	MeO	H	H	H	**3f**	39	40
7	**1g**	H	Me	H	H	H	**3g**	75	50
8	**1h**	H	Br	H	H	H	**3h**	68	63
9	**1i**	H	CF_3_	H	H	H	**3i**	65	42
10	**1j**	H	H	Me	H	H	**3j**	76	72
11	**1k**	H	H	H	F	H	**3k**	79	–
12	**1l**	H	H	H	H	F	**3l**	75	–

^a^Unless otherwise noted, all reactions were carried out in the flow photochemical reactor (volume: 1.0 mL, λ_max_ = 450 nm) having a 0.7 mL premixing zone using a dual syringe system with a flow rate of 24 mL/h (12 mL/h for each syringe) at 25 °C. Syringe A: 0.5 mmol of **1** in 1,2-DCE (5.4 mL). Syringe B: 25 μmol (5 mol %) of AgNTf_2_, 12.5 μmol (2.5 mol %) of P(4-CF_3_C_6_H_4_)_3_, 5 mmol (10 equiv) of TMSBn (**2a**), and 2.5 mmol (5 equiv) of TFA in 1,2-DCE (4.6 mL). ^b^Yield was determined by NMR analysis using 1,1,2,2-tetrabromoethane as an internal standard. Substrates **1** were not recovered in all cases. All products **3** were isolated before structural assignment. ^c^Batch reaction conditions (see ref. [55]): Unless otherwise noted, all reactions were carried out using blue LED (λ_max_ = 448 nm), 0.1 mmol of **1**, 1.0 mmol (10 equiv) of TMSBn (**2a**), 10 μmol (10 mol %) of AgNTf_2_, 20 μmol (20 mol %) of P(4-CF_3_C_6_H_4_)_3_, and 5 equiv of TFA in 1,2-DCE (1.0 mL: 0.1 M of **1**) at 50 °C for 1 h. ^d^At 0 °C for 6 h. ^e^The flow photochemical reactor having a 1.1 mL premixing zone using a dual syringe system with a flow rate of 6 mL/h (3 mL/h for each syringe).

Next, the effects of a carbonyl substituent, instead of a methyl ketone substituent, were investigated (Table 3). First, the reaction was performed with phenyl ketone **1m**, but product **3m** was obtained in low yield (Table 3, entry 1: 7%). This yield was lower than that obtained in the batch reaction (30%), and because 28% of **1m** were recovered, the flow reaction conditions were further examined (see Supporting Information File 1 for details). Although the yield of **3m** was improved to 19% (Table 3, entry 2) by increasing the temperature of the premixing zone from room temperature to 50 °C and reducing the flow rate from 24 mL/h to 6 mL/h (light irradiation time was extended from 2.5 min to 10 min), it did not exceed the yield of the batch reaction. In contrast, aldehyde **1n** having a simple phenyl group gave product **3n** in good yield (Table 3, entry 3: 72%). Because the yield of this flow reaction was better than that of the batch reaction (65%), the reactions of aldehydes with a series of substituents introduced to the terminal phenyl group were further investigated (Table 3, entries 4–7). Aldehydes having an electron-donating methyl group and an electron-withdrawing bromo group at the *para*-position of the phenyl moiety gave products **3o** and **3p**, respectively, in high yields (Table 3, entries 4 and 5). The aldehyde **1q** bearing a strong electron-withdrawing trifluoromethyl group at the *para*-position gave product **3q** in moderate yield (Table 3, entry 6: 63%), with the recovery of substrate **1q** (18%). The reaction of *ortho*-methyl-substituted aldehyde **1r** afforded the product **3r** in high yield when the temperature of the premixing zone was increased to 50 °C (Table 3, entry 7: 73%).

**Table 3:** Sequential transformation of phenyl ketone **1m** and aldehydes **1n**–**r**^a^.

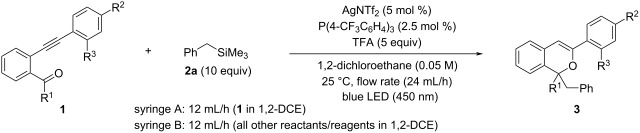								

Entry	**1**	R^1^	R^2^	R^3^	**3**	Yield of **3** (%)^b^	Recovery of **1** (%)^b^	Batch reaction using AgNTf_2_. Yield of **3** (%)^c^

1	**1m**	Ph	H	H	**3m**	7	29	30
2^d,e^	**1m**				**3m**	19	0	
3	**1n**	H	H	H	**3n**	72	0	65^f^
4	**1o**	H	Me	H	**3o**	82	5	–
5	**1p**	H	Br	H	**3p**	75	0	34^f^
6	**1q**	H	CF_3_	H	**3q**	63	18	–
7^e^	**1r**	H	H	Me	**3r**	73	8	–

^a^Unless otherwise noted, all reactions were carried out in the flow photochemical reactor (volume: 1.0 mL, λ_max_ = 450 nm) having a 0.7 mL premixing zone using a dual syringe system with a flow rate of 24 mL/h (12 mL/h for each syringe) at 25 °C. Syringe A: 0.5 mmol of **1** in 1,2-DCE (5.4 mL). Syringe B: 25 μmol (5 mol %) of AgNTf_2_, 12.5 μmol (2.5 mol %) of P(4-CF_3_C_6_H_4_)_3_, 5 mmol (10 equiv) of TMSBn (**2a**), and 2.5 mmol (5 equiv) of TFA in 1,2-DCE (4.6 mL). ^b^Yield was determined by NMR analysis using 1,1,2,2-tetrabromoethane as an internal standard. All products **3** were isolated before structural assignment. ^c^Batch reaction conditions: unless otherwise noted, reactions were carried out using 0.1 mmol of **1**, 1.0 mmol (10 equiv) of TMSBn (**2a**), 10 μmol (10 mol %) of AgNTf_2_, 20 μmol (20 mol %) of P(4-CF_3_C_6_H_4_)_3_, and 5 equiv of TFA in 1,2-DCE (1 mL: 0.1 M of **1**) at 50 °C for 1 h. ^d^The flow photochemical reactor having a 0.5 mL premixing zone using a dual syringe system with a flow rate of 6 mL/h (3 mL/h for each syringe). ^e^The temperature of the premixing zone was increased to 50 °C. ^f^The reaction was performed using 0.05 M of **1** and 2.5 equiv of TFA for 2 h.

**Scheme 2:**
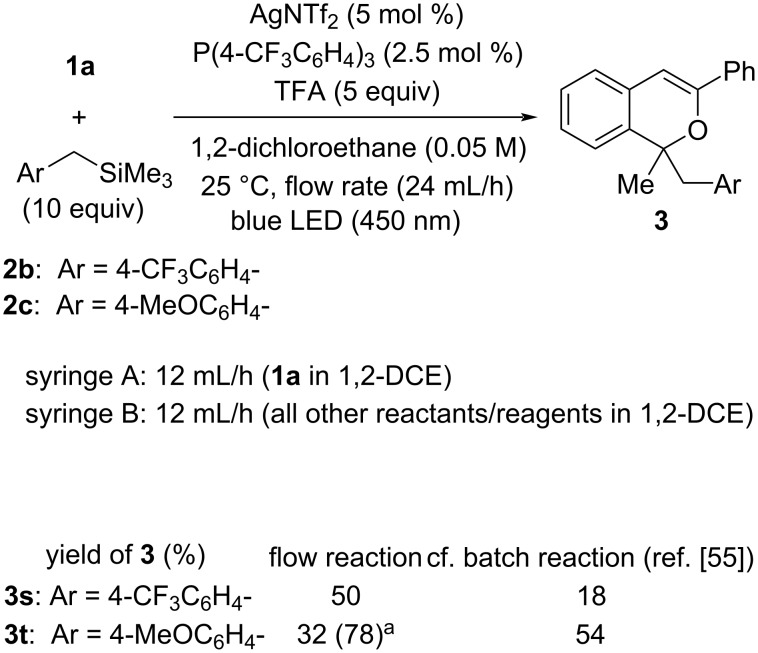
The reaction with benzyltrimethylsilane derivatives **2**. ^a^Flow rate was 6 mL/h and premixing zone was 0.2 mL.

The scope of donor molecules **2b** and **2c** having an electron-withdrawing trifluoromethyl group and an electron-donating methoxy group [60–61] at the *para*-position of the benzyltrimethylsilane, respectively, was also investigated in the present flow reaction system (Scheme 2). As expected, the flow reaction of **2b** having a trifluoromethyl group afforded product **3s** in higher yield (50%) than that of the batch reaction (18%) under the optimal reaction conditions. In contrast, in the flow reaction of **2c** having a methoxy group, product **3t** was obtained in a markedly lower yield (32%) than that of the batch reaction (54%). However, extending the light irradiation time by reducing the flow rate from 24 mL/h to 6 mL/h (light irradiation time: 24 mL/h = 2.5 min, 6 mL/h = 10 min) significantly improved the yield of **3t** (78%), presumably because of the retardation of the desilylation process (from **B** to **C** in Scheme 1a).

## Conclusion

We have demonstrated a flow reaction system for a π-Lewis acidic metal-catalyzed cyclization/photochemical radical addition sequence, affording, in most cases, the 1*H*-isochromene derivatives in higher yields than the batch reaction system, even with the amount of the π-Lewis acidic metal catalyst reduced by half. In the present sequential transformation, the key cationic species, 2-benzopyrylium intermediates, were generated in situ through the AgNTf_2_-catalyzed intramolecular cyclization of *ortho*-carbonyl alkynylbenzene derivatives and subsequent proto-demetalation with TFA. Further photoreactions of 2-benzopyrylium intermediates with benzyltrimethylsilane derivatives as the donor molecule were conducted in the flow photoreactor. We confirmed that the flow reaction system is an excellent method for improving the efficiency of the present sequential transformation, avoiding product degradation under photochemical reaction conditions. Further investigation of other flow photochemical reactions using in situ-generated organic cations is in progress in our laboratory.

## Supporting Information

File 1 The exploratory investigation, experimental procedures, and characterization data.

File 2 Copies of NMR spectra.

## Data Availability

All data that supports the findings of this study is available in the published article and/or the supporting information to this article.

## References

[1] Ehrfeld W, Hessel V, Löwe H (2000). Microreactors.

[2] Yoshida J (2015). Basics of Flow Microreactor Synthesis.

[3] Noël T (2016). Organometallic Flow Chemistry.

[4] Mason B P, Price K E, Steinbacher J L, Bogdan A R, McQuade D T (2007). Chem Rev.

[5] Ahmed-Omer B, Brandt J C, Wirth T (2007). Org Biomol Chem.

[6] Watts P, Wiles C (2007). Chem Commun.

[7] McMullen J P, Jensen K F (2010). Annu Rev Anal Chem.

[8] Yoshida J, Kim H, Nagaki A (2011). ChemSusChem.

[9] Wiles C, Watts P (2012). Green Chem.

[10] Kirschning A, Kupracz L, Hartwig J (2012). Chem Lett.

[11] McQuade D T, Seeberger P H (2013). J Org Chem.

[12] Elvira K S, i Solvas X C, Wootton R C R, deMello A J (2013). Nat Chem.

[13] Pastre J C, Browne D L, Ley S V (2013). Chem Soc Rev.

[14] Cambié D, Bottecchia C, Straathof N J W, Hessel V, Noël T (2016). Chem Rev.

[15] Plutschack M B, Pieber B, Gilmore K, Seeberger P H (2017). Chem Rev.

[16] Buglioni L, Raymenants F, Slattery A, Zondag S D A, Noël T (2022). Chem Rev.

[17] Nagaki A, Kim H, Yoshida J (2008). Angew Chem, Int Ed.

[18] Kim H, Min K-I, Inoue K, Im D J, Kim D-P, Yoshida J (2016). Science.

[19] Seo H, Katcher M H, Jamison T F (2017). Nat Chem.

[20] Otake Y, Nakamura H, Fuse S (2018). Angew Chem, Int Ed.

[21] Nagaki A, Yamashita H, Hirose K, Tsuchihashi Y, Yoshida J (2019). Angew Chem, Int Ed.

[22] Nagaki A, Ichinari D, Yoshida J (2013). Chem Commun.

[23] Moon S-Y, Jung S-H, Bin Kim U, Kim W-S (2015). RSC Adv.

[24] Degennaro L, Maggiulli D, Carlucci C, Fanelli F, Romanazzi G, Luisi R (2016). Chem Commun.

[25] Nauth A M, Lipp A, Lipp B, Opatz T (2017). Eur J Org Chem.

[26] Tucker J W, Zhang Y, Jamison T F, Stephenson C R J (2012). Angew Chem, Int Ed.

[27] Hernandez‐Perez A C, Collins S K (2013). Angew Chem, Int Ed.

[28] Elliott L D, Knowles J P, Koovits P J, Maskill K G, Ralph M J, Lejeune G, Edwards L J, Robinson R I, Clemens I R, Cox B (2014). Chem – Eur J.

[29] Talla A, Driessen B, Straathof N J W, Milroy L-G, Brunsveld L, Hessel V, Noël T (2015). Adv Synth Catal.

[30] Asao N, Nogami T, Takahashi K, Yamamoto Y (2002). J Am Chem Soc.

[31] Yao T, Zhang X, Larock R C (2004). J Am Chem Soc.

[32] Patil N T, Yamamoto Y (2008). Chem Rev.

[33] Rudolph M, Hashmi A S K (2011). Chem Commun.

[34] Shiroodi R K, Gevorgyan V (2013). Chem Soc Rev.

[35] Saito K, Kajiwara Y, Akiyama T (2013). Angew Chem, Int Ed.

[36] Obradors C, Echavarren A M (2014). Chem Commun.

[37] Terada M, Li F, Toda Y (2014). Angew Chem, Int Ed.

[38] Dorel R, Echavarren A M (2015). Chem Rev.

[39] Debrouwer W, Heugebaert T S A, Roman B I, Stevens C V (2015). Adv Synth Catal.

[40] Asiri A M, Hashmi A S K (2016). Chem Soc Rev.

[41] Lauder K, Toscani A, Scalacci N, Castagnolo D (2017). Chem Rev.

[42] Zhang Z, Smal V, Retailleau P, Voituriez A, Frison G, Marinetti A, Guinchard X (2020). J Am Chem Soc.

[43] Raj A S K, Narode A S, Liu R-S (2021). Org Lett.

[44] Greiner L C, Arichi N, Inuki S, Ohno H (2023). Angew Chem, Int Ed.

[45] Das S (2023). Asian J Org Chem.

[46] Beatty J W, Douglas J J, Cole K P, Stephenson C R J (2015). Nat Commun.

[47] Nakajima K, Miyake Y, Nishibayashi Y (2016). Acc Chem Res.

[48] Ermanis K, Colgan A C, Proctor R S J, Hadrys B W, Phipps R J, Goodman J M (2020). J Am Chem Soc.

[49] Xiong T, Zhang Q (2021). Chem Soc Rev.

[50] Alfonzo E, Hande S M (2020). ACS Catal.

[51] Kikuchi J, Kodama S, Terada M (2021). Org Chem Front.

[52] Schlegel M, Qian S, Nicewicz D A (2022). ACS Catal.

[53] Takemura N, Sumida Y, Ohmiya H (2022). ACS Catal.

[54] Miura T, Yoritate M, Hirai G (2023). Chem Commun.

[55] Terada M, Yazaki R, Obayashi R, Iwasaki Z, Umemiya S, Kikuchi J (2024). Chem Sci.

[56] Prier C K, Rankic D A, MacMillan D W C (2013). Chem Rev.

[57] Romero N A, Nicewicz D A (2016). Chem Rev.

[R58] 58Cu(NTf_2_)_2_ which was also the effective catalyst in our batch system (ref. [55]) did not solve well in 1,2-DCE.

[R59] 59Benzyltrimethylsilane (**2a**), which was not consumed in the corresponding radical reaction, was almost completely recovered.

[60] Maruyama T, Mizuno Y, Shimizu I, Suga S, Yoshida J (2007). J Am Chem Soc.

[61] Montanaro S, Ravelli D, Merli D, Fagnoni M, Albini A (2012). Org Lett.

