# Supplementary material for: Development of a flow photochemical process for a π-Lewis acidic metal-catalyzed cyclization/radical addition sequence: in situ-generated 2-benzopyrylium as photoredox catalyst and reactive intermediate
Source: Beilstein J Org Chem. 2024 Aug 13;20:1973–80. doi: 10.3762/bjoc.20.173 (PMC11331546; doi:10.3762/bjoc.20.173)
Supplement: File 1 — The exploratory investigation, experimental procedures, and characterization data. [file Beilstein_J_Org_Chem-20-1973-s001.pdf]

BEILSTEIN JOURNAL OF ORGANIC CHEMISTRY

Supporting Information

for

Development of a flow photochemical process for a π-Lewis acidic metal-catalyzed cyclization/radical addition sequence: in situ-generated 2-benzopyrylium as photoredox catalyst and reactive intermediate

Masahiro Terada, Zen Iwasaki, Ryohei Yazaki, Shigenobu Umemiya and Jun Kikuchi

Beilstein J. Org. Chem. **2024,** *20,* 1973–1980. doi:10.3762/bjoc.20.173

The exploratory investigation, experimental procedures, and characterization data

License and Terms: This is a supporting information file under the terms of the Creative Commons Attribution License (https://creativecommons.org/licenses/by/4.0). Please note that the reuse, redistribution and reproduction in particular requires that the author(s) and source are credited and that individual graphics may be subject to special legal provisions.
The license is subject to the *Beilstein Journal of Organic Chemistry* terms and conditions: (https://www.beilstein-journals.org/bjoc/terms)

Table of contents

1. General information S2

2. Preparation of *ortho*-carbonyl alkynylbenzene derivatives **1** S3

3. Typical procedures for the flow photochemical reaction S8

4. Screening of reaction conditions S9

5. Analytical data of products **3** S13

6. Catalytic cycles of the sequential transformation and theoretical studies S20

7. References S26

1. General information

Unless otherwise noted, all reactions were carried out under nitrogen or argon atmosphere in flame-dried glassware. Anhydrous 1,2-dichloroethane ($C_2H_4Cl_2$) was purchased from Aldrich. Other solvents and reagents were purchased from commercial suppliers and used without further purification. The flow photo reactions were carried out in photo-microreactor system KeyChem-Basic series provided by YMC Co., Ltd. The syringe pump was a YSP-201 and the thermal control unit was a stand-alone Thermos Stage TS-C-R. The blue light source was HCK1012-01-002 (450 PF: λ_{max} = 450 nm) provided by HepatoChem, Inc. These were connected each other with tubing (YMC-P-0025, inner diameter 0.5 mm). The batch reactions performed for comparison were conducted using Techno Sigma-PER-AMPs (λ_{max} = 448 nm light source). Purification of products was carried out by flash column chromatography using silica gel 60 N (spherical, neutral, 40–50 μm; Kanto Chemical Co., Inc.). Analytical thin layer chromatography (TLC) was performed on Merck pre-coated TLC plates (silica gel 60 GF 254, 0.25 mm). ^{1}H NMR spectra were recorded on a JEOL ECA-600 (600 MHz) spectrometer. Chemical shifts are reported in ppm from tetramethylsilane or solvent resonance as the internal standard ($CHCl_3$: 7.26 ppm, TMS: 0.00 ppm). $^{13}C\{^{1}H\}$ NMR spectra were recorded on a JEOL ECA-600 (151 MHz) spectrometer with complete proton decoupling. Chemical shifts are reported in ppm from the solvent resonance as the internal standard ($CDCl_3$: 77.0 ppm). NMR yields were determined using $C_2H_2Br_4$ as the internal standard. Infrared spectra were recorded on a Jasco FT/IR-4100 spectrometer. Mass spectral analysis using the APCI ionization method was performed on a Bruker Daltonics SolariX 12T spectrometer and the FD method was performed using a JEOL JMS-T100GC spectrometer at the Macromolecule Research Center, Graduate School of Science, Tohoku University.

2. Preparation of *ortho*-carbonyl alkynylbenzene derivatives **1**

Preparation of **S3**

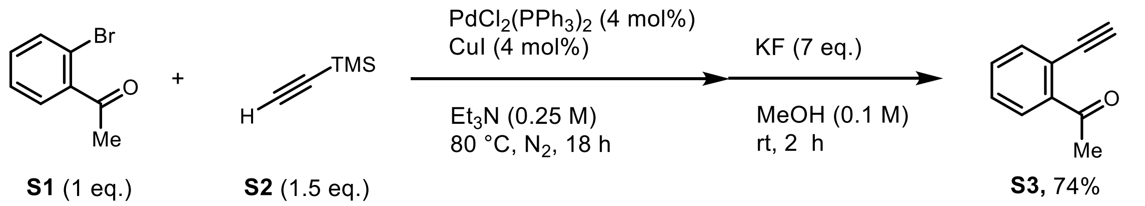

To an oven-dried three-neck flask with a magnetic stirrer bar were added $PdCl_2(PPh_3)_2$ (276.4 mg, 4 mol %) and CuI (75.7 mg, 4 mol %). The atmosphere was replaced with nitrogen, then 2-bromoacetophenone (**S1**, 1.85 g, 1.16 mL, 10 mmol), trimethylsilylacetylene (**S2**, 1.5 g, 2.1 mL, 15 mmol), and triethylamine (40 mL) were added to the mixture, and the reaction mixture was stirred for 18 hours at 80 °C (oil bath). The reaction was quenched with aq. NH_4Cl. The resulting mixture was extracted with EtOAc, and the combined extracts were washed with H_2O and brine, dried over Na_2SO_4, and concentrated under reduced pressure after filtration. The crude product was used in the next reaction without further purification.

To the crude product was added KF (4.1g, 70 mmol) and MeOH (100 mL), and the reaction mixture was stirred for 2 h at room temperature. The reaction was quenched with water. The resulting mixture was extracted with EtOAc, and the combined extracts were washed with brine, dried over Na_2SO_4, and concentrated under reduced pressure after filtration. The residual crude product was purified by column chromatography on silica gel (hexane/EtOAc 30:1 to 20:1) to give the product **S3** as a red oil (959.1 mg, 74% yield).

Preparation of *ortho*-alkynylmethylketone **1**

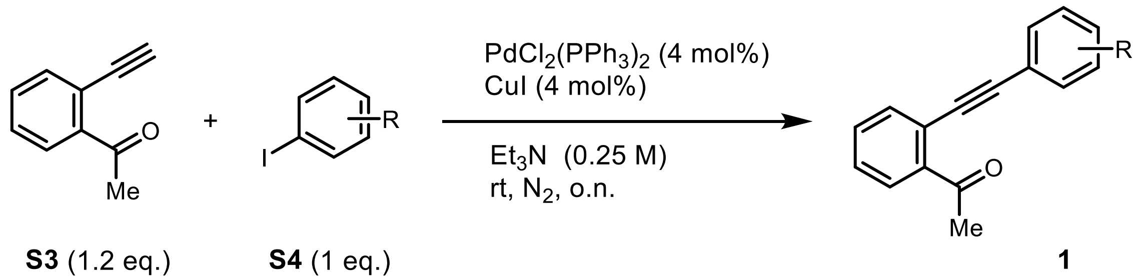

General procedure: To an oven-dried two-neck flask with a magnetic stirrer bar were added $PdCl_2(PPh_3)_2$ (4 mol %) and CuI (4 mol %). The atmosphere was replaced with nitrogen, then 2-ethynylacetophenone (**S3**, 1.5 mmol), iodobenzene derivative **S4** (1.25 mmol) and triethylamine (5 mL) were added to the mixture, and the reaction mixture was stirred overnight at room temperature. The reaction was quenched with aq. NH_4Cl. The resulting mixture was extracted with EtOAc, and the combined extracts were washed with aq. NH_4Cl, H_2O, and brine, dried over Na_2SO_4, and concentrated under reduced pressure after filtration. The residual crude product was purified by column chromatography on silica gel (hexane/EtOAc) to give product **1**.

1-(2-(Phenylethynyl)phenyl)ethan-1-one (**1a**)

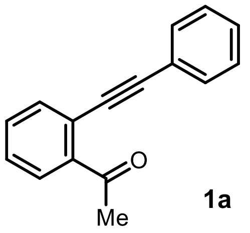

1a

98% yield; orange oil; hexane/EtOAc = 50:1 to 20:1

All spectroscopic data for **1a** (^{1}H NMR, ^{13}C{^{1}H} NMR, IR, and HRMS) were identical to the known compound.[1]

1-(2-((4-Methoxyphenyl)ethynyl)phenyl)ethan-1-one (**1b**)

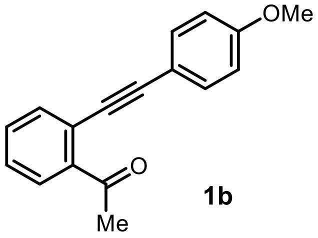

1b

96% yield; orange oil; hexane/EtOAc = 30:1 to 5:1

All spectroscopic data for **1b** (^{1}H NMR, ^{13}C{^{1}H} NMR, IR, and HRMS) were identical to the known compound.[1]

1-(2-(*p*-Tolylethynyl)phenyl)ethan-1-one (**1c**)

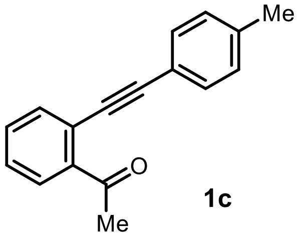

1c

82% yield; orange solid; hexane/EtOAc = 30:1 to 5:1

All spectroscopic data for **1c** (^{1}H NMR, ^{13}C{^{1}H} NMR, IR, and HRMS) were identical to the known compound.[1]

1-(2-((4-Bromophenyl)ethynyl)phenyl)ethan-1-one (**1d**)

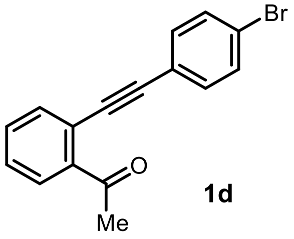

1d

86% yield; yellow solid; hexane/EtOAc = 30:1 to 10:1

All spectroscopic data for **1d** (^{1}H NMR, ^{13}C{^{1}H} NMR, IR, and HRMS) were identical to the known compound.[2]

1-(2-((4-(Trifluoromethyl)phenyl)ethynyl)phenyl)ethan-1-one (**1e**)

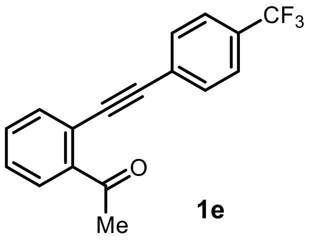

1e

89% yield; orange oil; hexane/EtOAc = 25:1 to 10:1

All spectroscopic data for **1e** (^{1}H NMR, ^{13}C{^{1}H} NMR, IR, and HRMS) were identical to the known compound.[1]

1-(2-((3-Methoxyphenyl)ethynyl)phenyl)ethan-1-one (**1f**)

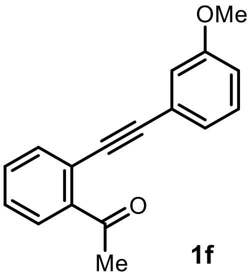

1f

90% yield; orange oil; hexane/EtOAc = 25:1 to 10:1

All spectroscopic data for **1f** (^{1}H NMR, ^{13}C{^{1}H} NMR, IR, and HRMS) were identical to the known compound.[3]

1-(2-(*m*-Tolylethynyl)phenyl)ethan-1-one (**1g**)

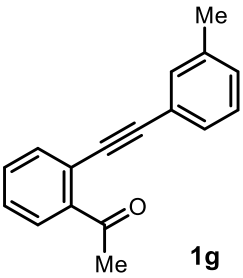

1g

95% yield; yellow oil; hexane/EtOAc = 30:1 to 5:1

All spectroscopic data for **1g** (^{1}H NMR, ^{13}C{^{1}H} NMR, IR, and HRMS) were identical to the known compound.[2]

1-(2-((3-Bromophenyl)ethynyl)phenyl)ethan-1-one (**1h**)

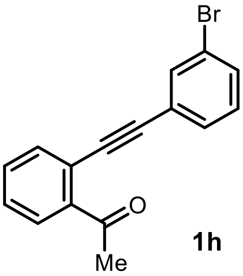

1h

88% yield; orange oil; hexane/EtOAc = 25:1 to 10:1

All spectroscopic data for **1h** (^{1}H NMR, ^{13}C{^{1}H} NMR, IR, and HRMS) were identical to the known compound.[2]

1-(2-((3-(Trifluoromethyl)phenyl)ethynyl)phenyl)ethan-1-one (**1i**)

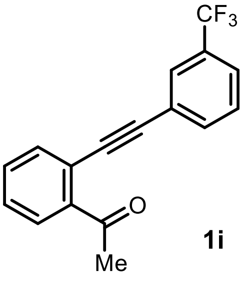

1i

92% yield; orange oil; hexane/EtOAc = 25:1 to 5:1; R_f = 0.4 (hexane/EtOAc = 5/1); ^{1}H NMR (600 MHz, $CDCl_3$) δ 7.81 (s, 1H), 7.78 (dd, *J* = 8.3, 1.4 Hz, 1H), 7.73 (dd, *J* = 7.6 Hz, 1H), 7.65 (dd, *J* = 8.3, 1.4 Hz, 1H), 7.61 (d, *J* = 7.6 Hz, 1H), 7.52-7.48 (m, 2H), 7.44 (td, *J* = 7.6, 1.4 Hz, 1H), 2.76 (s, 3H); ^{13}C {^{1}H} NMR (151MHz, $CDCl_3$) δ 199.8, 140.6, 134.7, 134.1, 131.4, 131.0 (q, J_{C-F} = 31.8 Hz), 129.0, 128.9, 128.7, 128.2 (q, J_{C-F} = 3.9 Hz), 125.2 (q, J_{C-F} = 4.3 Hz), 123.9, 123.6 (q, J_{C-F} = 271.7 Hz), 121.0, 92.9, 89.9, 29.6; IR (neat): 3065, 2981, 2916, 1688, 1489, 1430, 1336, 1165, 1122, 1091, 1071, 892, 803, 759, 695 cm^{-1}; HRMS (FD) *m*/*z*: $[M]^+$ Calcd for $C_{17}H_{11}F_3O$ 288.0757; Found 288.0761.

1-(2-(*o*-Tolylethynyl)phenyl)ethan-1-one (**1j**)

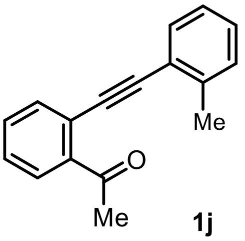

1j

89% yield; yellow oil; hexane/EtOAc = 25:1 to 5:1; R_f = 0.27 (hexane/EtOAc = 15/1); ^{1}H NMR (600 MHz, $CDCl_3$) δ 7.74 (d, J = 7.9 Hz, 1H), 7.64 (d, J = 7.6 Hz, 1H), 7.51 (d, J = 7.9 Hz, 1H), 7.47 (t, J = 7.6 Hz, 1H), 7.39 (t, J = 7.6 Hz, 1H), 7.31-7.27 (m, 2H), 7.20-7.17 (m, 1H), 2.78 (s, 3H), 2.54 (s, 3H); ^{13}C{^{1}H} NMR (151 MHz, $CDCl_3$) δ 200.3, 140.4, 140.3, 134.0, 131.9, 131.2, 129.6, 128.8, 128.6, 128.1, 125.6, 122.6, 121.9, 94.0, 92.1, 29.9, 20.7; IR (neat): 3060, 3021, 2921, 2861, 2209, 1922, 1830, 1686, 1591, 1561, 1488, 1356, 1278, 1244, 955, 795 cm^{-1}; HRMS (APCI) m/z: $[M + H]^+$ Calcd for $C_{17}H_{15}O$ 235.1117; Found 235.1118.

1-(4-Fluoro-2-(phenylethynyl)phenyl)ethan-1-one (**1k**)

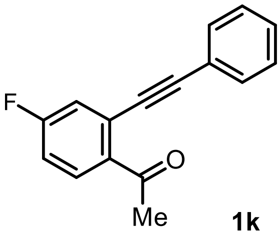

1k

78% yield; yellow oil; hexane/EtOAc = 25:1 to 10:1

All spectroscopic data for **1k** (^{1}H NMR, ^{13}C{^{1}H} NMR, IR, and HRMS) were identical to the known compound.[4]

1-(5-Fluoro-2-(phenylethynyl)phenyl)ethan-1-one (**1l**)

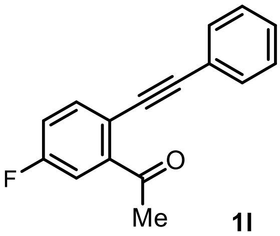

1l

80% yield; yellow oil; hexane/EtOAc = 25:1 to 10:1

All spectroscopic data for **1l** (^{1}H NMR, ^{13}C{^{1}H} NMR, IR, and HRMS) were identical to the known compound.[5]

Phenyl(2-(phenylethynyl)phenyl)methanone (**1m**)

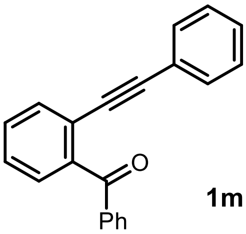

1m

92% yield; yellow oil; hexane/EtOAc = 30:1 to 5:1

All spectroscopic data for **1m** (^{1}H NMR, ^{13}C{^{1}H} NMR, IR, and HRMS) were identical to the known compound.[6]

2-(Phenylethynyl)benzaldehyde (**1n**)

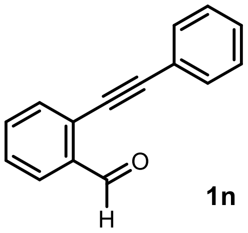

1n

58% yield; yellow oil; hexane/EtOAc = 30:1 to 5:1

All spectroscopic data for **1n** (^{1}H NMR, ^{13}C{^{1}H} NMR, IR, and HRMS) were identical to the known compound.[7]

2-(*p*-Tolylethynyl)benzaldehyde (**1o**)

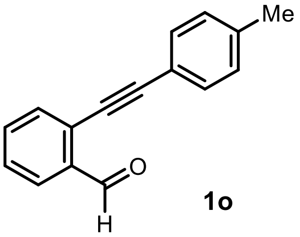

1o

70% yield; yellow solid; hexane/EtOAc = 25:1 to 10:1

All spectroscopic data for **1o** (^{1}H NMR, ^{13}C{^{1}H} NMR, IR, and HRMS) were identical to the known compound.[8]

2-((4-Bromophenyl)ethynyl)benzaldehyde (**1p)**

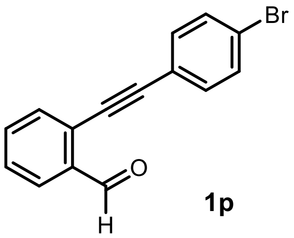

1p

82% yield; white solid; hexane/EtOAc = 25:1 to 10:1

All spectroscopic data for **1p** (^{1}H NMR, ^{13}C{^{1}H} NMR, IR, and HRMS) were identical to the known compound. [9]

2-((4-(Trifluoromethyl)phenyl)ethynyl)benzaldehyde (**1q**)

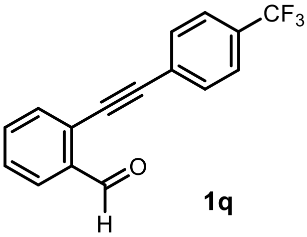

1q

65% yield; white solid; hexane/EtOAc = 25:1 to 10:1

All spectroscopic data for **1q** (^{1}H NMR, ^{13}C{^{1}H} NMR, IR, and HRMS) were identical to the known compound.[8]

2-(*o*-Tolylethynyl)benzaldehyde (**1r**)

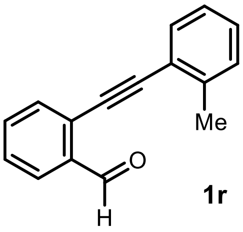

1r

76% yield; yellow solid; hexane/EtOAc = 25:1 to 5:1; R$_{f}$ = 0.33 (hexane/EtOAc = 15/1) ; Mp: 42.1-42.5 °C; ^{1}H NMR (600 MHz, CDCl$_3$) δ 10.68 (s, 1H), 7.96 (d, *J* = 7.9 Hz, 1H), 7.66 (d, *J* = 7.6 Hz, 1H), 7.61 (t, *J* = 7.6 Hz, 1H), 7.54 (d, *J* = 7.6 Hz, 1H), 7.46 (t, *J* = 7.6 Hz, 1H), 7.28 (m, 2H), 7.21 (t, *J* = 7.6 Hz, 1H), 2.54 (s, 3H); ^{13}C{^{1}H} NMR (151 MHz, CDCl$_3$) δ 191.6, 140.3, 135.6, 133.8, 133.3, 132.1, 129.6, 129.1, 128.5, 127.2, 127.1, 125.7, 122.1, 95.3, 88.7, 20.9; IR (neat): 3064, 2921, 2839, 2745, 2212, 1868, 1698, 1593, 1490, 1265, 1193, 795, 760 cm^{-1}; HRMS (APCI) *m*/*z*: [M + H]$^+$ Calcd for $C_{16}H_{13}O$ 221.0961; Found 221.0961.

3. Typical procedures for the flow photochemical reaction

General procedure

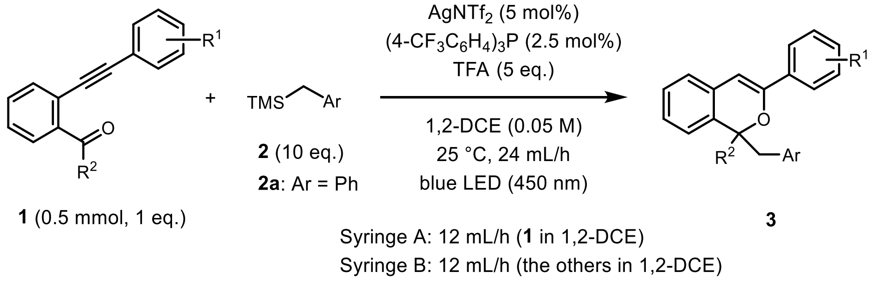

To a dried two-neck flask were added $AgNTf_2$ (9.7 mg, 5 mol %) and $(4\text{-}CF_3C_6H_4)_3P$ (5.9 mg, 2.5 mol%). Then, 1,2-dichloroethane (4.6 mL), benzyltrimethylsilane (**2a**, 690 μL, 5 mmol), and TFA (190 μL, 2.5 mmol) were added to the mixture. The resulting mixture was filled into syringe B. On the other hand, the solution of *ortho*-carbonyl alkynylbenzene derivative **1** (0.5 mmol) in 1,2-dichloroethane (5.4 mL) was filled into syringe A. Each syringe was set into a syringe pump and the flow rate of each syringe pump was set to half the flow rate in the reactor. This was due to achieve the indicated flow rate in the reactor. A light source (blue LED: λ_{max} = 450 nm) was placed at the top of the flow reactor. Before starting the flow reaction, the flow reactor was filled with 1,2-dichloroethane solvent and then the flow reaction started under light irradiation. The solution discharged from the flow reactor was immediately quenched with $NaHCO_3$ solution (see Figures S1 and S2). After pumping the solution in the syringe, all the remaining solution in the flow reactor was drained using 1,2-dichloroethane while irradiating with light. After the solution was discharged, the collected mixture was separated from the aqueous layer, the aqueous layer was extracted with dichloromethane, and the combined organic layers were concentrated under reduced pressure. The residual crude product was purified by column chromatography on silica gel (pure hexane to hexane/EtOAc 30:1) to give product **3**.

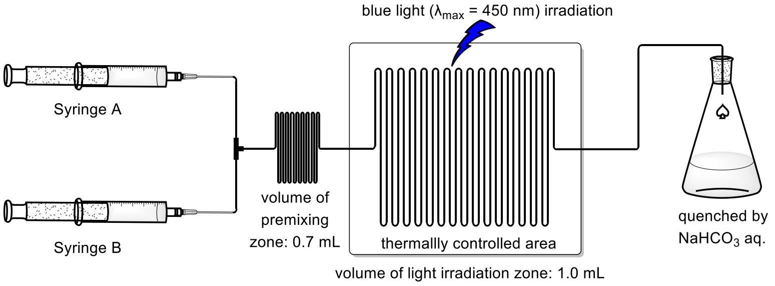

Figure S1. Flow system with premixing zone.

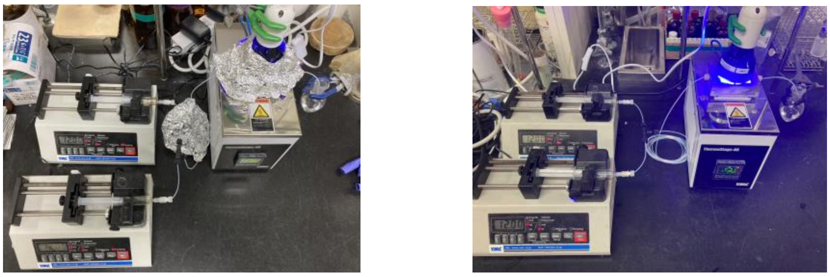

Figure S2. Photo of flow system with premixing zone.

4. Screening of reaction conditions

Since it is necessary to perform a π-Lewis acidic metal-catalyzed cyclization prior to the photoreaction, a method of separating the substrate, catalyst, and reagents into two syringes and mixing them before light irradiation was adopted. As shown in Table S1, several combinations were examined and the yield of **3a** was slightly lower when substrate **1a** and the silver catalyst were filled in the same syringe (entries 4 and 5) compared to the case where the substrate **1a** and the silver catalyst were filled in different syringes (entries 1–3). From these results, we decided to divide the substrate **1a** into syringe A and the others, i.e., the silver catalyst, phosphine ligand, **2a**, and TFA into syringe B, as shown in entry 1.

Table S1. Searching for the suitable combination of **1a**, **2a**, silver catalyst, phosphine ligand, and TFA when splitting into two syringes.

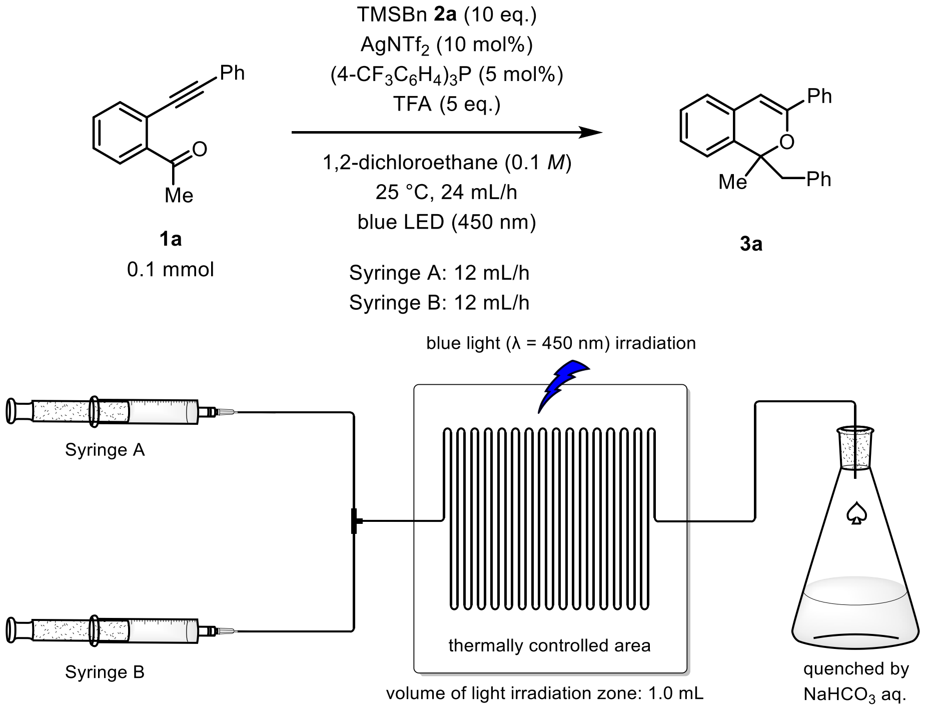

Entry	Syringe A	Syringe B	**3a** (%)[a]	Recovery of **1a** (%)[a]
1	**1a**	**2a**, $AgNTf_2$, $(4\text{-}CF_3C_6H_4)_3P$, TFA	47	0
2	**1a**, **2a**	$AgNTf_2$, $(4\text{-}CF_3C_6H_4)_3P$, TFA	42	0
3	**1a**, **2a**, TFA	$AgNTf_2$, $(4\text{-}CF_3C_6H_4)_3P$	49	0
4	**1a**, **2a**, $AgNTf_2$, $(4\text{-}CF_3C_6H_4)_3P$	TFA	32	0
5	**1a**, $AgNTf_2$, $(4\text{-}CF_3C_6H_4)_3P$	**2a**, TFA	38	2

[a]Determined by ^1H NMR analysis using 1,1,2,2-tetrabromoethane as an internal standard.

Table S2. Screening of flow reaction conditions

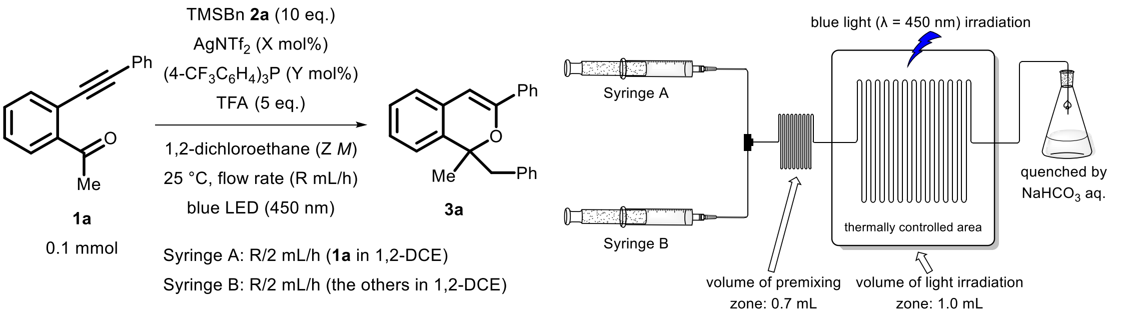

Entry	$AgNTf_2$ (X mol %)	$(CF_3C_6H_4)_3P$ (Y mol %)	Temp. (°C)	Conc. (Z, M)	Flow rate R (mL/h)	Premixing zone (mL)	**3a** (%)[a]	Rec. **1a** (%)[a]	Table 1 entry
1	10	20	50	0.1	3	none	42	0	entry 1
2	10	20	25	0.1	3	none	35	22	entry 2
3	10	5	25	0.1	3	none	53	1	entry 3
4	10	5	25	0.1	6	none	53	0	
5	10	5	25	0.1	24	none	53	0	entry 4
6	10	5	25	0.05	24	none	55	5	
7	5	2.5	25	0.1	24	none	26	14	entry 6
8	10	5	25	0.1	24	0.7	52	9	entry 5
9	10	5	25	0.1	6	0.7	52	1	
10	10	5	25	0.1	2	0.7	42	0	
11	5	2.5	25	0.1	24	0.7	49	0	entry 7
12	5	2.5	25	0.05	24	0.7	61	0	entry 8
13	2	1	25	0.05	24	0.7	28	28	entry 9
14	5	2.5	25	0.05	12	0.7	67	1	
15	5	2.5	25	0.05	6	0.7	57	2	
**16**[b]	**5**	**2.5**	**25**	**0.05**	**24**	**0.7**	**77**	**0**	**entry 10**
17[c]	5	2.5	25	0.05	24	0.7	75	0	
18[b]	5	2.5	25	0.05	24	none	62	3	
19[b]	5	2.5	25	0.05	24	1.1	73	0	
20[b]	5	2.5	25	0.05	24	2.2	67	0	
21[b]	5	2.5	25	0.05	96	1.1	36	0	

[a]Yield was determined by NMR analysis using 1,1,2,2-tetrabromoethane as an internal standard. [b]The reaction was conducted on 0.5 mmol scale. [c]The reaction was conducted on 1.0 mmol scale.

Table S3. Screening of reaction conditions in the reaction of **1b** (*p*-OMe substrate).

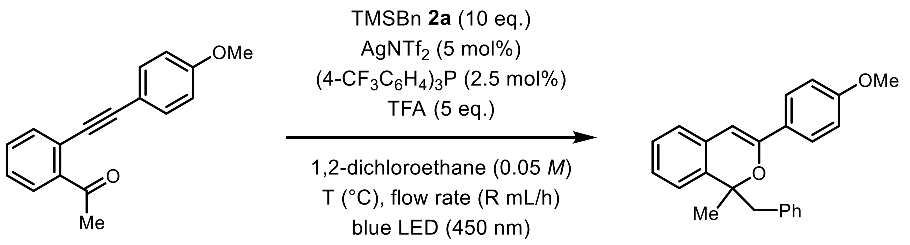

1b
0.5 mmol

Syringe A: R/2 mL/h (**1b** in 1,2-DCE)
Syringe B: R/2 mL/h (the others in 1,2-DCE)

3b

Entry	T (°C)	Flow rate	Premixing zone	Premixing time (min)	Irradiation time (min)	**3b** (%)[a]	Table 2 entry
1	25	24 mL/h	0.7 mL	1.7	2.5	14	entry 1
2	25	48 mL/h	1.4 mL	1.7	1.3	12	
3	25	96 mL/h	1.4 mL	0.8	0.6	23	
4	0	24 mL/h	0.7 mL	1.7	2.5	19	
5	0	12 mL/h	0.7 mL	3.3	5	16	
6	25	12 mL/h	0.7 mL	3.3	5	27	
7	50	12 mL/h	0.7 mL	3.3	5	28	
8	25	12 mL/h	1.1 mL	5.5	5	37	
9	25	24 mL/h	2.2 mL	5.5	2.5	20	
10	25	12 mL/h	2.2 mL	11	5	42	
11	25	6 mL/h	1.1 mL	11	10	54	entry 2
12	25	3 mL/h	1.1 mL	22	20	44	
13	25	12 mL/h	4.4 mL	22	5	40	

[a] Determined by ^{1}H NMR analysis using 1,1,2,2-tetrabromoethane as an internal standard. Substrate **1b** was completely consumed in all cases.

Table S4. Screening of reaction conditions in the reaction of **1m** (phenyl ketone substrate).

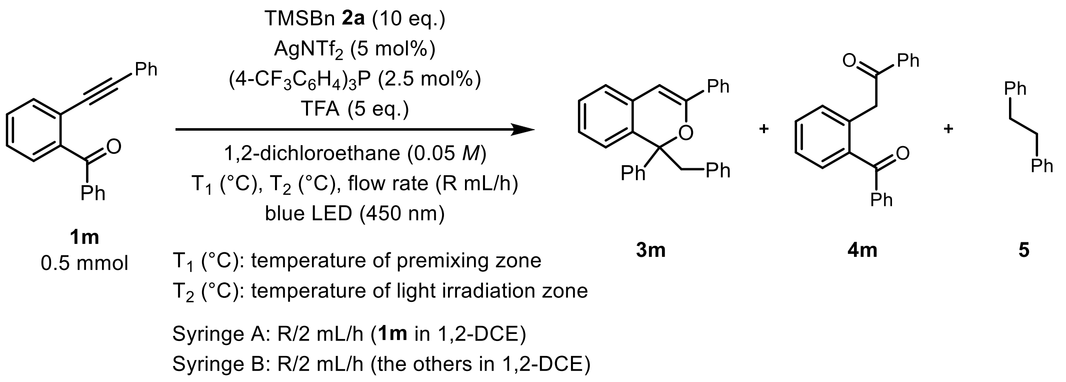

Entry	T_1 (°C)	T_2 (°C)	Flow rate	Premixing zone	Premixing time (min)	Irradiation time (min)	**3m**[a]	**1m**[a]	**4m**[a]	**5**[a]	Table 3 entry
1	rt	25	24 mL/h	10 m (2.2 mL)	1.7	2.5	7%	29%	0%	4%	entry 1
2	rt	25	24 mL/h	10 m (2.2 mL)	5.5	2.5	10%	23%	11%	2%	
3	rt	25	12 mL/h	5 m (1.1 mL)	5.5	5	5%	14%	0%	5%	
4	rt	5	12 mL/h	5 m (1.1 mL)	5.5	5	4%	19%	4%	6%	
5	50	25	24 mL/h	10 m (2.2 mL)	5.5	2.5	17%	0%	62%	0%	
6	35	25	24 mL/h	10 m (2.2 mL)	5.5	2.5	14%	0%	46%	1%	
7	50	25	6 mL/h	2.5 m (0.5 mL)	5.5	10	19%	0%	2%	7%	entry 2

[a] Determined by ^{1}H NMR analysis using 1,1,2,2-tetrabromoethane as an internal standard.

5. Analytical data of products **3**

1-Benzyl-1-methyl-3-phenyl-1*H*-isochromene (**3a**)

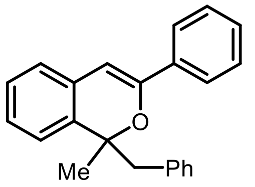

3a

77% yield; White foam; R_f = 0.59 (Hexane/EtOAc = 10/1); ^{1}H NMR (600 MHz, $CDCl_3$) δ 7.72 (d, *J* = 7.6 Hz, 2H), 7.39-7.32 (m, 3H), 7.22 (td, *J* = 7.6, 1.4 Hz, 1H), 7.19-7.15 (m, 3H), 7.12-7.09 (m, 2H), 6.96 (dd, *J* = 7.9, 1.4 Hz, 2H), 6.91 (d, *J* = 7.6 Hz, 1H), 6.47 (s, 1H), 3.22 (d, *J* = 13.8 Hz, 1H), 3.11 (d, *J* = 13.8 Hz, 1H), 1.71 (s, 3H); $^{13}C\{^1H\}$ NMR (151 MHz, $CDCl_3$) δ 150.5, 136.5, 134.6_3, 134.5_9, 131.1, 130.6, 128.6, 128.2, 127.7, 127.5, 126.3_1, 126.2_6, 125.0, 124.1, 123.7, 100.1, 80.8, 44.9, 24.4; IR (neat): 3061, 3028, 2980, 2922, 1723, 1698, 1495, 1452, 1272, 1235, 1089, 761, 700 cm^{-1}; HRMS (FD) *m*/*z*: $[M]^+$ Calcd for $C_{23}H_{20}O$ 312.1509; Found 312.1513.

1-Benzyl-3-(4-methoxyphenyl)-1-methyl-1*H*-isochromene (**3b**)

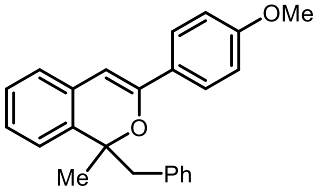

3b

54% yield; White foam; R_f = 0.57 (Hexane/EtOAc = 10/1); ^{1}H NMR (600 MHz, $CDCl_3$) δ 7.65 (dd, *J* = 6.9, 2.1 Hz, 2H), 7.21-7.18 (m, 4H), 7.10-7.07 (m, 2H), 6.96 (dd, *J* = 7.6, 1.7 Hz, 2H), 6.92-6.90 (m, 3H), 6.36 (s, 1H), 3.85 (s, 3H), 3.22 (d, *J* = 13.4 Hz, 1H), 3.08 (d, *J* = 13.4 Hz, 1H), 1.70 (s, 3H); $^{13}C\{^1H\}$ NMR (151 MHz, $CDCl_3$) δ 160.1, 150.4, 136.6, 134.4, 131.1, 130.9, 127.6, 127.5, 127.2, 126.5, 126.2, 125.9, 123.8, 123.6, 113.6, 98.5, 80.7, 55.3, 44.7, 24.3; IR (neat): 3061, 3026, 2934, 2835, 1509, 1248, 1173, 1055, 1028, 932, 833, 700 cm^{-1}; HRMS (FD) *m*/*z*: $[M]^+$ Calcd for $C_{24}H_{22}O_2$ 342.1614; Found 342.1619.

1-Benzyl-1-methyl-3-(*p*-tolyl)-1*H*-isochromene (**3c**)

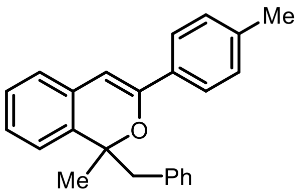

3c

76% yield; Colorless oil; R_f = 0.53 (Hexane/EtOAc = 10/1); ^{1}H NMR (600 MHz, $CDCl_3$) δ 7.61 (d, *J* = 8.2 Hz, 2H), 7.23-7.16 (m, 6H), 7.10-7.07 (m, 2H), 6.96 (d, *J* = 6.9 Hz, 2H), 6.90 (d, *J* = 7.6 Hz, 1H), 6.42 (s, 1H), 3.22 (d, *J* = 13.8 Hz, 1H), 3.10 (d, *J* = 13.8 Hz, 1H), 2.38 (s, 3H), 1.70 (s, 3H); $^{13}C\{^1H\}$ NMR (151 MHz, $CDCl_3$) δ 150.7, 138.6, 136.6, 134.6, 131.8, 131.1, 130.8, 128.9, 127.6, 127.5, 126.2, 126.1, 125.0, 124.0, 123.7, 99.3, 80.7, 44.8, 24.4, 21.3; IR (neat): 3061, 3027, 2981, 2919, 1452, 1273, 1082, 1057, 803, 748, 699 cm^{-1}; HRMS (FD) *m*/*z*: $[M]^+$ Calcd for $C_{24}H_{22}O$ 326.1665; Found 326.1670.

1-Benzyl-3-(4-bromophenyl)-1-methyl-1*H*-isochromene (**3d**)

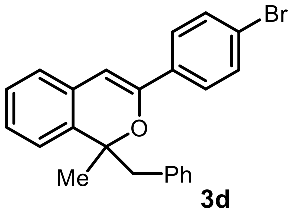

3d

57% yield; White foam; R$_f$ = 0.63 (Hexane/EtOAc = 10/1); ^{1}H NMR (600 MHz, $CDCl_3$) δ 7.56 (d, *J* = 8.6 Hz, 2H), 7.49 (d, *J* = 8.6 Hz, 2H), 7.24-7.16 (m, 4H), 7.15-7.10 (m, 2H), 6.95-6.93 (m, 3H), 6.45 (s, 1H), 3.21 (d, *J* = 13.8 Hz, 1H), 3.05 (d, *J* = 13.8 Hz, 1H), 1.70 (s, 3H); ^{13}C{^{1}H} NMR (151 MHz, $CDCl_3$) δ 149.5, 136.3, 134.7, 133.5, 131.3, 131.0, 130.3, 127.8, 127.6, 126.7, 126.5, 126.3, 124.3, 123.7, 122.6, 100.5, 80.9, 45.0, 24.4; IR (neat): 3062, 3026¸ 2983, 1629, 1486, 1451, 1398, 1054, 1005, 805, 746, 700 cm^{-1}; HRMS (FD) *m*/*z*: [M]$^+$ Calcd for $C_{23}H_{19}BrO$ 390.0614; Found 390.0618.

1-Benzyl-1-methyl-3-(4-(trifluoromethyl)phenyl)-1*H*-isochromene (**3e**)

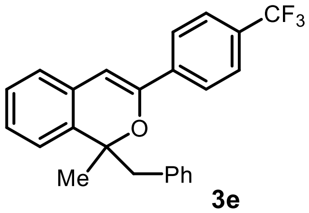

3e

77% yield; Colorless oil; R$_f$ = 0.57 (Hexane/EtOAc = 10/1); ^{1}H NMR (600 MHz, $CDCl_3$) δ 7.79 (d, *J* = 8.3 Hz, 2H), 7.61 (d, *J* = 8.6 Hz, 2H), 7.24 (td, *J* = 7.7, 1.4 Hz, 1H), 7.22-7.12 (m, 5H), 6.96 (d, *J* = 7.6 Hz, 1H), 6.94 (d, *J* = 6.9 Hz, 2H), 6.53 (s, 1H), 3.22 (d, *J* = 13.8 Hz, 1H), 3.08 (d, *J* = 13.8 Hz, 1H), 1.72 (s, 3H); ^{13}C{^{1}H} NMR (151 MHz, $CDCl_3$) δ 149.0, 138.0, 136.3, 134.9, 131.0, 130.1 (q, $J_{C\text{-}F}$ = 31.8 Hz), 130.0, 127.8, 127.6, 127.1, 126.4, 125.2 (q, $J_{C\text{-}F}$ = 4.3 Hz), 125.1 (q, $J_{C\text{-}F}$ = 271.5 Hz) 125.0, 124.6, 123.7, 101.9, 81.1, 45.1, 24.5; IR (neat): 3063, 3028, 2983, 1733, 1615, 1453, 1411, 1321, 1164, 1110, 1014, 851, 807, 750, 700 cm^{-1}; HRMS (FD) *m*/*z*: [M]$^+$ Calcd for $C_{24}H_{19}F_3O$ 380.1383; Found 380.1387.

1-Benzyl-3-(3-methoxyphenyl)-1-methyl-1*H*-isochromene (**3f**)

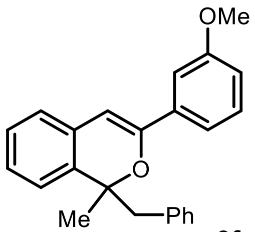

3f

39% yield; Colorless oil; R$_f$ = 0.57 (Hexane/EtOAc = 10/1); ^{1}H NMR (600 MHz, $CDCl_3$) δ 7.33 (dt, *J* = 7.8, 1.3 Hz, 1H), 7.29 (t, *J* = 7.9 Hz, 1H), 7.24-7.17 (m, 5H), 7.12-7.10 (m, 2H), 6.99-6.97 (m, 2H), 6.92 (d, *J* = 7.9 Hz, 1H), 6.89 (m, 1H), 6.46 (s, 1H), 3.82 (s, 3H), 3.23 (d, *J* = 13.4 Hz, 1H), 3.11 (d, *J* = 13.4 Hz, 1H), 1.69 (s, 3H); ^{13}C{^{1}H} NMR (151 MHz, $CDCl_3$) δ 159.6, 150.3, 136.5, 136.1, 134.8, 131.1, 130.6, 129.1, 127.7, 127.6, 126.4, 126.3, 124.2, 123.6, 117.5, 114.4, 110.3, 100.4, 80.8, 55.3, 44.8, 24.4; IR (neat): 3064,3030, 2937, 2834, 1748,1670,1647, 1630, 1604, 1542, 1508, 1489, 1452, 1429, 1322, 1229, 1171, 1061, 784, 719, 671, 658 cm^{-1}; HRMS (APCI) *m*/*z*: [M + H]$^+$ Calcd for $C_{24}H_{23}O$ 343.1693; Found 343.1693.

1-Benzyl-1-methyl-3-(*m*-tolyl)-1*H*-isochromene (**3g**)

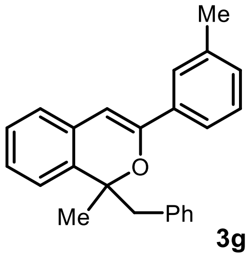

3g

75% yield; Colorless oil; R_f = 0.49 (Hexane/EtOAc = 10/1); ^{1}H NMR (600 MHz, $CDCl_3$) δ 7.53 (d, *J* = 7.6 Hz, 1H), 7.52 (s, 1H), 7.27 (t, *J* = 7.6 Hz, 1H), 7.23-7.15 (m, 5H), 7.12-7.09 (m, 2H), 6.97 (d, *J* = 6.5 Hz, 2H), 6.91 (d, *J* = 7.6 Hz, 1H), 6.46 (s, 1H), 3.22 (d, *J* = 13.8 Hz, 1H), 3.11 (d, *J* = 13.8 Hz, 1H), 2.38 (s, 3H), 1.71 (s, 3H); ^{13}C{^{1}H} NMR (151 MHz, $CDCl_3$) δ 150.6, 137.8, 136.6, 134.7, 134.5, 131.1, 130.7, 129.4, 128.1, 127.7, 127.5, 126.3, 126.2, 125.7, 124.1, 123.7, 122.2, 100.0, 80.7, 44.8, 24.4, 21.5; IR (neat): 3058, 3026, 2998, 2988, 2938, 2910, 1600, 1485, 1454, 1080, 1061, 1032, 808, 782, 767, 748, 716, 700, 691 cm^{-1}; HRMS (FD) *m*/*z*: $[M]^+$ Calcd for $C_{24}H_{22}O$ 326.1665; Found 326.1670.

1-Benzyl-3-(3-bromophenyl)-1-methyl-1*H*-isochromene (**3h**)

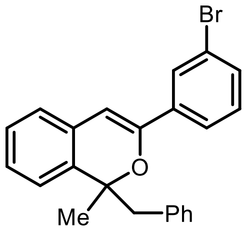

3h

68% yield; Yellow oil; R_f = 0.57 (Hexane/EtOAc = 10/1); ^{1}H NMR (600 MHz, $CDCl_3$) δ 7.83 (t, *J* = 1.9 Hz, 1H), 7.63 (d, *J* = 7.9 Hz, 1H), 7.45 (m, 1H), 7.25-7.17 (m, 5H), 7.15-7.11 (m, 2H), 6.96-6.93 (m, 3H), 6.46 (s, 1H), 3.21 (d, *J* = 13.7 Hz, 1H), 3.08 (d, *J* = 13.7 Hz, 1H), 1.71 (s, 3H); ^{13}C{^{1}H} NMR (151 MHz, $CDCl_3$) δ 149.0, 136.7, 136.3, 134.8, 131.4, 131.0, 130.2, 129.7, 128.0, 127.8, 127.6, 126.8, 126.4, 124.4, 123.7, 123.4, 122.6, 101.1, 81.0, 45.0, 24.4; IR (neat): 3062, 3027, 2981, 2917, 1557, 1485, 1474, 1452, 1240, 1079, 1058, 1031, 781, 746, 700, 685 cm^{-1}; HRMS (FD) *m*/*z*: $[M]^+$ Calcd for $C_{23}H_{19}BrO$ 390.0614; Found 390.0618.

1-Benzyl-1-methyl-3-(3-(trifluoromethyl)phenyl)-1*H*-isochromene (**3i**)

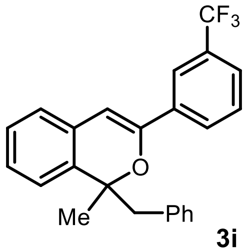

3i

65% yield; Colorless oil; R_f = 0.56 (Hexane/EtOAc = 10/1); ^{1}H NMR (600 MHz, $CDCl_3$) δ 7.92 (s, 1H), 7.87 (d, *J* = 7.9 Hz, 1H), 7.58 (d, *J* = 7.6 Hz, 1H), 7.49 (t, *J* = 7.7 Hz, 1H), 7.25-7.14 (m, 6H), 6.98-6.96 (m, 3H), 6.53 (s, 1H), 3.25 (d, *J* = 13.7 Hz, 1H), 3.07 (d, *J* = 13.7 Hz, 1H), 1.72 (s, 3H); ^{13}C{^{1}H} NMR (151 MHz, $CDCl_3$) δ 149.0, 136.2, 135.4, 135.0, 131.0, 130.7 (q, $J_{C\text{-}F}$ = 31.8 Hz), 130.0, 128.6, 127.9, 127.8, 127.6, 127.0, 126.4, 125.0 (q, $J_{C\text{-}F}$ = 4.3 Hz), 124.5, 124.1 (q, $J_{C\text{-}F}$ = 271.7 Hz), 123.7, 121.8 (q, $J_{C\text{-}F}$ = 4.3 Hz), 101.2, 81.1, 45.1, 24.4; IR (neat): 3064, 3028, 2984, 1487, 1450, 1333, 1164, 1121, 1072, 748, 722, 694 cm^{-1}; HRMS (FD) *m*/*z*: $[M]^+$ Calcd for $C_{24}H_{19}F_3O$ 380.1384; Found 380.1387.

1-Benzyl-1-methyl-3-(*o*-tolyl)-1*H*-isochromene (**3j**)

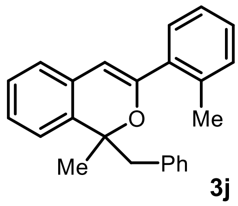

3j

76% yield; Colorless oil; R_f = 0.53 (Hexane/EtOAc = 10/1); ^{1}H NMR (600 MHz, $CDCl_3$) δ 7.47-7.45 (m, 1H), 7.25-7.19 (m, 4H), 7.17-7.11 (m, 3H), 7.06-7.03 (m, 2H), 6.88-6.86 (m, 2H), 6.76 (d, *J* = 7.6 Hz, 1H), 5.95 (s, 1H), 3.40 (d, *J* = 13.4 Hz, 1H), 3.15 (d, *J* = 13.4 Hz, 1H), 2.46 (s, 3H), 1.71 (s, 3H); ^{13}C{^{1}H} NMR (151 MHz, $CDCl_3$) δ 152.8, 136.7, 136.4, 135.5, 133.2, 130.9, 130.8, 130.8, 128.7, 128.4, 127.7, 127.5, 126.2, 126.0, 125.6, 124.1, 123.7, 103.8, 81.5, 45.1, 25.0, 21.1; IR (neat): 3060, 3023, 2979, 2926, 1720, 1701,1633,1601, 1486, 1453, 1341, 1249, 1158, 1118, 1056, 1034, 759, 723 cm^{-1}; HRMS (APCI) *m*/*z*: $[M + H]^+$ Calcd for $C_{24}H_{23}O$ 327.1743; Found 327.1743.

1-Benzyl-6-fluoro-1-methyl-3-phenyl-1*H*-isochromene (**3k**)

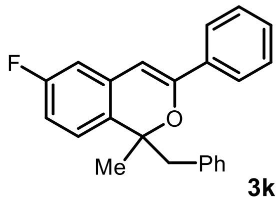

3k

79% yield; White foam; R_f = 0.56 (Hexane/EtOAc = 10/1); ^{1}H NMR (600 MHz, $CDCl_3$) δ 7.72-7.71 (m, 2H), 7.40-7.36 (m, 3H), 7.20-7.16 (m, 3H), 6.92 (d, *J* = 6.5 Hz, 2H), 6.81-6.75 (m, 3H), 6.39 (s, 1H), 3.18-3.13 (m, 2H), 1.71 (s, 3H). ^{13}C{^{1}H} NMR (151 MHz, $CDCl_3$) δ 162.4 (d, $J_{C\text{-}F}$ = 244.3 Hz), 151.8, 136.3, 134.2, 132.9 (d, $J_{C\text{-}F}$ = 8.6 Hz), 131.0, 130.0, 129.0, 128.3, 127.6, 126.4, 125.5 (d, $J_{C\text{-}F}$ = 8.6 Hz), 125.2, 112.5 (d, $J_{C\text{-}F}$ =21.7 Hz), 110.4 (d, $J_{C\text{-}F}$ =21.7 Hz), 99.4, 81.0, 44.9, 24.7; IR (neat): 3086, 3061, 3030, 2983, 2937, 1726, 1633, 1607, 1582, 1491, 1450, 1344, 1227, 1146, 1059, 964, 863, 760 cm^{-1}; HRMS (APCI) *m*/*z*: $[M + H]^+$ Calcd for $C_{23}H_{20}FO$ 331.1493; Found 331.1493.

1-Benzyl-7-fluoro-1-methyl-3-phenyl-1*H*-isochromene (**3l**)

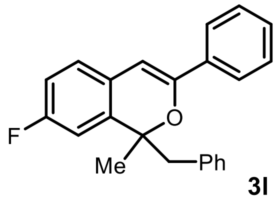

3l

75% yield; White foam; R_f = 0.56 (Hexane/EtOAc = 10/1);^{1}H NMR (600 MHz, $CDCl_3$) δ 7.70 (m, 2H), 7.39-7.34 (m, 3H), 7.21-7.17 (m, 3H), 7.07 (dd, *J* = 8.2, 5.8 Hz, 1H), 6.96-6.91 (m, 3H), 6.63 (dd, *J* = 9.5, 2.6 Hz, 1H), 6.44 (s, 1H), 3.19 (d, *J* = 13.7 Hz, 1H), 3.10 (d, *J* = 13.7 Hz, 1H), 1.69 (s, 3H). ^{13}C{^{1}H} NMR (151 MHz, $CDCl_3$) δ 161.2 (d, $J_{C\text{-}F}$ = 244.3 Hz), 149.8, 136.7 (d, $J_{C\text{-}F}$ = 7.2 Hz), 136.1, 134.4, 131.0, 128.7, 128.2, 127.7, 126.9 (d, $J_{C\text{-}F}$ = 3.0 Hz), 126.5, 125.5 (d, $J_{C\text{-}F}$ = 8.6 Hz), 124.9, 114.4 (d, $J_{C\text{-}F}$ = 21.7 Hz), 111.3 (d, $J_{C\text{-}F}$ = 24.6 Hz), 99.2, 80.5, 44.6, 24.3; IR (neat): 3086, 3061, 3030, 2984, 2937, 1948, 1879, 1633, 1607, 1583, 1496, 1449, 1346, 1271, 1061, 829, 761 cm^{-1}; HRMS (APCI) *m*/*z*: $[M + H]^+$ Calcd for $C_{23}H_{20}FO$ 331.1493; Found 331.1493.

1-Benzyl-1,3-diphenyl-1*H*-isochromene (**3m**)

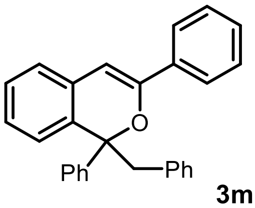
3m

19% yield; White solid; R_f = 0.63 (Hexane/EtOAc = 15/1); Mp: 155.4-156.8 °C; ^{1}H NMR (600 MHz, $CDCl_3$) δ 7.72 (dt, *J* = 6.8, 1.5 Hz, 2H), 7.50 (dd, *J* = 7.4, 1.5 Hz, 1H), 7.38-7.35 (m, 2H), 7.34-7.26 (m, 4H), 7.19-7.11 (m, 6H), 7.10-7.06 (m, 4H), 6.37 (s, 1H), 3.76 (d, *J* = 14.1 Hz, 1H), 3.69 (d, *J* = 14.1 Hz, 1H); ^{13}C{^{1}H} NMR (151 MHz, $CDCl_3$) δ 151.0, 143.0, 136.2, 134.2, 133.1, 131.5, 131.3, 128.6, 128.3, 128.0, 127.5, 127.4, 127.2, 126.3, 126.2, 126.2, 124.8, 124.6, 101.1, 83.9, 46.6; IR (neat): 3061, 3029, 2923, 2851, 1735, 1653, 1633, 1066, 1495, 1454, 1346, 1065, 1027, 794, 764 cm^{-1}; HRMS (APCI) *m*/*z*: $[M + H]^+$ Calcd for $C_{28}H_{23}O$ 375.1743; Found 375.1744.

1-Benzyl-3-phenyl-1*H*-isochromene (**3n**)

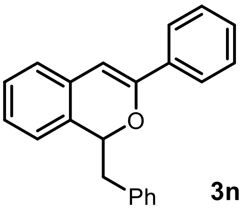
3n

72% yield; White solid; R_f = 0.63 (Hexane/EtOAc = 15/1); Mp: 103.9-104.8 °C; ^{1}H NMR (600 MHz, $CDCl_3$) δ 7.60-7.58 (m, 2H), 7.34-7.27 (m, 6H), 7.24 (m, 2H), 7.17-7.11 (m, 4H), 6.91-6.89 (m, 1H), 6.49 (s, 1H), 5.52 (dd, *J* = 9.0, 5.2 Hz, 1H), 3.31 (dd, *J* = 13.8, 9.0 Hz, 1H), 3.05 (dd, *J* = 13.8, 5.2 Hz, 1H); ^{13}C{^{1}H} NMR (151 MHz, $CDCl_3$) δ151.0, 137.5, 134.3, 130.8, 130.8, 129.8, 128.7, 128.2, 128.1, 126.4, 126.2, 125.1, 124.3, 124.0, 100.2, 79.1, 40.5; IR (neat): 3062, 3028, 2922, 2850, 1630, 1604, 1569, 1495, 1454, 1375, 1280, 1064, 1030, 795, 765 cm^{-1}; HRMS (APCI) *m*/*z*: $[M + H]^+$ Calcd for $C_{22}H_{19}O$ 299.1430; Found 299.1430.

1-Benzyl-3-(*p*-tolyl)-1*H*-isochromene (**3o**)

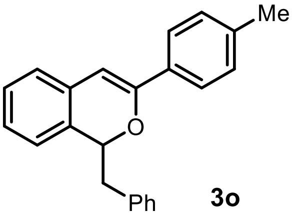
3o

82% yield; White solid; R_f = 0.56 (Hexane/EtOAc = 15/1); Mp: 122.2-123.2 °C; ^{1}H NMR (600 MHz, $CDCl_3$) δ 7.48 (d, *J* = 7.9 Hz, 2H), 7.30-7.22 (m, 4H), 7.17-7.09 (m, 6H), 6.89 (d, *J* = 7.9 Hz, 1H), 6.44 (s, 1H), 5.49 (dd, *J* = 9.1, 5.3 Hz, 1H), 3.30 (dd, *J* = 14.0, 9.1 Hz, 1H), 3.04 (dd, *J* = 14.0, 5.3 Hz, 1H), 2.36 (s, 3H); ^{13}C{^{1}H} NMR (151 MHz, $CDCl_3$) δ 151.2, 138.7, 137.6, 131.5, 130.9, 130.8, 129.8, 128.9, 128.2, 128.0, 126.4, 126.0, 125.1, 124.2, 123.8, 99.4, 79.1, 40.4, 21.3; IR (neat): 3064, 3029, 2945, 2917, 1626, 1604, 1510, 1488, 1454, 1375, 1275, 1183, 1064, 800, 751 cm^{-1}; HRMS (APCI) *m*/*z*: $[M + H]^+$ Calcd for $C_{23}H_{21}O$ 313.1587; Found 313.1587.

1-Benzyl-3-(4-bromophenyl)-1H-isochromene (**3p**)

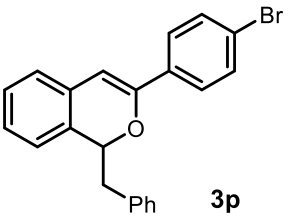

3p

75% yield; White solid; R_f = 0.67 (Hexane/EtOAc = 15/1); Mp: 92.4-93.8 °C; ^{1}H NMR (600 MHz, $CDCl_3$) δ 7.45-7.39 (m, 4H), 7.29-7.23 (m, 4H), 7.18-7.12 (m, 4H), 6.93 (t, J = 7.9 Hz, 1H), 6.46 (s, 1H), 5.50 (dd, J = dd, 9.3, 5.2 Hz, 1H), 3.27 (dd, J = 14.1, 9.3 Hz, 1H), 3.01 (dd, J = 14.1, 5.2 Hz, 1H); $^{13}C\{^1H\}$ NMR (151 MHz, $CDCl_3$) δ 149.9, 137.3, 133.2, 131.3, 130.9, 130.4, 129.7, 128.2, 128.1, 126.6, 126.6, 126.5, 124.2, 124.1, 122.7, 100.6, 79.1, 40.5; IR (neat): 3060, 3028, 2945, 2916, 1726, 1627, 1602, 1587, 1560, 1488, 1454, 1401, 1270, 1179, 1070, 1008, 795, 752 cm^{-1}; HRMS (APCI) m/z: $[M + H]^+$ Calcd for $C_{22}H_{18}BrO$ 377.0536; Found 377.0536.

1-Benzyl-3-(4-(trifluoromethyl)phenyl)-1H-isochromene (**3q**)

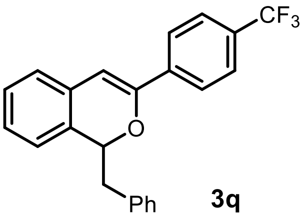

3q

63% yield; White solid; R_f = 0.49 (Hexane/EtOAc = 15/1) ; Mp:97.0-97.8 °C; ^{1}H NMR (600 MHz, $CDCl_3$) δ 7.65 (d, J = 8.2 Hz, 2H), 7.56 (d, J = 8.6 Hz, 2H), 7.30-7.25 (m, 4H), 7.19-7.14 (m, 4H), 6.95 (d, J = 7.6 Hz, 1H), 6.56 (s, 1H), 5.54 (dd, J = 9.3, 5.0 Hz, 1H), 3.28 (dd, J = 13.8, 9.3 Hz, 1H), 3.04 (dd, J = 13.8, 5.0 Hz, 1H); $^{13}C\{^1H\}$ NMR (151 MHz, $CDCl_3$) δ 149.5, 137.7, 137.3, 131.0, 130.1_2, 130.1_0 (q, $J_{C\text{-}F}$ = 32.6 Hz), 129.9, 129.7, 128.3, 128.2, 127.0, 126.5, 125.1, 124.4, 124.3, 124.1 (q, $J_{C\text{-}F}$ = 272.1 Hz), 102.1, 79.2, 40.6; IR (neat): 3068, 3029, 2946, 2915, 1616, 1487, 1455, 1414, 1325, 1167, 1125, 1112, 1069, 1014, 853, 795, 752 cm^{-1}; HRMS (APCI) m/z: $[M + H]^+$ Calcd for $C_{23}H_{18}F_3O$ 367.1304; Found 367.1304.

1-Benzyl-3-(o-tolyl)-1H-isochromene (**3r**)

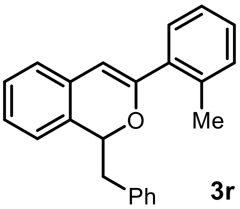

3r

73% yield; White solid; R_f = 0.40 (Hexane/EtOAc = 15/1); Mp: 83.0- 84.5 °C ; ^{1}H NMR (600 MHz, $CDCl_3$) δ 7.38 (d, J = 7.2 Hz, 1H), 7.26-7.22 (m, 5H), 7.18-7.13 (m, 4H), 7.09 (dd, J = 12.7, 7.6 Hz, 2H), 6.82 (d, J = 7.6 Hz, 1H), 6.01 (s, 1H), 5.49-5.44 (m, 1H), 3.36-3.31 (m, 2H), 2.28 (s, 3H); $^{13}C\{^1H\}$ NMR (151 MHz, $CDCl_3$) δ 153.9, 137.5, 136.7, 135.1, 131.2, 130.7, 129.9, 129.7, 128.9, 128.6, 128.2, 128.0, 126.4, 126.1, 125.5, 124.2, 123.6, 104.4, 79.0, 39.9, 20.8. IR (neat): 3063, 3029, 2959, 2871, 1729, 1630, 1604, 1542, 1488, 1455, 1375, 1270, 1119, 1063, 795, 762 cm^{-1}; HRMS (APCI) m/z: $[M + H]^+$ Calcd for $C_{23}H_{21}O$ 313.1587; Found 313.1587.

1-Methyl-3-phenyl-1-(4-(trifluoromethyl)benzyl)-1*H*-isochromene (**3s**)

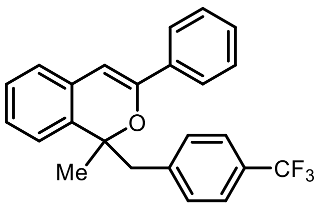

3s

50% yield; White foam; R_f = 0.66 (Hexane/EtOAc = 5/1); ^{1}H NMR (600 MHz, $CDCl_3$) δ 7.70-7.68 (m, 2H), 7.42-7.34 (m, 5H), 7.25-7.23 (m, 1H), 7.14-7.11 (m, 2H), 7.06 (d, *J* = 7.9 Hz, 2H), 6.93 (d, *J* = 7.2 Hz, 1H), 6.46 (s, 1H), 3.29 (d, *J* = 13.4 Hz, 1H), 3.16 (d, *J* = 13.4 Hz, 1H), 1.71 (s, 3H); ^{13}C{^{1}H} NMR (151 MHz, $CDCl_3$) δ 150.4, 140.7, 134.4, 134.1, 131.3, 130.6, 128.8, 128.6 (q, $J_{C\text{-}F}$ = 30.0 Hz), 128.3, 128.0, 126.5, 124.9, 124.5 (q, $J_{C\text{-}F}$ = 4.4 Hz), 124.3_4 (q, $J_{C\text{-}F}$ = 270.0 Hz), 124.3_3, 123.6, 100.2, 80.6, 44.8, 24.6; IR (neat): 3069, 3032, 2925, 2854, 1712, 1613, 1493, 1442, 1325, 1274, 1164, 1122, 1066, 1019, 849,793 cm^{-1}; HRMS (FD) *m*/*z*: $[M + H]^+$ Calcd for $C_{24}H_{20}F_3O$ 381.1461; Found 381.1461.

1-(4-Methoxybenzyl)-1-methyl-3-phenyl-1*H*-isochromene (**3t**)

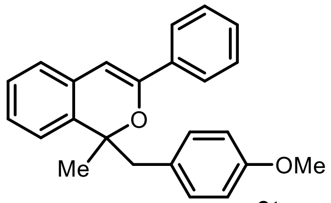

3t

78% yield; White foam; R_f = 0.60 (Hexane/EtOAc = 5/1); ^{1}H NMR (600 MHz, $CDCl_3$) δ 7.72 (d, *J* = 7.9 Hz, 2H), 7.39-7.34 (m, 3H), 7.22 (t, *J* = 7.4 Hz, 1H), 7.10 (t, *J* = 7.2 Hz, 2H), 6.88 (m, 3H), 6.71 (d, *J* = 8.2 Hz, 2H), 6.46 (s, 1H), 3.77 (s, 3H), 3.15 (d, *J* = 13.7 Hz, 1H), 3.06 (d, *J* = 13.7 Hz, 1H), 1.69 (s, 3H); ^{13}C{^{1}H} NMR (151 MHz, $CDCl_3$) δ 158.1, 150.5, 134.7, 134.6, 132.0, 130.6, 128.6, 128.6, 128.2, 127.6, 126.3, 125.0, 124.1, 123.8, 113.0, 100.0, 80.9, 55.2, 43.9, 24.4; IR (neat): 3064, 3032, 2953, 2834, 1730, 1716, 1699, 1685, 1651, 1631, 1558, 1541, 1508, 1457, 1247, 795, 669, 656 cm^{-1}; HRMS (FD) *m*/*z*: $[M + H]^+$ Calcd for $C_{24}H_{23}O$ 343.1693; Found 343.1693.

6. Catalytic cycles of the sequential transformation and theoretical studies

Catalytic cycles of the sequential transformation

As shown in Figure S3, the reported reaction is composed of three catalytic cycles, namely, catalytic cycles I and II and a photoredox cycle of the photocatalyst, 2-benzopyrylium cation. Catalytic cycle I generates 2-benzopyrylium cations **A** in situ from *ortho*-carbonyl alkynylbenzene derivatives **1** through the activation of alkyne moiety of **1** by π-Lewis acidic metal catalyst ([M]X: $AgNTf_2$ or $Cu(NTf_2)_2$) followed by the intramolecular cyclization and the proto-demetalation by trifluoroacetic acid (TFA). In catalytic cycle II, photo-excitation of generated **A** under light irradiation facilitates the single-electron transfer (SET) from benzyltrimethylsilane derivatives **2** as a donor molecule, resulting in the formation of radical cations **B**. Further desilylation from generated radical cations **B** affords nucleophilic arylmethyl radicals **C** which undergo addition reaction with 2-benzopyrylium intermediates **A**, giving radical cations **E**. Further reduction of radical cations **E** by SET from radical intermediates **D**, a reduced form of the photocatalyst, gives rise to corresponding 1*H*-isochromene derivatives **3**, which completes the photoredox cycle with the regeneration of cations **A**. The most distinctive feature of this sequential transformation is that the in situ-generated 2-benzopyrylium cations **A** are used not only as the electrophilic substrate but also as the photoredox catalyst.

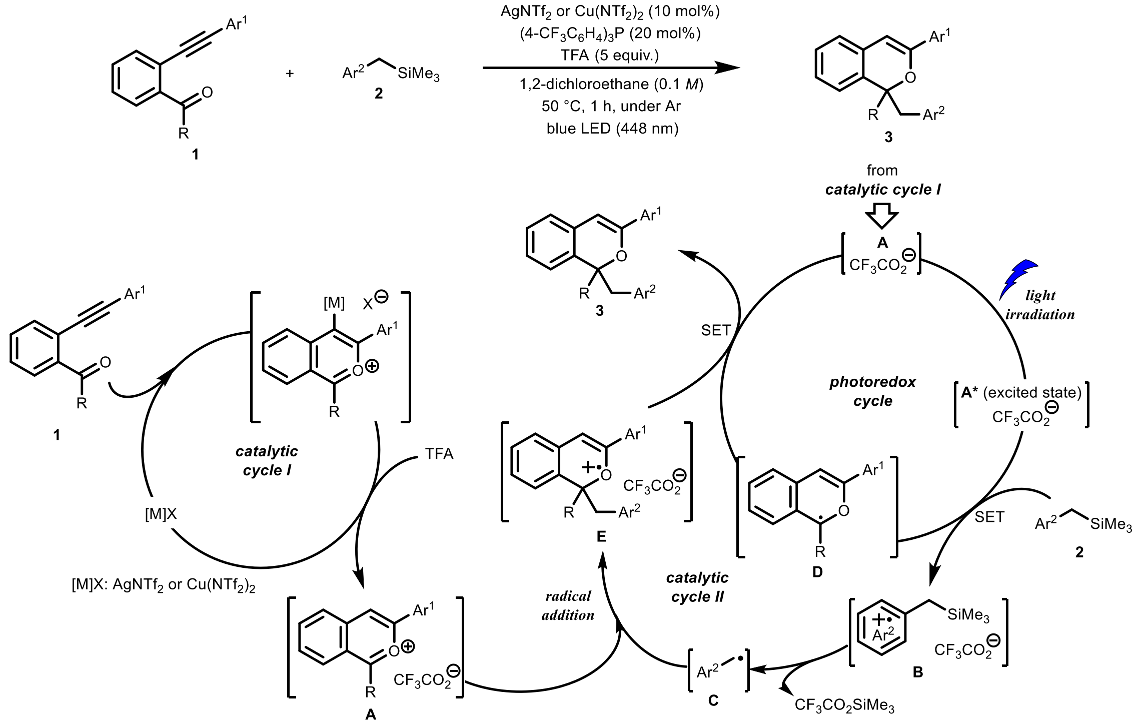

Figure S3. Plausible catalytic cycles

Theoretical studies of the sequential transformation

DFT calculation was conducted to determine the actual reaction pathway in the carbon–carbon bond-formation step. All calculations were performed with the Gaussian 16 package (Revision B.01). Geometries were optimized and characterized using frequency calculations at the UM06-2X/6-31G(d,p) level. Gibbs free energies in the solution phase were calculated using single-point energy calculations at the UM06-2X/6-311+G(d,p) level according to the SMD solvation model (dichloroethane: ε = 10.125) for the optimized structures.

First, we considered the radical addition pathway, of which mechanism was proposed in our previous paper.[10] As shown in Figure S4, the energy profile for the radical addition of benzyl radical **C** to 2-benzopyrylium intermediate **A** was identified. The radical addition pathway exhibits an energy barrier of **TS-1** ($\Delta G^{\neq}$ = +10.4 kcal/mol). On the other hand, as shown in Figure S5, the energy profile for the radical coupling of radical **D**, generated by a single electron reduction of 2-benzopyrylium, with benzyl radical **C** exhibited the energy barrier of **TS-2** ($\Delta G^{\neq}$ = +30.6 kcal/mol) which is much larger than that of **TS-1** ($\Delta G^{\neq}$ = +10.4 kcal/mol). These results strongly suggest that the radical addition (Figure S4) is plausible for the present reaction and the radical coupling (Figure S5) is unfavorable. In addition, the addition of benzyl radical **C** to 2-benzopyrylium intermediate **A** through **TS-1** generates benzylated radical cation **E** (Figure S4). As shown in Figure S6, SET from radical **D** to radical cation **E** gives product **3a** along with the generation of 2-benzopyrylium **A**. The process is exergonic by 37.8 kcal/mol and therefore thermodynamically favorable. From the above theoretical studies, we proposed a mechanism for the present radical addition pathway (Figures S3 and S4).

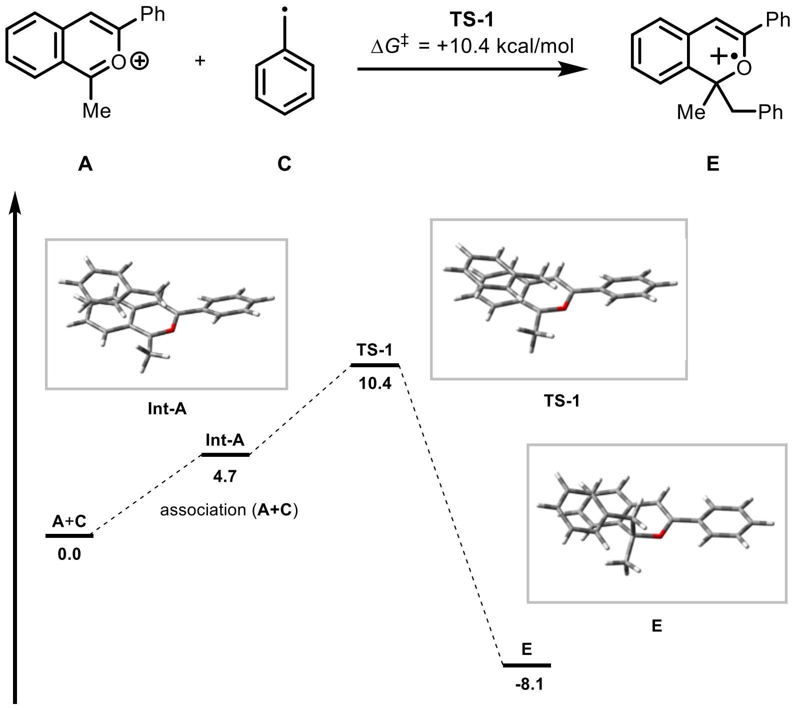

Figure S4. Radical addition pathway

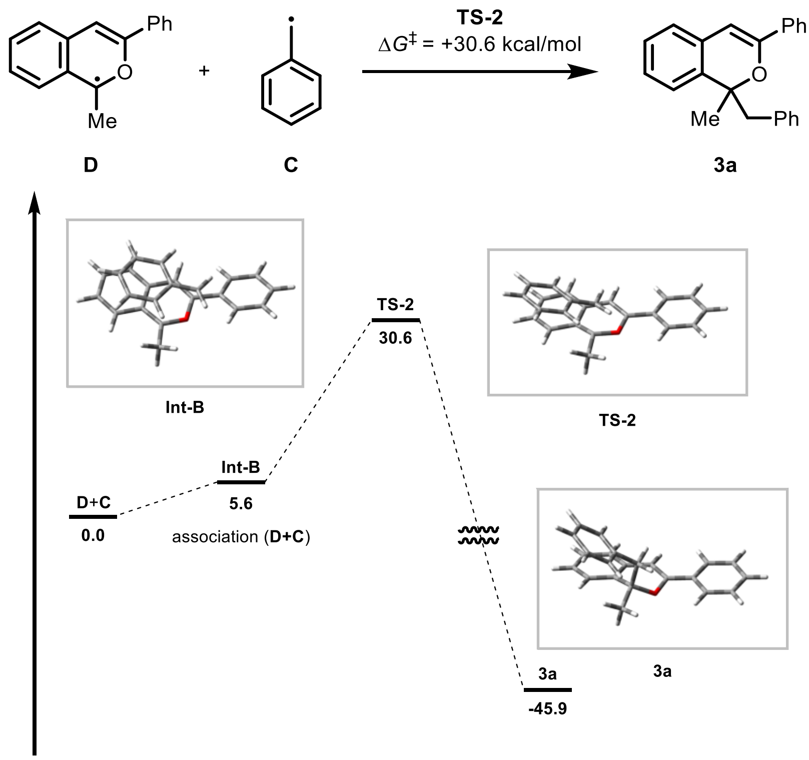

Figure S5. Radical coupling pathway

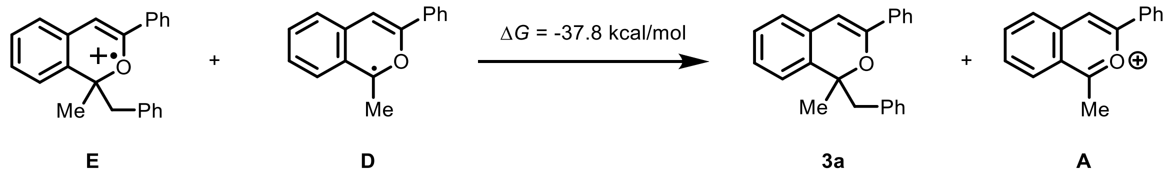

Figure S6. Electron transfer process

Cartesian coordinates

A
UM062X/6-31g(d,p)
E(UM062X) = -692.249135 hartree
Zero-point Energy Correction = 0.246496 hartree
Thermal Correction to Energy = 0.259762 hartree
Thermal correction to Enthalpy = 0.260706 hartree
Thermal correction to Gibbs Free Energy = 0.205908 hartree
Sum of electronic and Zero-point Energies = -692.002639 hartree
Sum of electronic and thermal Energies = -691.989373 hartree
Sum of electronic and thermal Enthalpies = -691.988429 hartree
Sum of electronic and thermal Free Energies= -692.043227 hartree
SMD(dichloroethane)/UM062X/6-311+g(d,p)
E(UM062X) = -692.482580 hartree
Gibbs Free Energy in dichloroethane = -692.276672 hartree
The number of Imaginary frequencies = 0

Center Number	Atomic Number	Atomic Type	Coordinates (Angstroms) X	Y	Z
1	6	0	0.275079	-1.152061	-0.096002
2	6	0	1.671383	-0.918174	-0.071426
3	6	0	-0.601271	-0.110987	-0.002292
4	6	0	2.620682	-1.961602	-0.161263
5	6	0	2.138855	0.427582	0.037051
6	6	0	3.961686	-1.667049	-0.136306
7	1	0	2.276817	-2.986668	-0.247302
8	6	0	3.535815	0.705292	0.059989
9	6	0	4.425954	-0.328888	-0.024380
10	1	0	3.883787	1.729000	0.143591
11	1	0	4.687192	-2.471067	-0.203468
12	1	0	5.492045	-0.134286	-0.007743
13	1	0	-0.105633	-2.159254	-0.212107
14	8	0	-0.097149	1.152760	0.092033
15	6	0	1.183441	1.445043	0.109624
16	6	0	-2.061247	-0.158393	-0.000889
17	6	0	-2.716275	-1.350899	0.336712
18	6	0	-2.809656	0.976060	-0.343027
19	6	0	-4.102407	-1.407736	0.320228
20	1	0	-2.148830	-2.224246	0.641501
21	6	0	-4.196067	0.909587	-0.358248
22	1	0	-2.308286	1.899951	-0.608801
23	6	0	-4.843334	-0.279860	-0.029350
24	1	0	-4.606086	-2.329040	0.590056
25	1	0	-4.772977	1.786168	-0.630622
26	1	0	-5.926834	-0.327136	-0.040792
27	6	0	1.464313	2.903135	0.213066
28	1	0	2.058860	3.109421	1.107425
29	1	0	2.035659	3.239509	-0.656793
30	1	0	0.529768	3.459277	0.267887

C
UM062X/6-31g(d,p)
E(UM062X) = -270.788424 hartree
Zero-point Energy Correction = 0.115638 hartree
Thermal Correction to Energy = 0.121302 hartree
Thermal correction to Enthalpy = 0.122246 hartree
Thermal correction to Gibbs Free Energy = 0.085981 hartree
Sum of electronic and Zero-point Energies = -270.672786 hartree
Sum of electronic and thermal Energies = -270.667122 hartree
Sum of electronic and thermal Enthalpies = -270.666178 hartree
Sum of electronic and thermal Free Energies= -270.702443 hartree
SMD(dichloroethane)/UM062X/6-311+g(d,p)
E(UM062X) = -270.862174 hartree
Gibbs Free Energy in dichloroethane = -270.776193 hartree
The number of Imaginary frequencies = 0

Center Number	Atomic Number	Atomic Type	Coordinates (Angstroms) X	Y	Z
1	6	0	1.834112	0.000000	-0.000010
2	6	0	1.131214	1.207826	-0.000005
3	6	0	-0.252233	1.213017	0.000003
4	6	0	-0.987558	0.000000	0.000005
5	6	0	-0.252233	-1.213017	0.000000
6	6	0	1.131214	-1.207826	-0.000008
7	1	0	2.918811	0.000000	-0.000016
8	1	0	1.672824	2.148491	-0.000006
9	1	0	-0.795455	2.153811	0.000007
10	1	0	-0.795455	-2.153811	0.000002
11	1	0	1.672824	-2.148491	-0.000012
12	6	0	-2.398224	0.000000	0.000013
13	1	0	-2.955650	0.928245	0.000017
14	1	0	-2.955650	-0.928245	0.000015

D
UM062X/6-31g(d,p)
E(UM062X) = -692.440925 hartree
Zero-point Energy Correction = 0.243583 hartree
Thermal Correction to Energy = 0.257277 hartree
Thermal correction to Enthalpy = 0.258221 hartree
Thermal correction to Gibbs Free Energy = 0.201341 hartree
Sum of electronic and Zero-point Energies = -692.197342 hartree
Sum of electronic and thermal Energies = -692.183648 hartree
Sum of electronic and thermal Enthalpies = -692.182704 hartree
Sum of electronic and thermal Free Energies= -692.239584 hartree
SMD(dichloroethane)/UM062X/6-311+g(d,p)
E(UM062X) = -692.622321 hartree
Gibbs Free Energy in dichloroethane = -692.420980 hartree
The number of Imaginary frequencies = 0

Center Number	Atomic Number	Atomic Type	Coordinates (Angstroms) X	Y	Z
1	6	0	0.257809	-1.120418	-0.079036
2	6	0	1.688747	-0.918772	-0.065115
3	6	0	-0.593197	-0.080531	0.016335
4	6	0	2.607768	-1.959056	-0.151710
5	6	0	2.144695	0.434165	0.033513
6	6	0	3.977899	-1.704335	-0.141186
7	1	0	2.240307	-2.978902	-0.227951
8	6	0	3.545938	0.673613	0.039080
9	6	0	4.433241	-0.382350	-0.045579
10	1	0	3.914837	1.691635	0.108165
11	1	0	4.686093	-2.522423	-0.207842
12	1	0	5.500219	-0.181914	-0.039549
13	1	0	-0.139793	-2.122109	-0.191973
14	8	0	-0.141987	1.207511	0.108420
15	6	0	1.207946	1.466383	0.113922
16	6	0	-2.064431	-0.157096	0.008463
17	6	0	-2.723653	-1.345523	0.346071
18	6	0	-2.824935	0.964778	-0.340156
19	6	0	-4.110004	-1.416119	0.310899
20	1	0	-2.149637	-2.210545	0.661821
21	6	0	-4.213124	0.890507	-0.370883
22	1	0	-2.319034	1.889702	-0.591414
23	6	0	-4.860748	-0.298941	-0.050008
24	1	0	-4.607081	-2.342680	0.579015
25	1	0	-4.790720	1.766394	-0.648138
26	1	0	-5.944135	-0.354376	-0.072427
27	6	0	1.518603	2.920246	0.214583
28	1	0	2.165276	3.133352	1.073146
29	1	0	2.032253	3.289740	-0.681054
30	1	0	0.591986	3.482741	0.335680

Int-A
UM062X/6-31g(d,p)
E(UM062X) = -963.058270 hartree
Zero-point Energy Correction = 0.363392 hartree
Thermal Correction to Energy = 0.383945 hartree
Thermal correction to Enthalpy = 0.384889 hartree
Thermal correction to Gibbs Free Energy = 0.312237 hartree
Sum of electronic and Zero-point Energies = -962.694878 hartree
Sum of electronic and thermal Energies = -962.674324 hartree
Sum of electronic and thermal Enthalpies = -962.673380 hartree
Sum of electronic and thermal Free Energies= -962.746033 hartree
SMD(dichloroethane)/UM062X/6-311+g(d,p)
E(UM062X) = -963.357675 hartree
Gibbs Free Energy in dichloroethane = -963.045438 hartree
The number of Imaginary frequencies = 0

Center Number	Atomic Number	Atomic Type	Coordinates (Angstroms) X	Y	Z
1	6	0	-1.014316	1.479834	-0.231631
2	6	0	0.335332	1.642572	0.177107
3	6	0	-1.770387	0.464439	0.270506
4	6	0	1.156883	2.688450	-0.295237
5	6	0	0.887491	0.692467	1.085042
6	6	0	2.464498	2.770037	0.122710
7	1	0	0.744705	3.419902	-0.982200
8	6	0	2.246511	0.788720	1.489357
9	6	0	3.016867	1.816237	1.013656
10	1	0	2.666271	0.051146	2.163938
11	1	0	3.091971	3.577709	-0.240157
12	1	0	4.055917	1.900123	1.310467
13	1	0	-1.448242	2.152953	-0.961073
14	8	0	-1.199467	-0.404007	1.152478
15	6	0	0.062298	-0.351433	1.520844
16	6	0	-3.166637	0.150626	-0.031817
17	6	0	-4.020869	1.156855	-0.501302
18	6	0	-3.648590	-1.154991	0.128628
19	6	0	-5.338339	0.855164	-0.816762
20	1	0	-3.667576	2.179214	-0.590369
21	6	0	-4.967840	-1.448402	-0.189861
22	1	0	-2.988775	-1.936824	0.489124
23	6	0	-5.812430	-0.446645	-0.663955
24	1	0	-5.999352	1.637985	-1.171303
25	1	0	-5.337258	-2.460852	-0.071261
26	1	0	-6.842890	-0.679135	-0.909834
27	6	0	0.469360	-1.438363	2.449195

28	1	0	1.383898	-1.909873	2.077074
29	1	0	0.678161	-1.027647	3.442167
30	1	0	-0.324142	-2.180199	2.529149
31	6	0	4.525197	-0.550855	-1.101858
32	6	0	3.499699	0.043854	-1.843783
33	6	0	2.208822	-0.449511	-1.772714
34	6	0	1.902818	-1.572191	-0.960111
35	6	0	2.960661	-2.162110	-0.218776
36	6	0	4.247634	-1.655278	-0.290823
37	1	0	5.537732	-0.167574	-1.168675
38	1	0	3.717892	0.892660	-2.484205
39	1	0	1.414263	0.007529	-2.357017
40	1	0	2.755255	-3.046378	0.380096
41	1	0	5.046687	-2.129986	0.269086
42	6	0	0.582973	-2.062400	-0.868305
43	1	0	-0.211746	-1.637428	-1.472418
44	1	0	0.357143	-2.953729	-0.293069

TS-1

UM062X/6-31g(d,p)
E(UM062X) = -963.054559 hartree
Zero-point Energy Correction = 0.364381 hartree
Thermal Correction to Energy = 0.383836 hartree
Thermal correction to Enthalpy = 0.384780 hartree
Thermal correction to Gibbs Free Energy = 0.314630 hartree
Sum of electronic and Zero-point Energies = -962.690179 hartree
Sum of electronic and thermal Energies = -962.670724 hartree
Sum of electronic and thermal Enthalpies = -962.669779 hartree
Sum of electronic and thermal Free Energies= -962.739929 hartree
SMD(dichloroethane)/UM062X/6-311+g(d,p)
E(UM062X) = -963.350958 hartree
Gibbs Free Energy in dichloroethane = -963.036328 hartree
The number of Imaginary frequencies = 1

Center Number	Atomic Number	Atomic Type	Coordinates (Angstroms) X	Y	Z
1	6	0	-1.306997	1.525091	-0.383439
2	6	0	0.024374	1.891389	-0.019985
3	6	0	-1.889484	0.414928	0.144253
4	6	0	0.631823	3.082824	-0.461475
5	6	0	0.759790	1.010859	0.818634
6	6	0	1.918453	3.386406	-0.067754
7	1	0	0.073206	3.758058	-1.101273
8	6	0	2.082431	1.330360	1.196170
9	6	0	2.645517	2.511319	0.765670
10	1	0	2.650168	0.651358	1.823126
11	1	0	2.379170	4.309795	-0.402148
12	1	0	3.656357	2.767774	1.061922
13	1	0	-1.870195	2.142827	-1.072127
14	8	0	-1.166649	-0.384973	0.978485
15	6	0	0.143141	-0.217501	1.194594
16	6	0	-3.257716	-0.061244	-0.071110
17	6	0	-4.250667	0.830667	-0.497079
18	6	0	-3.578711	-1.408546	0.137836
19	6	0	-5.541590	0.375243	-0.725495
20	1	0	-4.024658	1.884812	-0.620586
21	6	0	-4.872875	-1.856794	-0.091706
22	1	0	-2.814350	-2.101916	0.470960
23	6	0	-5.854166	-0.968322	-0.525784
24	1	0	-6.308114	1.071091	-1.047668
25	1	0	-5.116076	-2.901637	0.066082
26	1	0	-6.864457	-1.321115	-0.702464
27	6	0	0.666769	-1.126899	2.257838
28	1	0	1.714483	-1.374281	2.079589
29	1	0	0.585417	-0.628712	3.229969
30	1	0	0.075730	-2.042848	2.287346
31	6	0	4.990355	-1.298806	-0.771452
32	6	0	4.239869	-0.300643	-1.398558
33	6	0	2.856890	-0.353026	-1.367805
34	6	0	2.193001	-1.419854	-0.714772
35	6	0	2.972800	-2.419218	-0.081164
36	6	0	4.354470	-2.356236	-0.112089
37	1	0	6.073958	-1.256825	-0.800093
38	1	0	4.741208	0.513228	-1.910874
39	1	0	2.265196	0.422696	-1.848375
40	1	0	2.471750	-3.248718	0.411020
41	1	0	4.944761	-3.131102	0.364304
42	6	0	0.777818	-1.454156	-0.650796
43	1	0	0.191203	-0.801884	-1.293111
44	1	0	0.276675	-2.339849	-0.270669

E

UM062X/6-31g(d,p)
E(UM062X) = -963.088013 hartree
Zero-point Energy Correction = 0.368010 hartree
Thermal Correction to Energy = 0.387201 hartree
Thermal correction to Enthalpy = 0.388145 hartree
Thermal correction to Gibbs Free Energy = 0.318483 hartree
Sum of electronic and Zero-point Energies = -962.720003 hartree
Sum of electronic and thermal Energies = -962.700811 hartree
Sum of electronic and thermal Enthalpies = -962.699867 hartree
Sum of electronic and thermal Free Energies= -962.769530 hartree
SMD(dichloroethane)/UM062X/6-311+g(d,p)
E(UM062X) = -963.384267 hartree
Gibbs Free Energy in dichloroethane = -963.065784 hartree
The number of Imaginary frequencies = 0

Center Number	Atomic Number	Atomic Type	Coordinates (Angstroms) X	Y	Z
1	6	0	-1.369477	1.491367	-0.399080
2	6	0	-0.073735	1.912146	-0.041720
3	6	0	-1.883120	0.275670	0.084534
4	6	0	0.376467	3.224565	-0.338142
5	6	0	0.773132	1.010929	0.662791
6	6	0	1.625638	3.633235	0.073618
7	1	0	-0.282812	3.899180	-0.875314
8	6	0	2.026627	1.439509	1.066536
9	6	0	2.447029	2.742101	0.780708
10	1	0	2.700750	0.763970	1.581092
11	1	0	1.973871	4.636570	-0.142675
12	1	0	3.431510	3.062936	1.104128
13	1	0	-2.000554	2.131345	-1.001904
14	8	0	-1.121556	-0.536923	0.775188
15	6	0	0.337511	-0.423136	0.811991
16	6	0	-3.256520	-0.162581	-0.096601
17	6	0	-4.249267	0.727306	-0.548208
18	6	0	-3.596773	-1.497944	0.189185
19	6	0	-5.550282	0.283706	-0.719121
20	1	0	-4.018965	1.769235	-0.739540
21	6	0	-4.899142	-1.933426	0.010251
22	1	0	-2.830951	-2.181132	0.536869
23	6	0	-5.875581	-1.045334	-0.444456
24	1	0	-6.314810	0.972275	-1.060007
25	1	0	-5.157904	-2.964703	0.221485
26	1	0	-6.895078	-1.389462	-0.582836
27	6	0	0.722453	-1.044160	2.145472
28	1	0	1.805691	-1.150143	2.214027
29	1	0	0.367271	-0.427833	2.973348
30	1	0	0.276147	-2.038171	2.221777
31	6	0	5.107620	-1.388310	-0.796755
32	6	0	4.370063	-0.418171	-1.471603
33	6	0	2.984659	-0.387757	-1.348980
34	6	0	2.319490	-1.326171	-0.554214
35	6	0	3.068022	-2.303576	0.107865
36	6	0	4.454723	-2.333029	-0.008854
37	1	0	6.187552	-1.415656	-0.894576
38	1	0	4.873341	0.309222	-2.099710
39	1	0	2.410430	0.368991	-1.879369
40	1	0	2.560359	-3.055774	0.706825
41	1	0	5.023378	-3.100445	0.505239
42	6	0	0.822073	-1.272380	-0.402832
43	1	0	0.364423	-0.845831	-1.303160
44	1	0	0.409149	-2.276547	-0.261503

Int-B

UM062X/6-31g(d,p)
E(UM062X) = -963.243848 hartree
Zero-point Energy Correction = 0.360352 hartree
Thermal Correction to Energy = 0.381341 hartree
Thermal correction to Enthalpy = 0.382285 hartree
Thermal correction to Gibbs Free Energy = 0.307994 hartree
Sum of electronic and Zero-point Energies = -962.883496 hartree
Sum of electronic and thermal Energies = -962.862507 hartree
Sum of electronic and thermal Enthalpies = -962.861562 hartree
Sum of electronic and thermal Free Energies= -962.935854 hartree
SMD(dichloroethane)/UM062X/6-311+g(d,p)
E(UM062X) = -963.496209 hartree
Gibbs Free Energy in dichloroethane = -963.188215 hartree
The number of Imaginary frequencies = 0

Center Number	Atomic Number	Atomic Type	Coordinates (Angstroms) X	Y	Z
1	6	0	-0.536736	1.560770	-0.354911
2	6	0	0.863288	1.714590	-0.034258
3	6	0	-1.318268	0.707553	0.335544
4	6	0	1.704215	2.589378	-0.714614
5	6	0	1.383536	0.877063	1.004499
6	6	0	3.058374	2.670657	-0.397639
7	1	0	1.287697	3.208415	-1.505158
8	6	0	2.765374	0.987736	1.321979
9	6	0	3.574390	1.865380	0.627615
10	1	0	3.184410	0.376960	2.114119
11	1	0	3.705461	3.356770	-0.932656
12	1	0	4.627753	1.931705	0.882858
13	1	0	-0.959420	2.119788	-1.181811
14	8	0	-0.817646	-0.050687	1.355278
15	6	0	0.527069	-0.024616	1.640085
16	6	0	-2.749413	0.450007	0.099855
17	6	0	-3.541894	1.363472	-0.606405
18	6	0	-3.337239	-0.725861	0.581961

19	6	0	-4.882570	1.093750	-0.847698
20	1	0	-3.113407	2.300091	-0.947928
21	6	0	-4.681112	-0.990182	0.340766
22	1	0	-2.727479	-1.427965	1.139259
23	6	0	-5.457575	-0.085545	-0.377535
24	1	0	-5.484483	1.812661	-1.393953
25	1	0	-5.122819	-1.907635	0.716163
26	1	0	-6.506354	-0.292449	-0.563459
27	6	0	0.888364	-1.023850	2.681169
28	1	0	1.917278	-1.364935	2.547241
29	1	0	0.781634	-0.629520	3.699566
30	1	0	0.227951	-1.890839	2.585990
31	6	0	3.476663	-1.197290	-1.226902
32	6	0	2.462044	-0.614935	-1.988330
33	6	0	1.148739	-1.026687	-1.837032
34	6	0	0.802672	-2.038464	-0.906738
35	6	0	1.853159	-2.626564	-0.154971
36	6	0	3.162041	-2.207484	-0.311905
37	1	0	4.501564	-0.858915	-1.335741
38	1	0	2.700219	0.179310	-2.688589
39	1	0	0.357610	-0.559768	-2.417361
40	1	0	1.610784	-3.410279	0.558454
41	1	0	3.949061	-2.665754	0.279616
42	6	0	-0.540923	-2.432823	-0.722815
43	1	0	-1.340577	-1.978209	-1.295101
44	1	0	-0.800926	-3.210277	-0.014043

TS-2
UM062X/6-31g(d,p)
E(UM062X) = -963.205091 hartree
Zero-point Energy Correction = 0.360291 hartree
Thermal Correction to Energy = 0.380122 hartree
Thermal correction to Enthalpy = 0.381066 hartree
Thermal correction to Gibbs Free Energy = 0.309865 hartree
Sum of electronic and Zero-point Energies = -962.844800 hartree
Sum of electronic and thermal Energies = -962.824970 hartree
Sum of electronic and thermal Enthalpies = -962.824025 hartree
Sum of electronic and thermal Free Energies= -962.895226 hartree
SMD(dichloroethane)/UM062X/6-311+g(d,p)
E(UM062X) = -963.458304 hartree
Gibbs Free Energy in dichloroethane = -963.148439 hartree
The number of Imaginary frequencies = 1

Center Number	Atomic Number	Atomic Type	Coordinates (Angstroms) X	Y	Z
1	6	0	-1.249773	1.573223	-0.340784
2	6	0	0.038550	1.913929	0.006686
3	6	0	-1.879385	0.422433	0.181566
4	6	0	0.703859	3.101787	-0.463038
5	6	0	0.780627	1.020109	0.889142
6	6	0	1.966487	3.396791	-0.061641
7	1	0	0.163177	3.762773	-1.134862
8	6	0	2.108105	1.365017	1.274181
9	6	0	2.682425	2.522707	0.835652
10	1	0	2.663754	0.697327	1.925458
11	1	0	2.450880	4.301511	-0.414597
12	1	0	3.687921	2.784639	1.145085
13	1	0	-1.808175	2.206376	-1.020714
14	8	0	-1.145021	-0.400218	1.009210
15	6	0	0.197017	-0.216089	1.189585
16	6	0	-3.217523	-0.032032	-0.062589
17	6	0	-4.133885	0.738835	-0.815569
18	6	0	-3.667878	-1.271352	0.448736
19	6	0	-5.421030	0.285313	-1.048839
20	1	0	-3.837475	1.704291	-1.211330
21	6	0	-4.959762	-1.711195	0.207939
22	1	0	-2.988101	-1.879442	1.033652
23	6	0	-5.850035	-0.943177	-0.542675
24	1	0	-6.102210	0.899498	-1.629911
25	1	0	-5.277800	-2.668019	0.611133
26	1	0	-6.860014	-1.292278	-0.727413
27	6	0	0.728698	-1.154043	2.233361
28	1	0	1.784148	-1.371094	2.057792
29	1	0	0.618417	-0.711635	3.228295
30	1	0	0.166011	-2.088720	2.204846
31	6	0	4.922660	-1.428570	-0.857263
32	6	0	4.208682	-0.333957	-1.343972
33	6	0	2.822824	-0.318771	-1.280116
34	6	0	2.115260	-1.409424	-0.731410
35	6	0	2.855774	-2.503514	-0.233907
36	6	0	4.240217	-2.512763	-0.300247
37	1	0	6.006470	-1.437864	-0.908549
38	1	0	4.736535	0.514555	-1.767286
39	1	0	2.265245	0.543408	-1.639068
40	1	0	2.322182	-3.348572	0.194053
41	1	0	4.794113	-3.365741	0.078524
42	6	0	0.689574	-1.360459	-0.611203
43	1	0	0.131011	-0.660001	-1.227216
44	1	0	0.155141	-2.258048	-0.313142

3a
UM062X/6-31g(d,p)
E(UM062X) = -963.340849 hartree
Zero-point Energy Correction = 0.368054 hartree
Thermal Correction to Energy = 0.386997 hartree
Thermal correction to Enthalpy = 0.387942 hartree
Thermal correction to Gibbs Free Energy = 0.319823 hartree
Sum of electronic and Zero-point Energies = -962.972796 hartree
Sum of electronic and thermal Energies = -962.953852 hartree
Sum of electronic and thermal Enthalpies = -962.952908 hartree
Sum of electronic and thermal Free Energies= -963.021027 hartree
SMD(dichloroethane)/UM062X/6-311+g(d,p)
E(UM062X) = -963.590130 hartree
Gibbs Free Energy in dichloroethane = -963.270307 hartree
The number of Imaginary frequencies = 0

Center Number	Atomic Number	Atomic Type	Coordinates (Angstroms) X	Y	Z
1	6	0	-1.282739	1.459615	-0.531918
2	6	0	0.037057	1.934190	-0.142461
3	6	0	-1.825165	0.406355	0.108779
4	6	0	0.522820	3.189838	-0.521422
5	6	0	0.824482	1.110246	0.681685
6	6	0	1.765657	3.628420	-0.078942
7	1	0	-0.088413	3.824246	-1.157336
8	6	0	2.059739	1.563961	1.132485
9	6	0	2.532383	2.820203	0.756191
10	1	0	2.677402	0.926043	1.756722
11	1	0	2.133474	4.603860	-0.380885
12	1	0	3.502993	3.157860	1.103681
13	1	0	-1.847226	1.990449	-1.288518
14	8	0	-1.122818	-0.294714	1.039463
15	6	0	0.317076	-0.298121	0.934396
16	6	0	-3.196183	-0.103592	-0.081942
17	6	0	-4.208783	0.715816	-0.595091
18	6	0	-3.497496	-1.428811	0.249802
19	6	0	-5.486860	0.211442	-0.797516
20	1	0	-3.998311	1.758055	-0.812219
21	6	0	-4.779527	-1.929418	0.050468
22	1	0	-2.715570	-2.056819	0.661425
23	6	0	-5.776468	-1.113847	-0.477435
24	1	0	-6.263718	0.857718	-1.193074
25	1	0	-5.000174	-2.960543	0.307207
26	1	0	-6.777053	-1.504742	-0.631084
27	6	0	0.785707	-0.886424	2.255661
28	1	0	1.861639	-1.069517	2.242919
29	1	0	0.543910	-0.208616	3.077048
30	1	0	0.275251	-1.838697	2.422083
31	6	0	4.915594	-1.645660	-0.907145
32	6	0	4.222153	-0.576655	-1.469742
33	6	0	2.851348	-0.449148	-1.269395
34	6	0	2.150841	-1.385142	-0.501750
35	6	0	2.857682	-2.455222	0.053450
36	6	0	4.229373	-2.586985	-0.145074
37	1	0	5.984380	-1.746950	-1.066057
38	1	0	4.749453	0.159831	-2.067713
39	1	0	2.313846	0.390657	-1.703241
40	1	0	2.322385	-3.194676	0.644557
41	1	0	4.760251	-3.427089	0.291444
42	6	0	0.672466	-1.227978	-0.257308
43	1	0	0.185868	-0.820023	-1.149720
44	1	0	0.215214	-2.201836	-0.047584

7. References

1 N. Chernyak, S. I. Gorelsky, V. Gevorgyan, *Angew. Chem. Int. Ed.* **2011**, *50*, 2342–2345.

2 K. Saito, Y. Kajiwara, T. Akiyama, *Angew. Chem. Int. Ed*. **2013**, *52*, 13284–13288.

3 J. Sedelmeier, S. V. Ley, H. Lange, I. R. Baxendale. *Eur. J. Org. Chem.* **2009**, 4412–4420.

4 M. -E. Domaradzki, Y. Long, Z. She, X. Liu, G. Zhang, Y. Chen, *J. Org. Chem.* **2015**, *80*, 11360–11368.

5 M. Terada, F. Li, Y, Toda, *Angew. Chem. Int. Ed.* **2014**, *53*, 235–239.

6 N. Asao, K. Sato, *Org. Lett.* 2006, **8**, 5361–5363.

7 S. Bhunia, K.-C. Wang, R.-S. Liu, *Angew. Chem. Int. Ed.* **2008**, *47*, 5063–5066.

8 P. -C. Too, S. Chiba, *Chem. Commun.*, **2012**, *48*, 7634–7636.

9 N. Nardangeli, N. Topolovčan, R. Simionescu, T. Hudlický, *Eur. J. Org. Chem.* **2020**, 227–233.

10 M. Terada, R. Yazaki, R. Obayashi, Z. Iwasaki, S. Umemiya, J. Kikuchi, *Chem. Sci.* **2024**, *15*, 6115–6121.
