# Supplementary material for: Development of a flow photochemical process for a π-Lewis acidic metal-catalyzed cyclization/radical addition sequence: in situ-generated 2-benzopyrylium as photoredox catalyst and reactive intermediate
Source: Beilstein J Org Chem. 2024 Aug 13;20:1973–80. doi: 10.3762/bjoc.20.173 (PMC11331546; doi:10.3762/bjoc.20.173)

BEILSTEIN JOURNAL OF ORGANIC CHEMISTRY

Supporting Information

for

Development of a flow photochemical process for a π-Lewis acidic metal-catalyzed cyclization/radical addition sequence: in situ-generated 2-benzopyrylium as photoredox catalyst and reactive intermediate

Masahiro Terada, Zen Iwasaki, Ryohei Yazaki, Shigenobu Umemiya and Jun Kikuchi

Beilstein J. Org. Chem. **2024,** *20,* 1973–1980. doi:10.3762/bjoc.20.173

Copies of NMR spectra

License and Terms: This is a supporting information file under the terms of the Creative Commons Attribution License (https://creativecommons.org/licenses/by/4.0). Please note that the reuse, redistribution and reproduction in particular requires that the author(s) and source are credited and that individual graphics may be subject to special legal provisions.
The license is subject to the *Beilstein Journal of Organic Chemistry* terms and conditions: (https://www.beilstein-journals.org/bjoc/terms)

NMR Spectra

^{1}H NMR (600 MHz, $CDCl_3$) and $^{13}C\{^{1}H\}$ NMR (151 MHz, $CDCl_3$) spectra of **1a**.

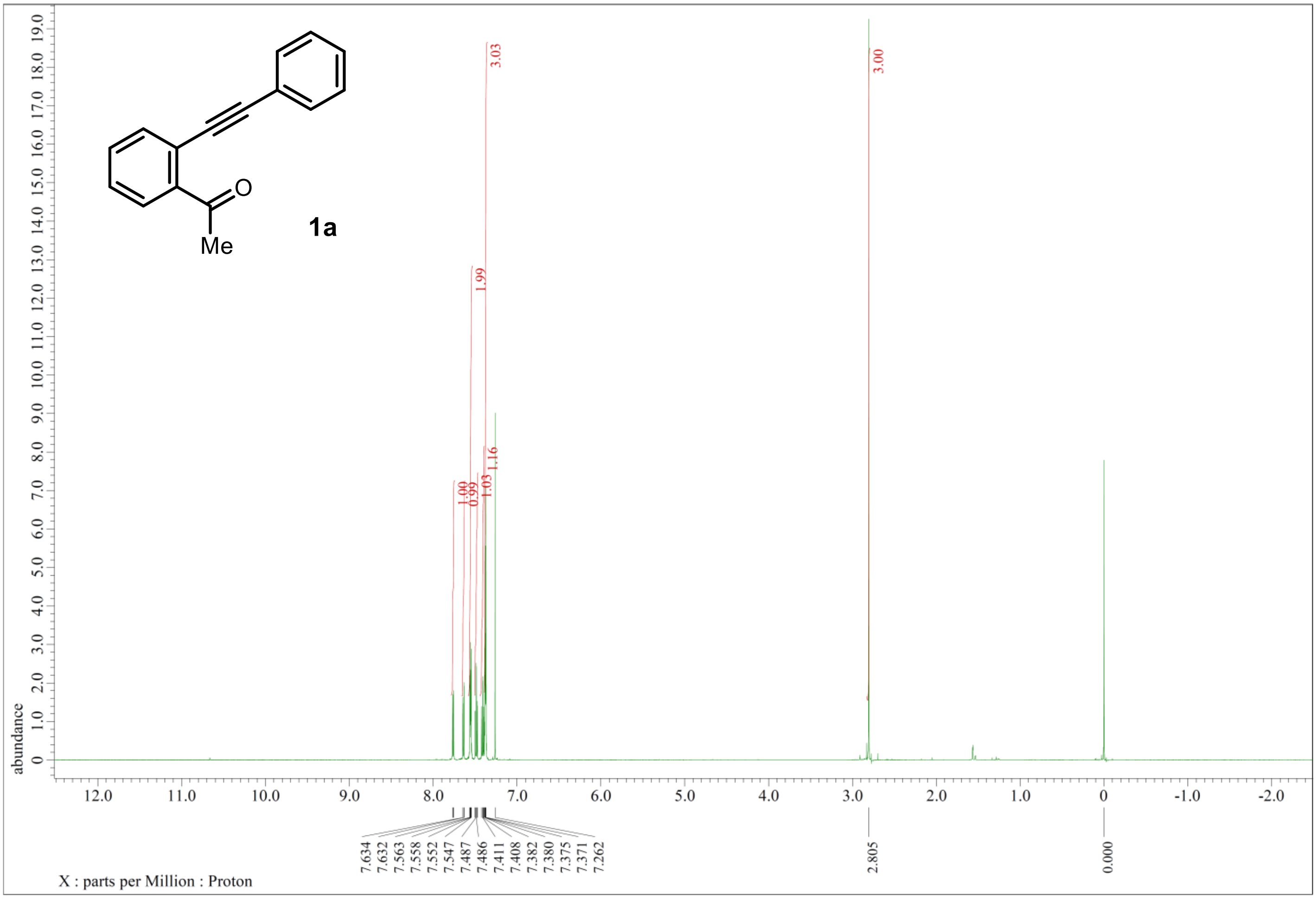

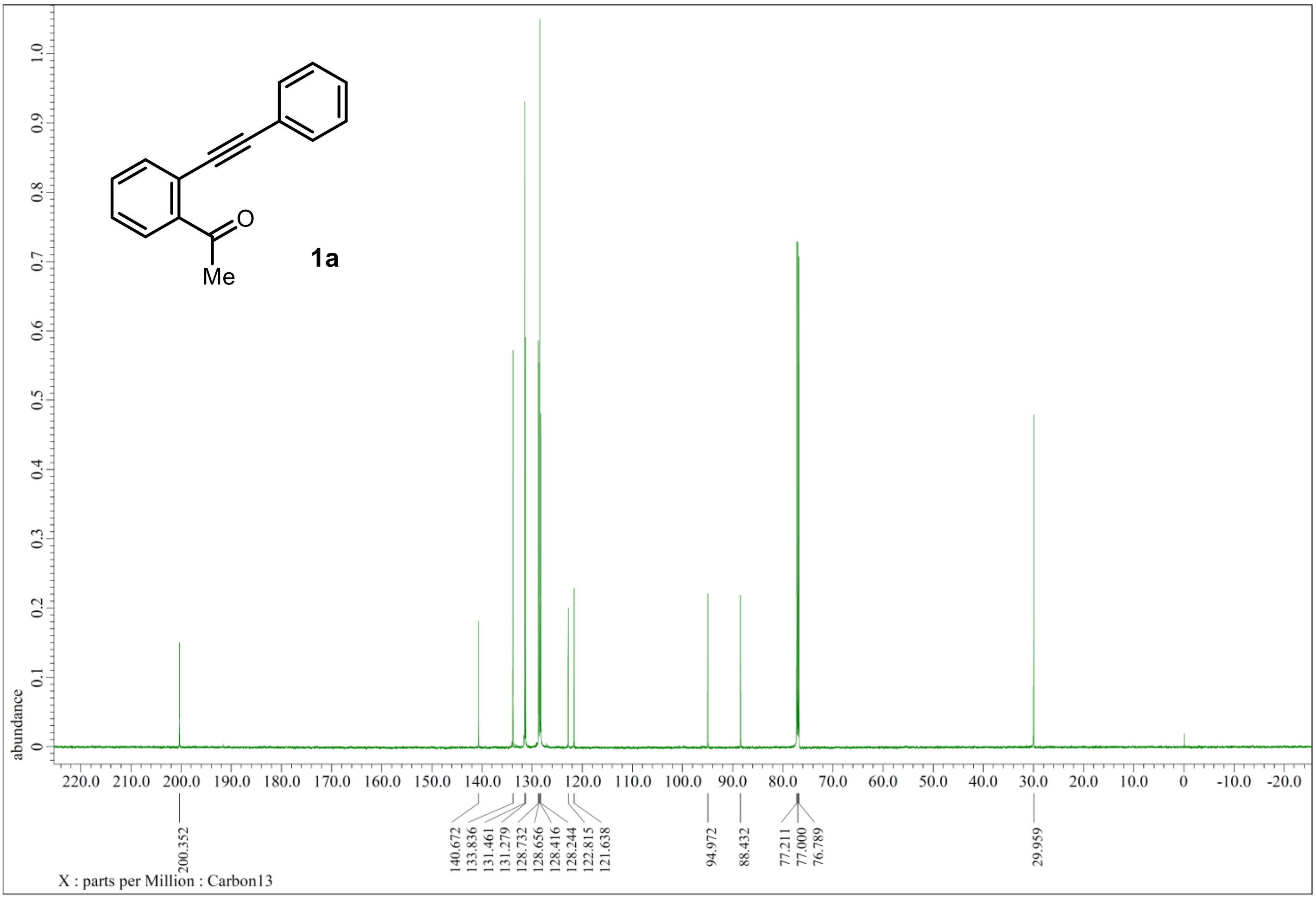

^{1}H NMR (600 MHz, $CDCl_3$) and $^{13}C\{^{1}H\}$ NMR (151 MHz, $CDCl_3$) spectra of **1b**.

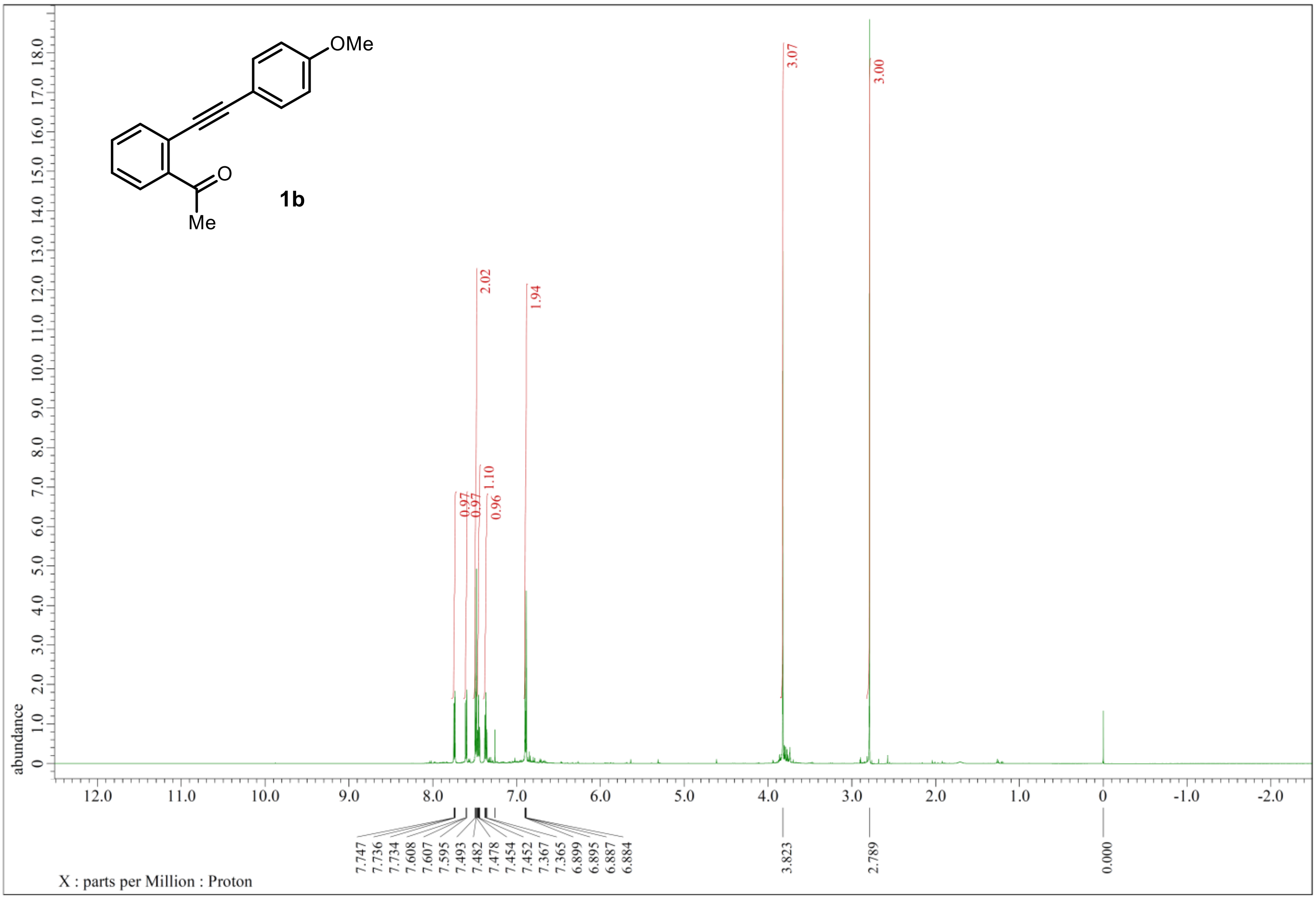

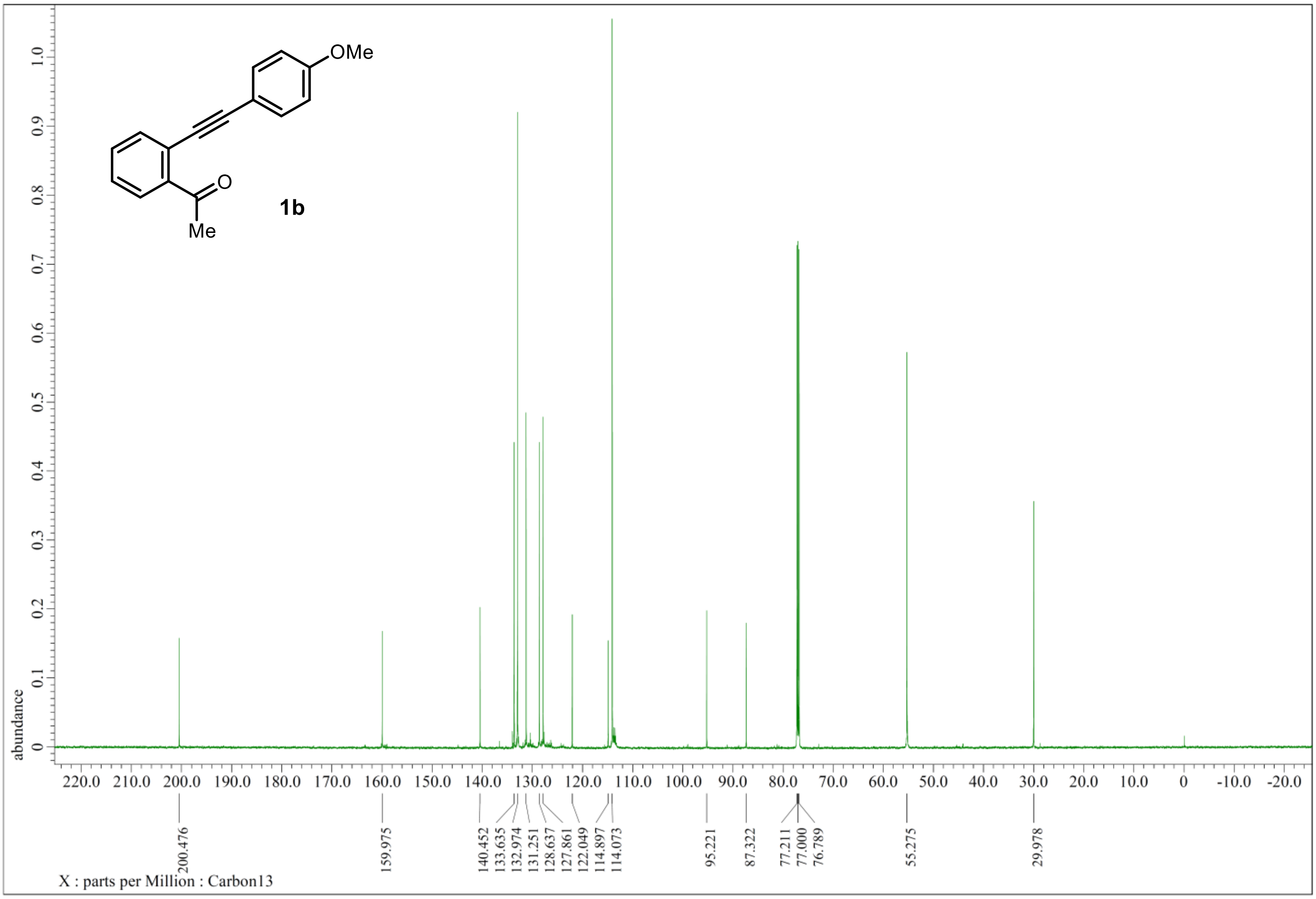

^{1}H NMR (600 MHz, $CDCl_3$) and $^{13}C\{^{1}H\}$ NMR (151 MHz, $CDCl_3$) spectra of **1c**.

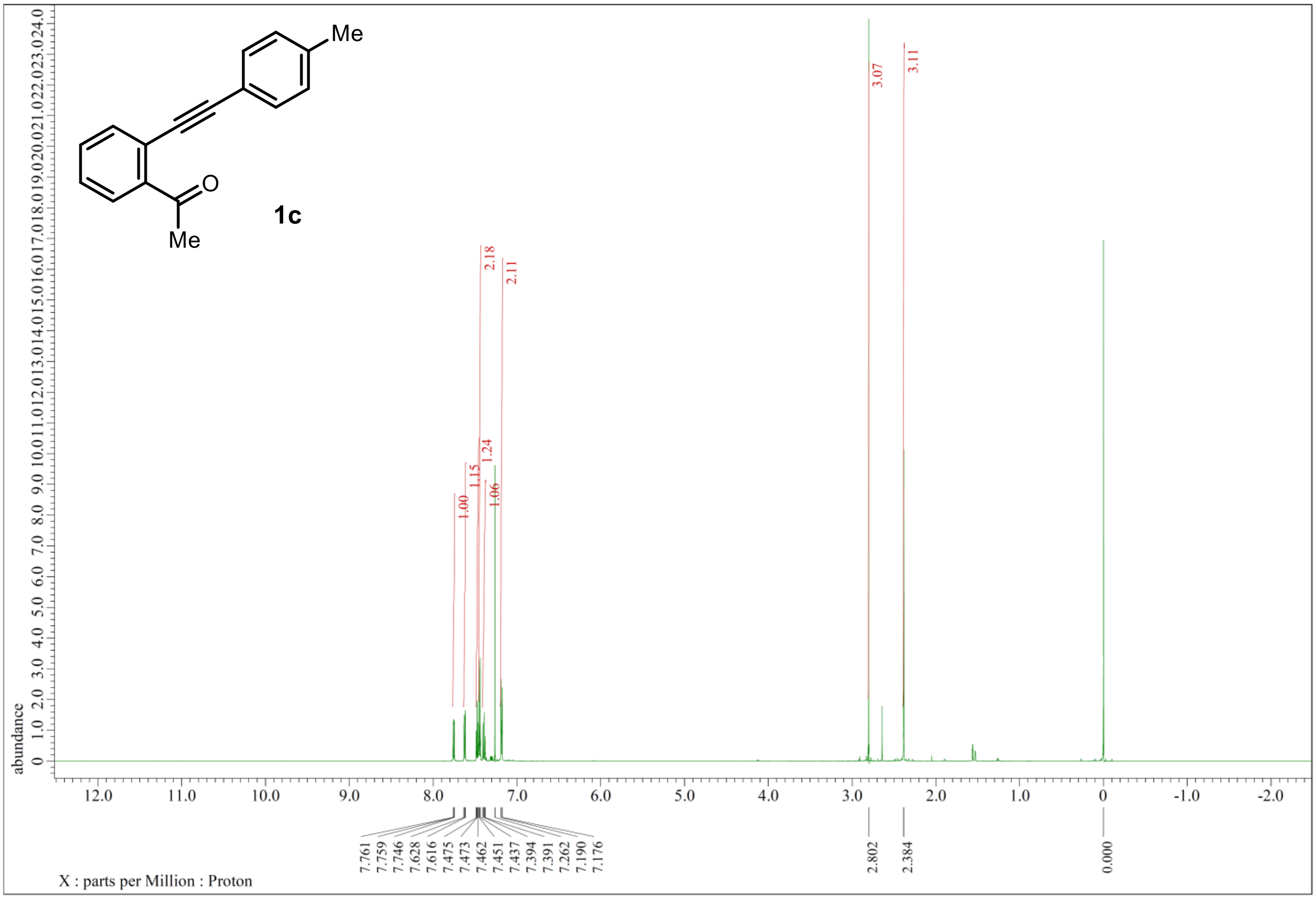

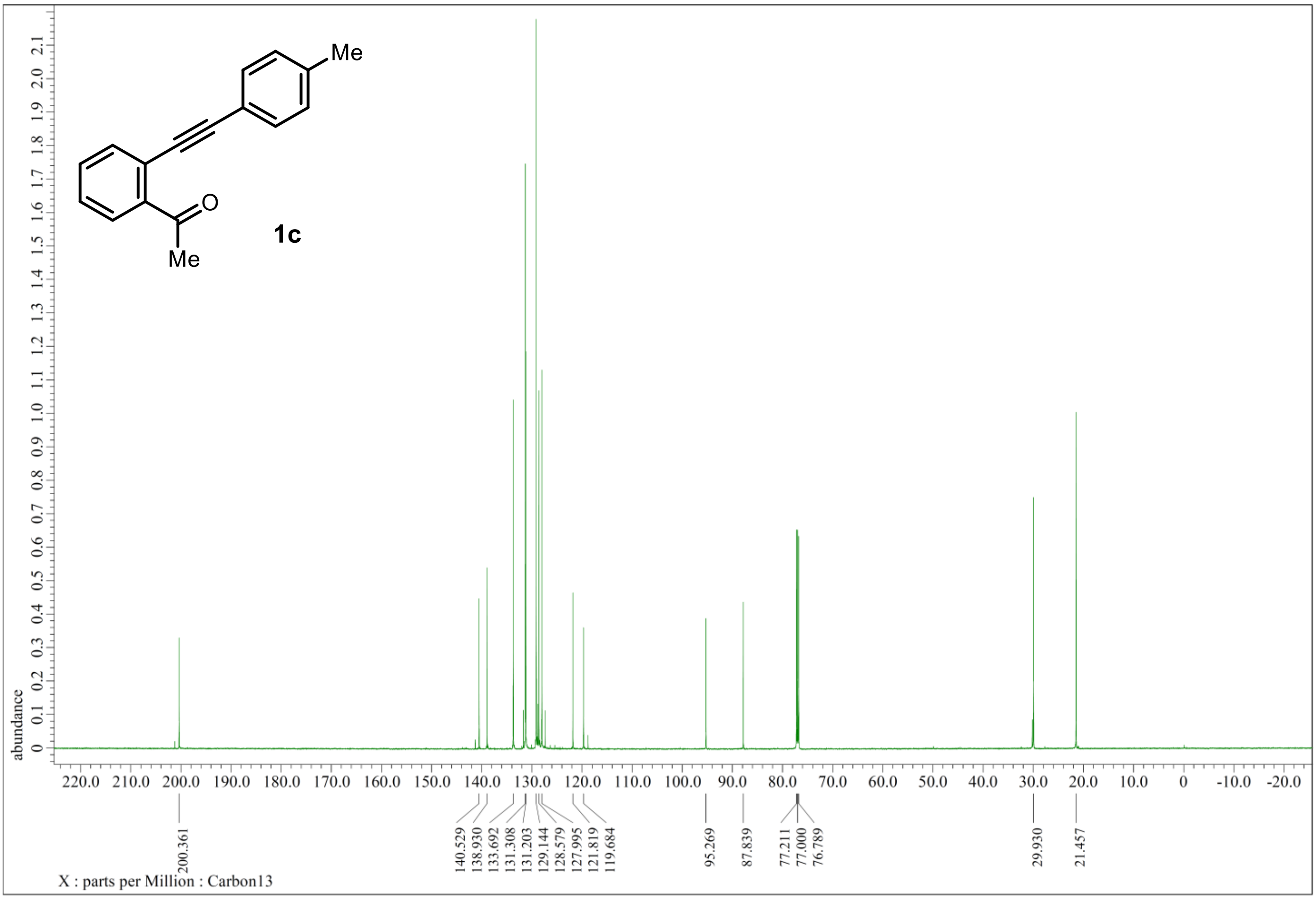

^{1}H NMR (600 MHz, $CDCl_3$) and ^{13}C{^{1}H} NMR (151 MHz, $CDCl_3$) spectra of **1d**.

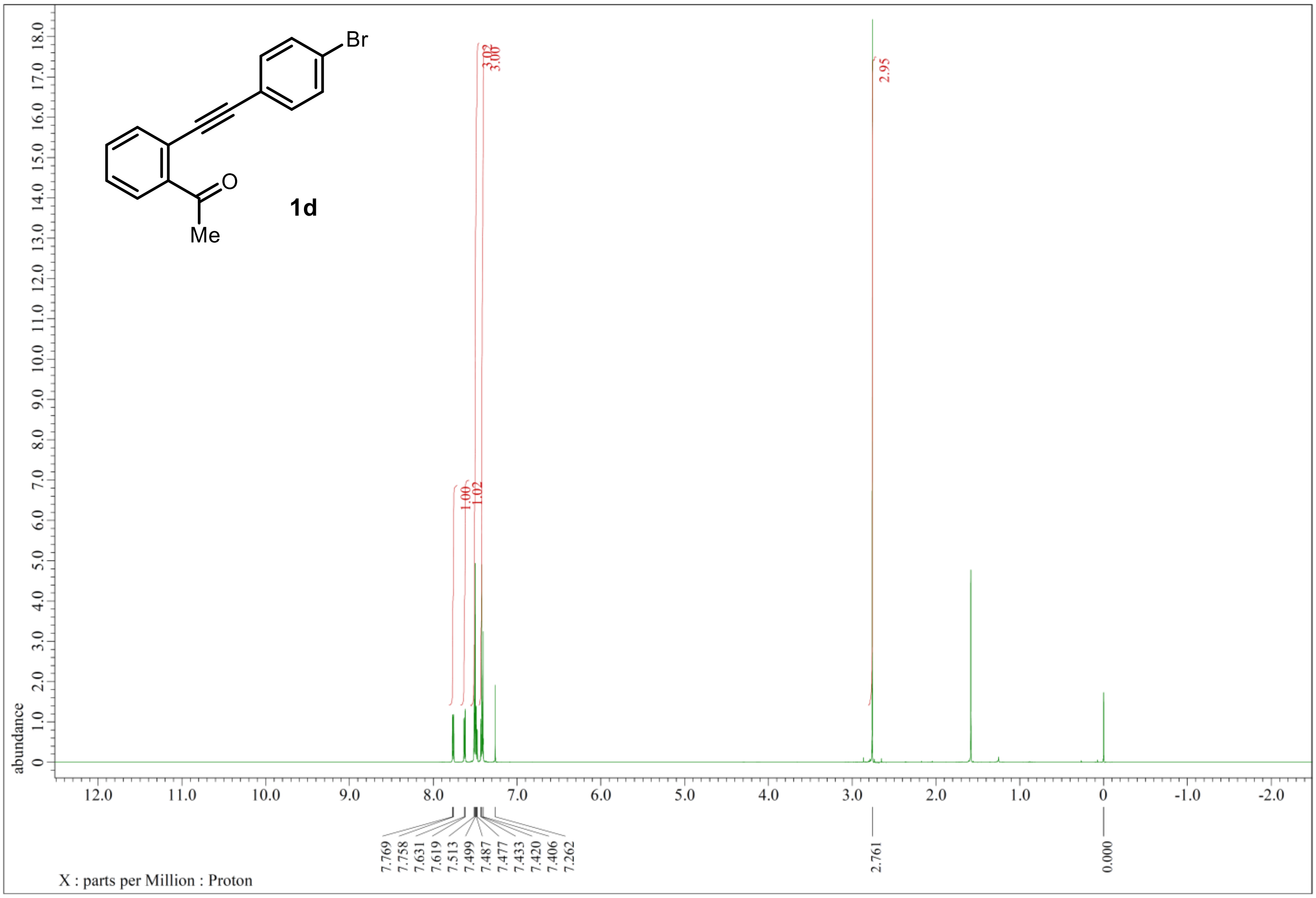

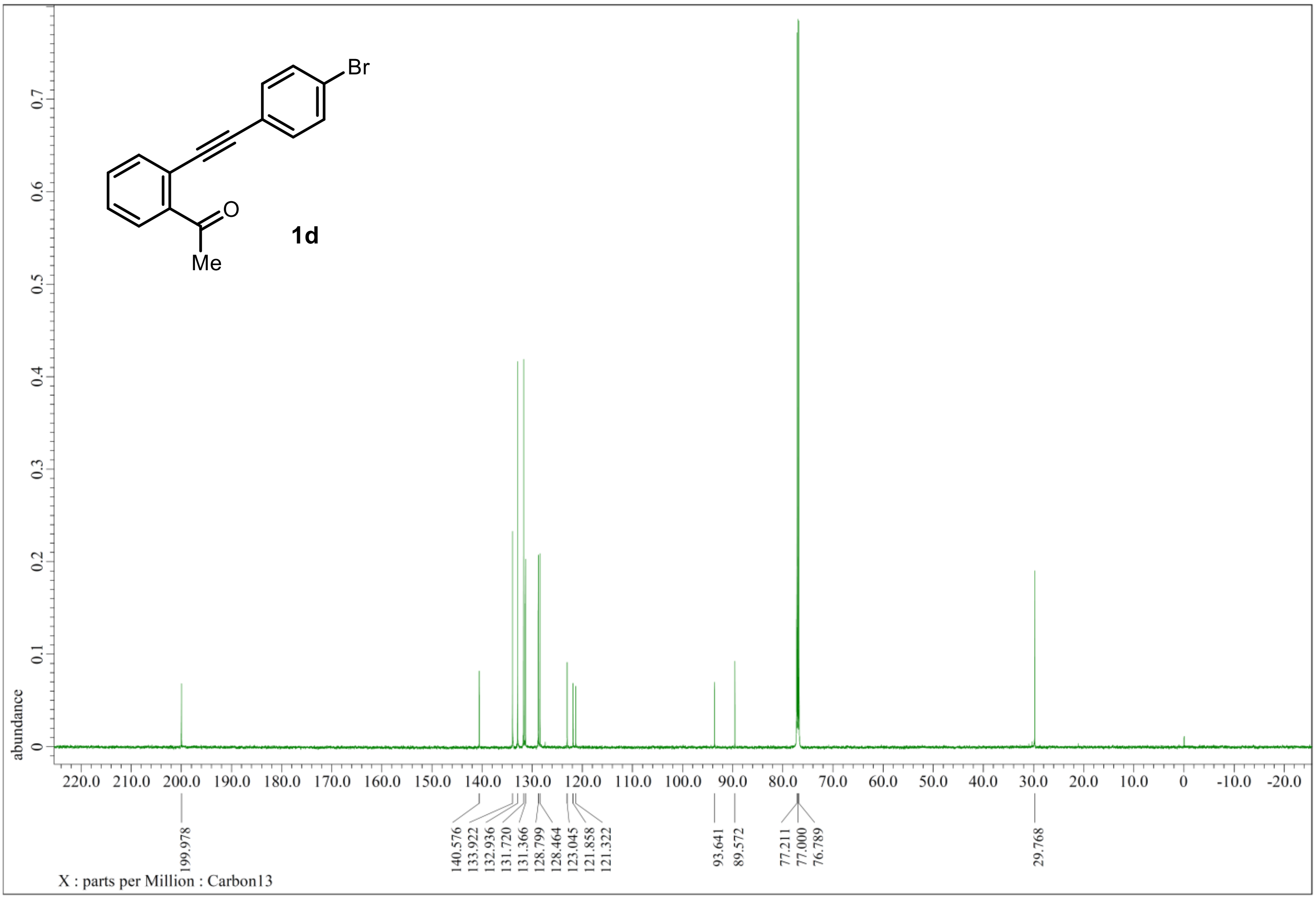

^{1}H NMR (600 MHz, $CDCl_3$) and $^{13}C\{^{1}H\}$ NMR (151 MHz, $CDCl_3$) spectra of **1e**.

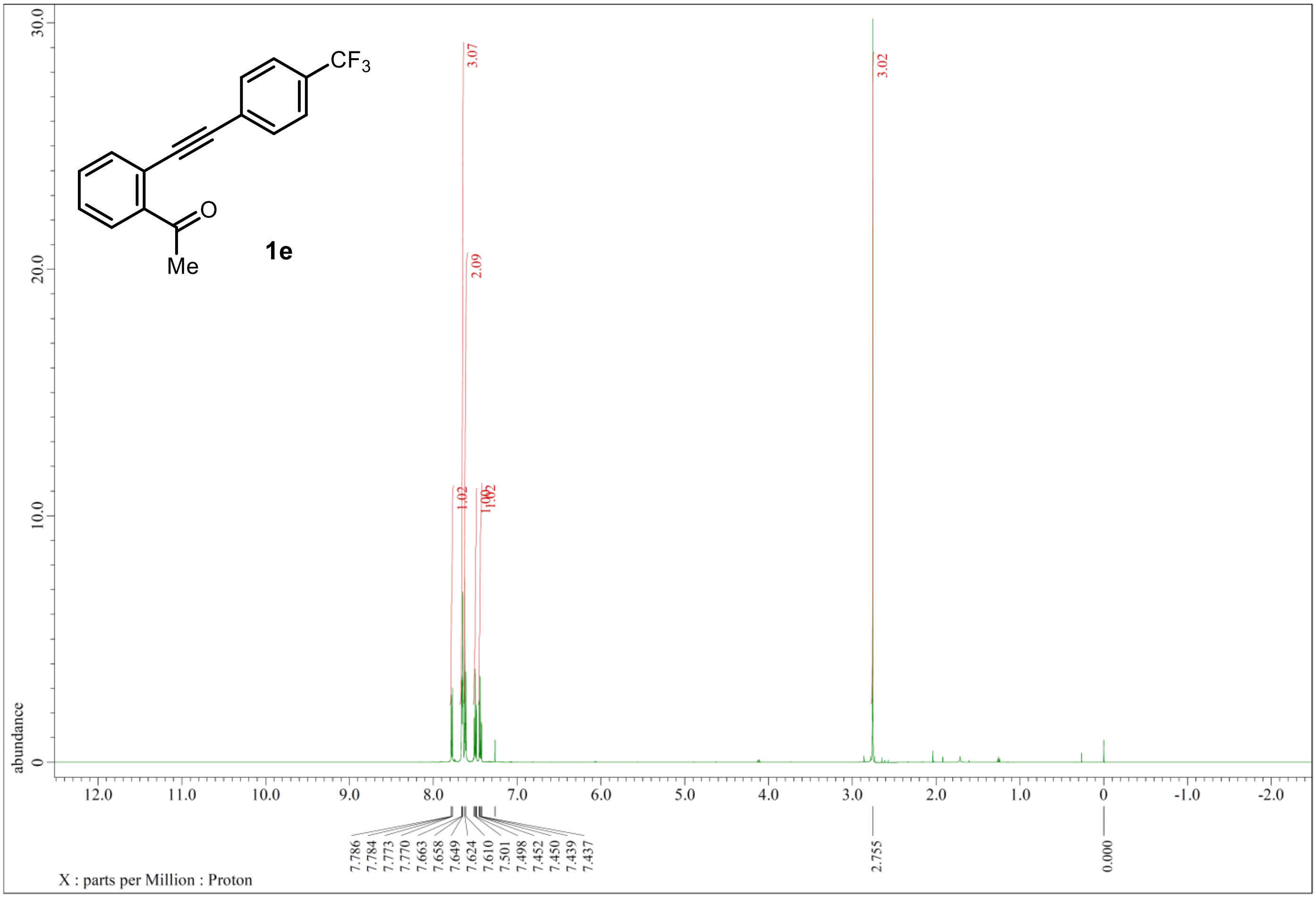

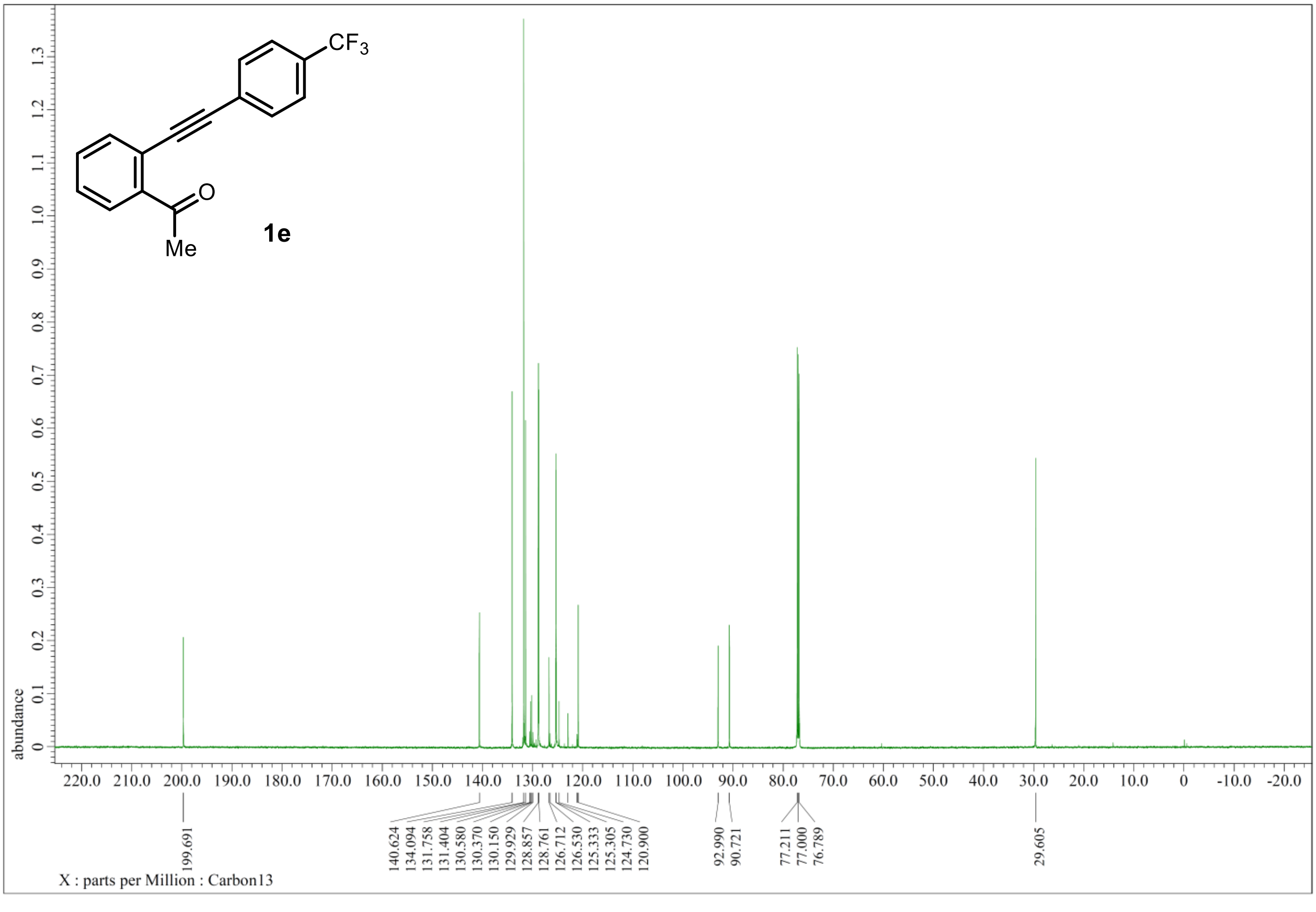

^{1}H NMR (600 MHz, $CDCl_3$) and $^{13}C\{^{1}H\}$ NMR (151 MHz, $CDCl_3$) spectra of **1f**.

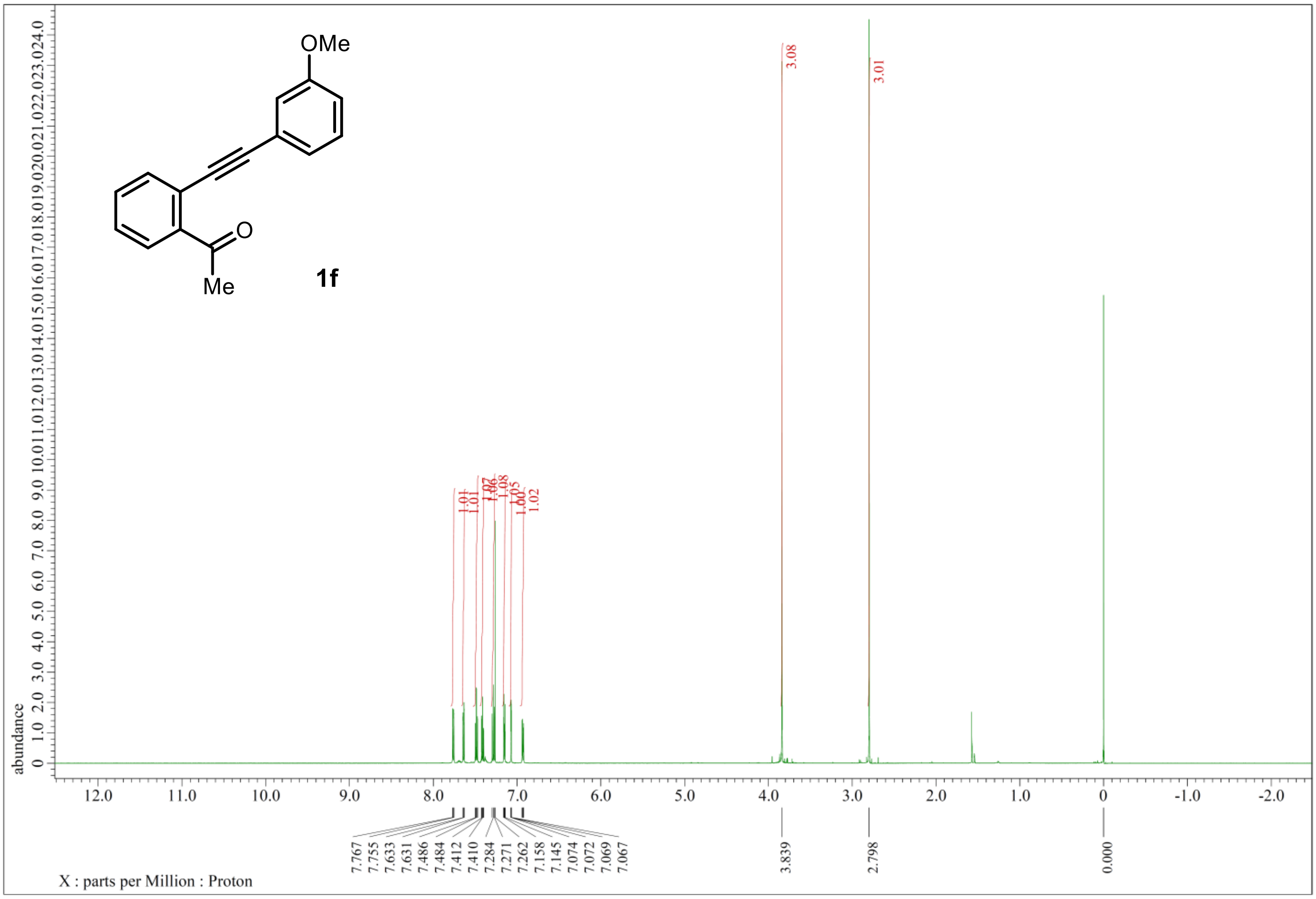

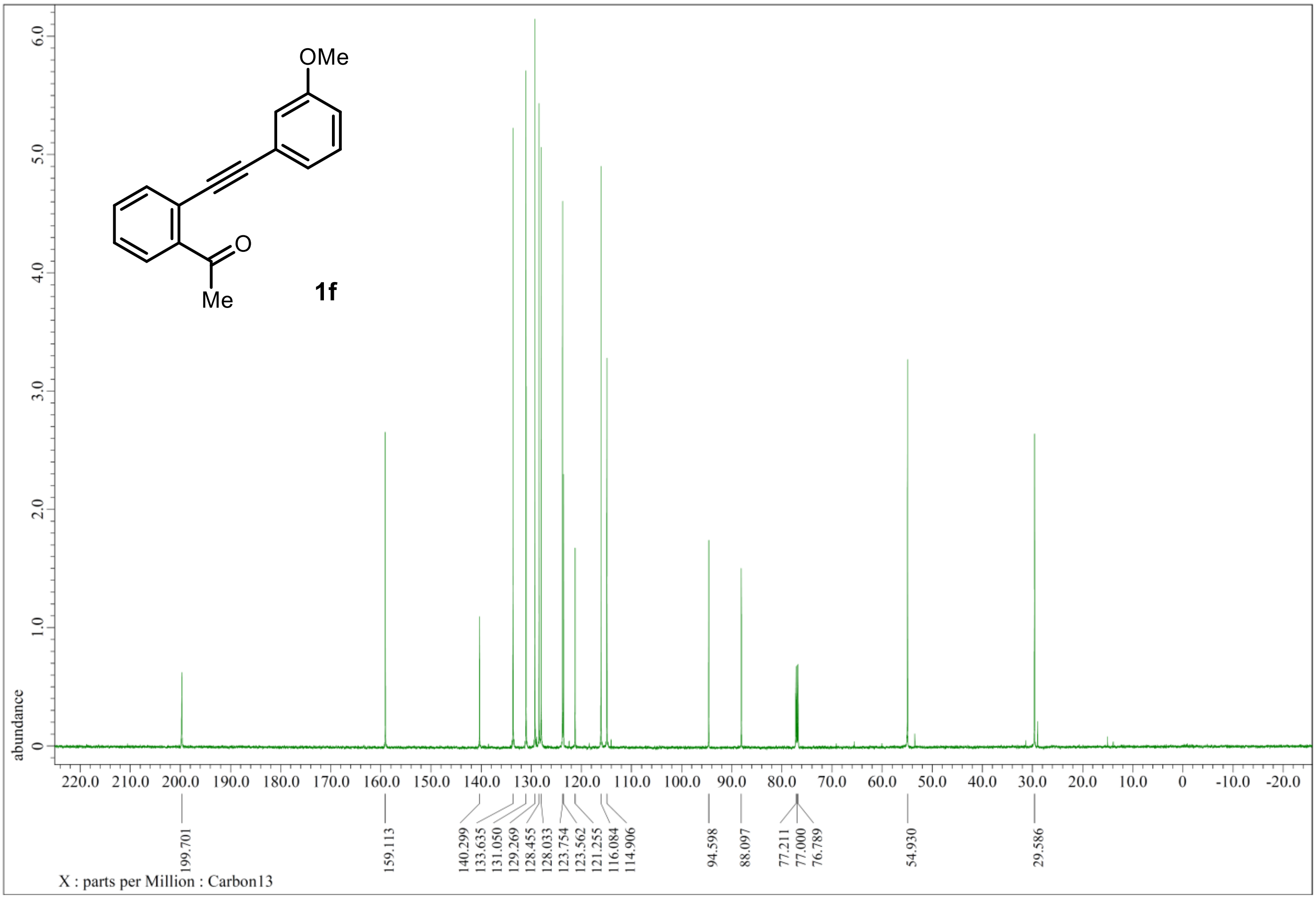

^{1}H NMR (600 MHz, $CDCl_3$) and $^{13}C\{^{1}H\}$ NMR (151 MHz, $CDCl_3$) spectra of **1g**.

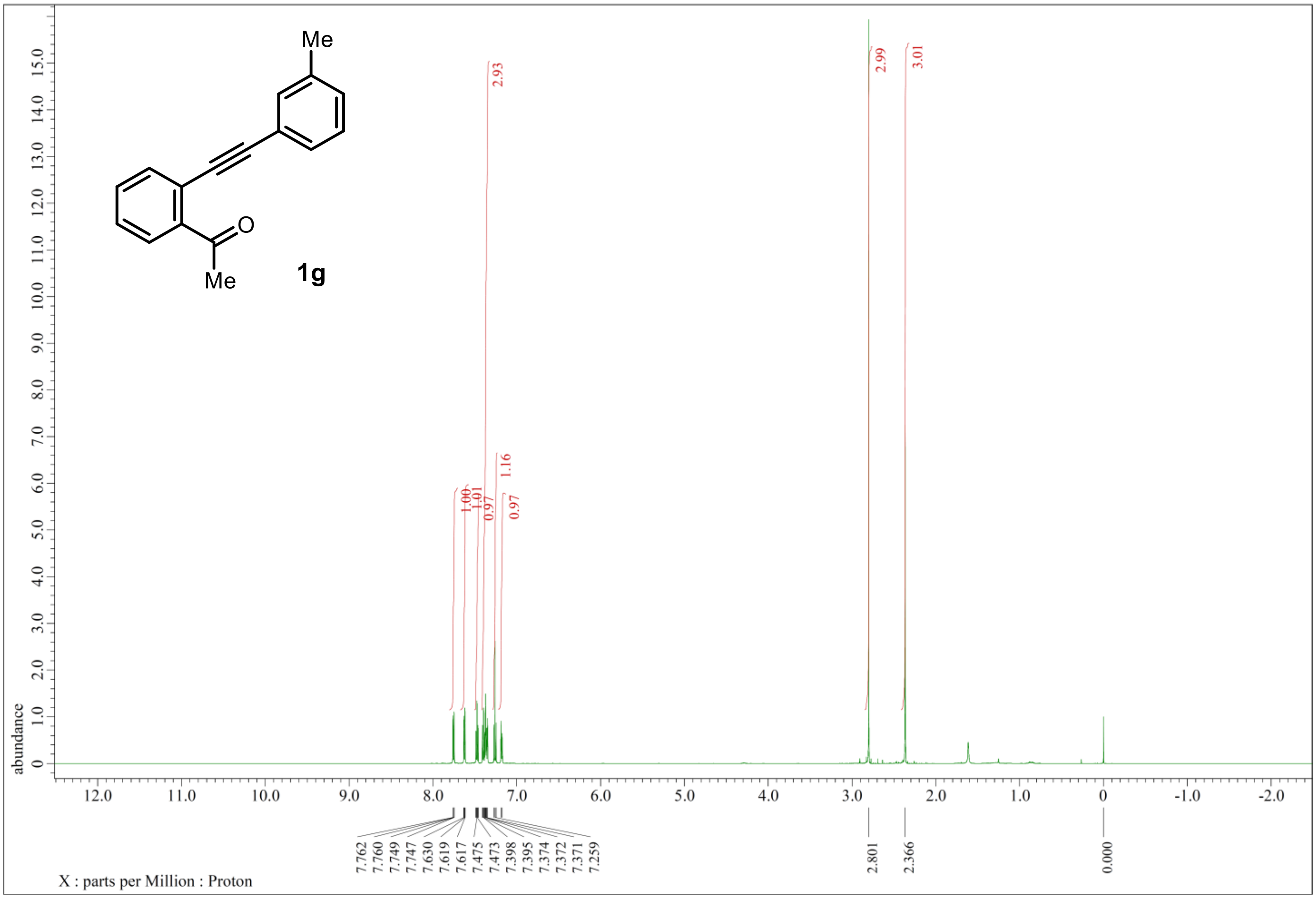

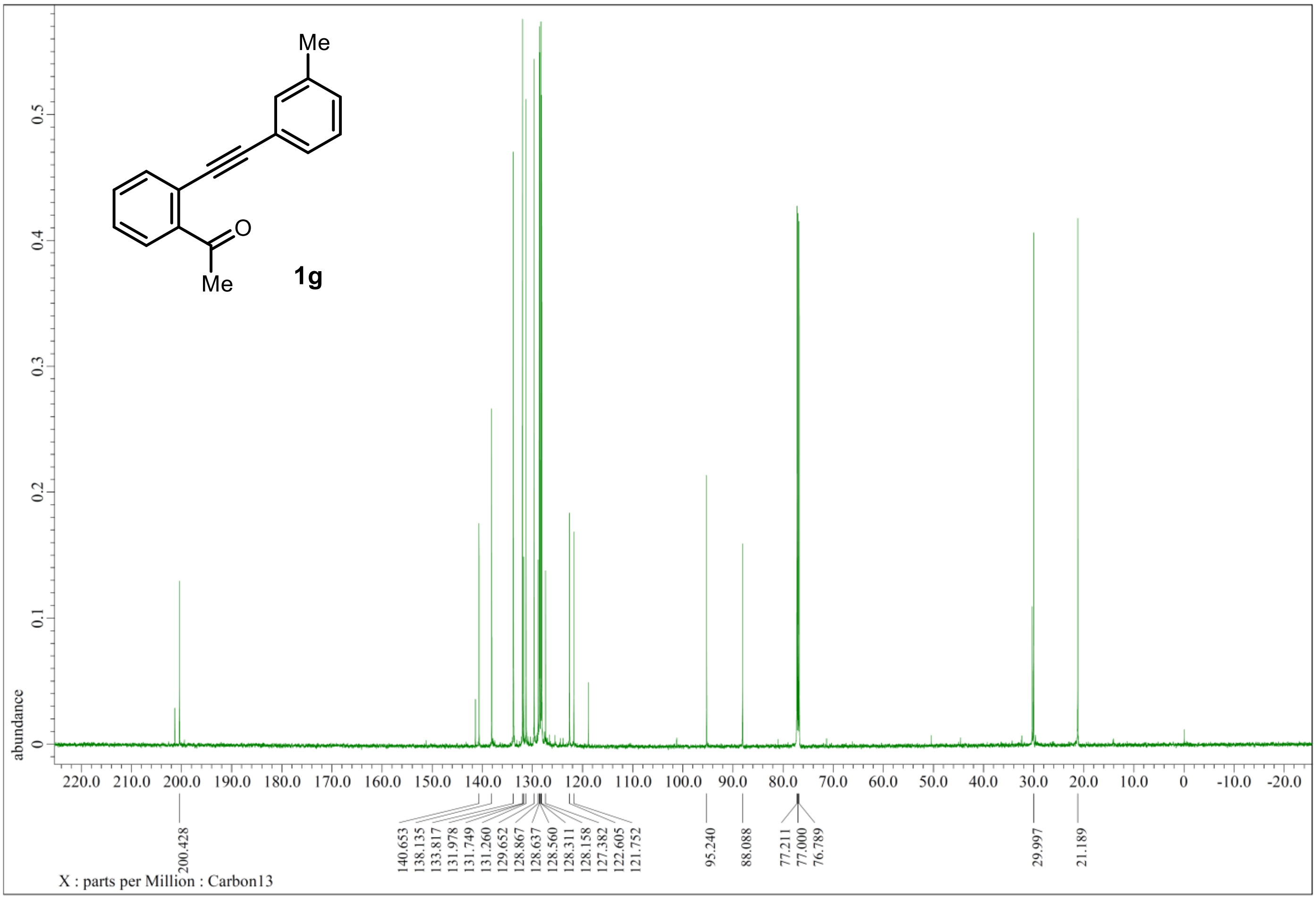

^{1}H NMR (600 MHz, $CDCl_3$) and ^{13}C{^{1}H} NMR (151 MHz, $CDCl_3$) spectra of **1h**.

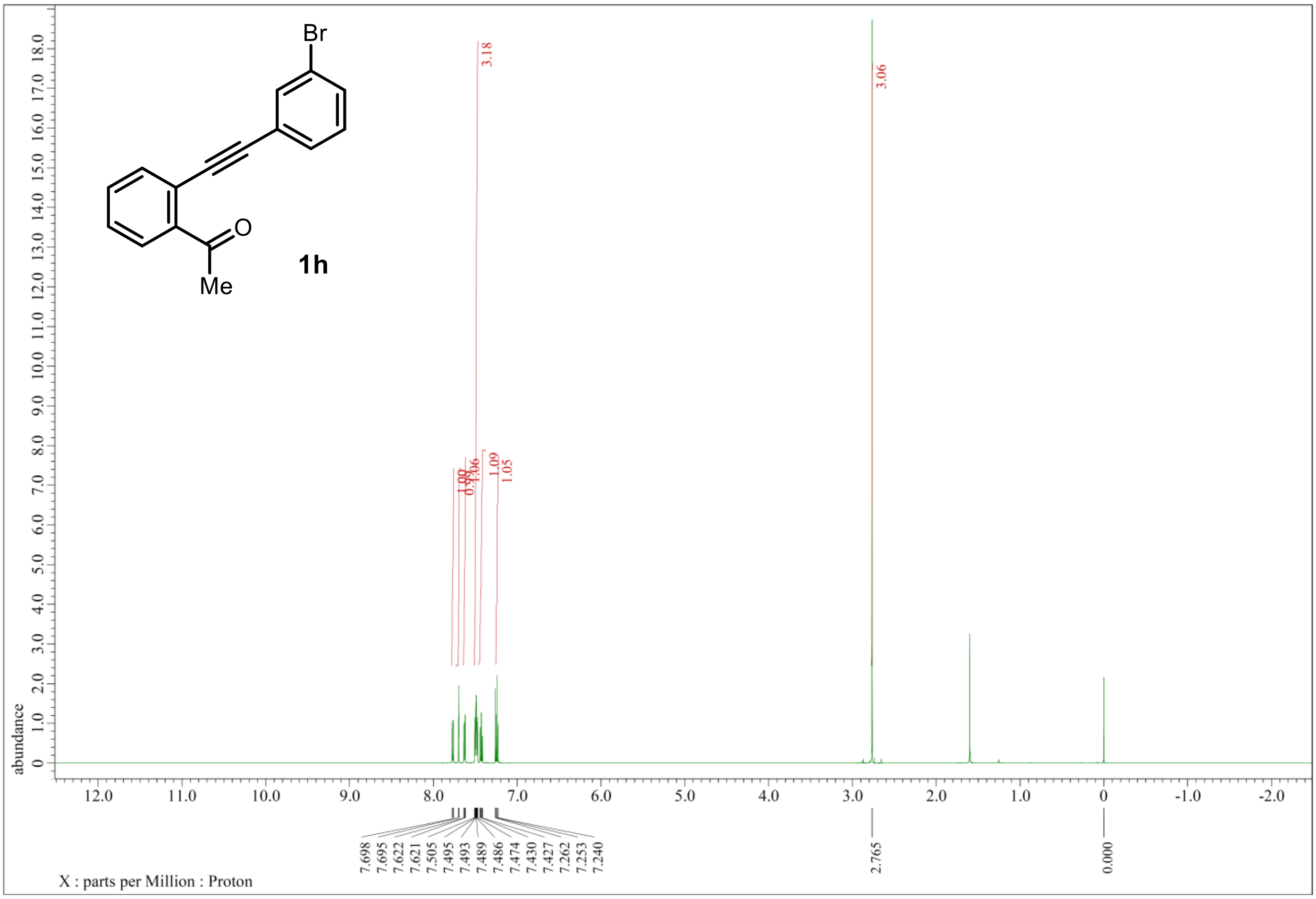

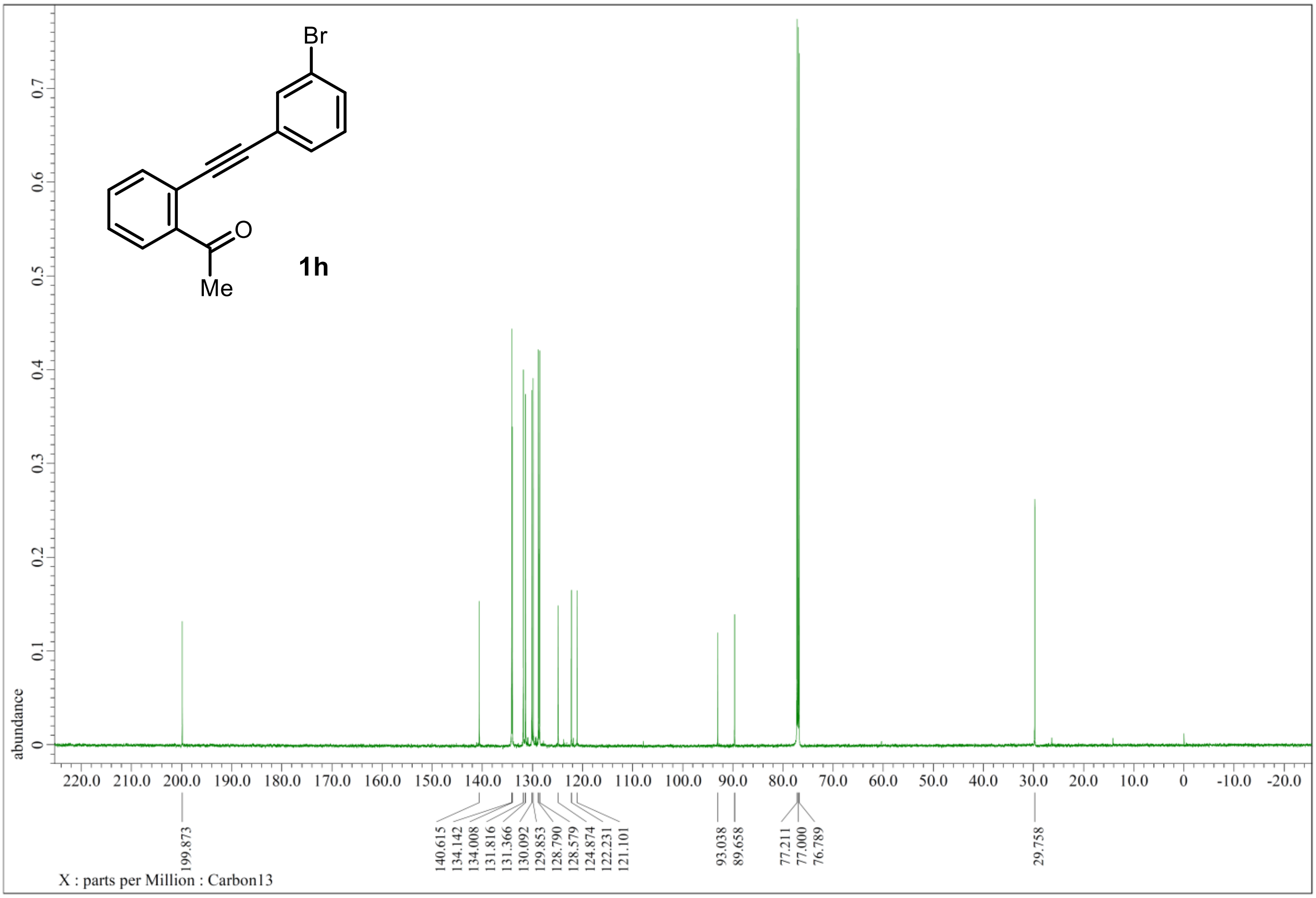

^1H NMR (600 MHz, $CDCl_3$) and $^{13}C\{^1H\}$ NMR (151 MHz, $CDCl_3$) spectra of **1i**.

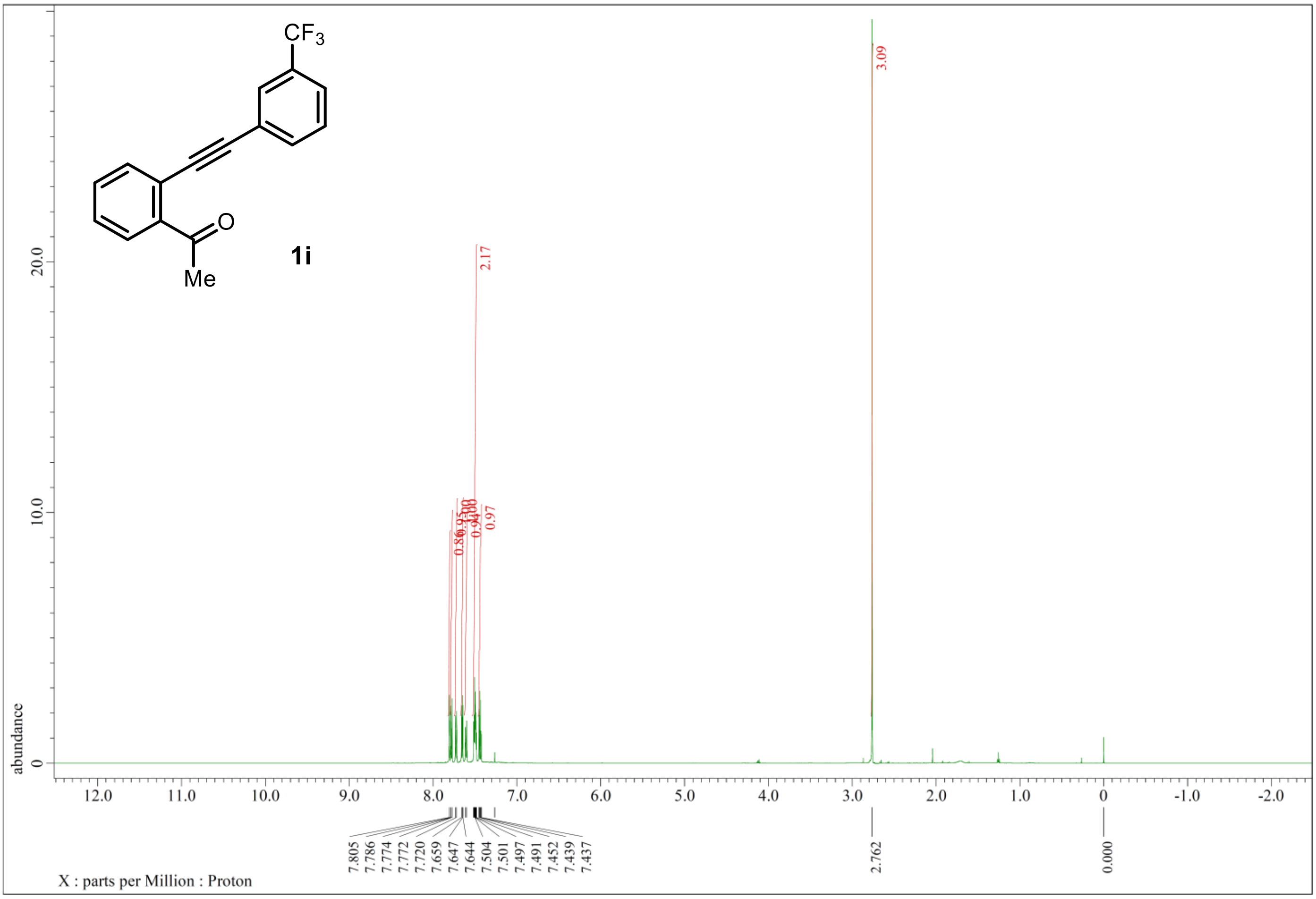

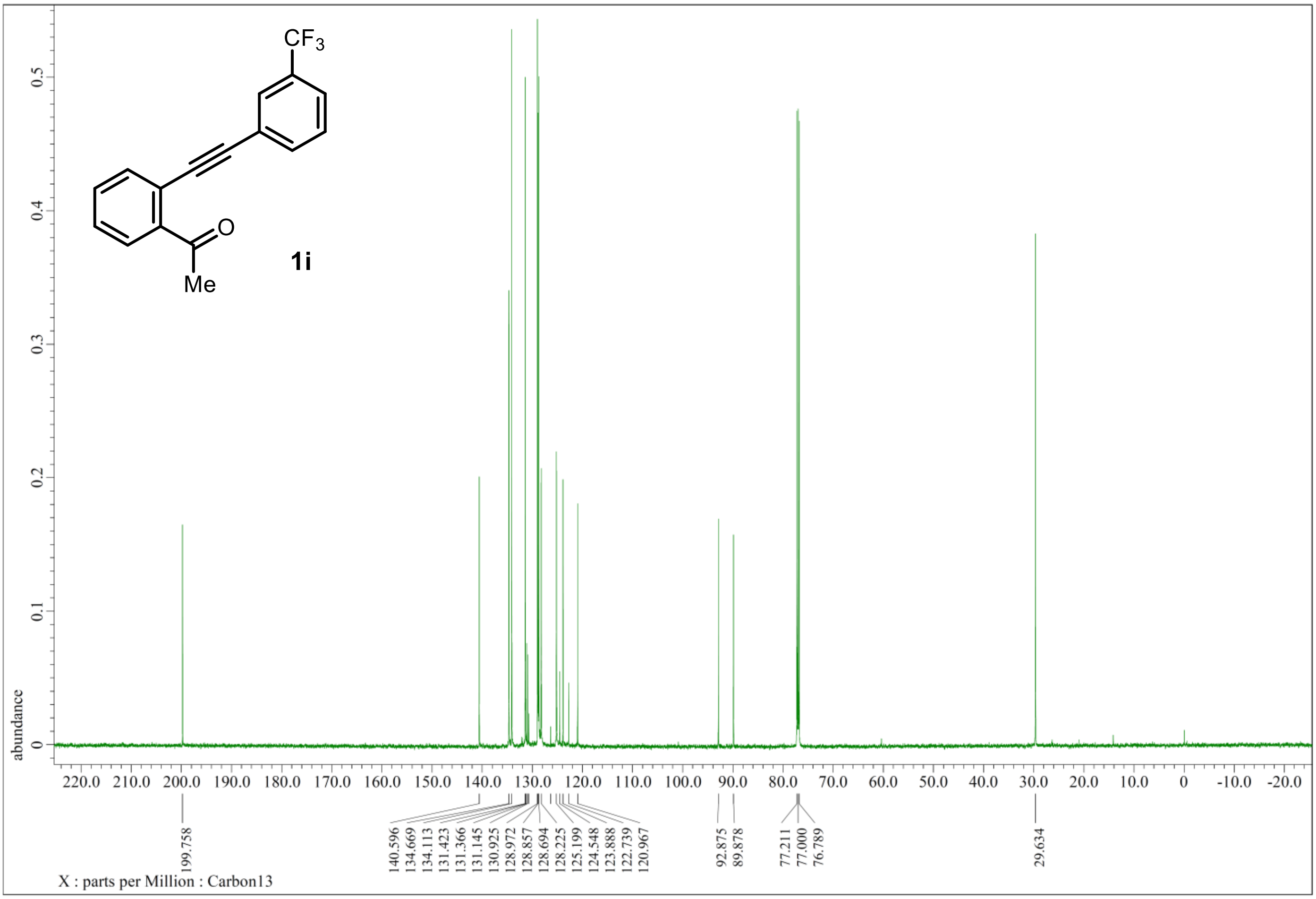

^1H NMR (600 MHz, $CDCl_3$) and $^{13}C\{^1H\}$ NMR (151 MHz, $CDCl_3$) spectra of **1j**.

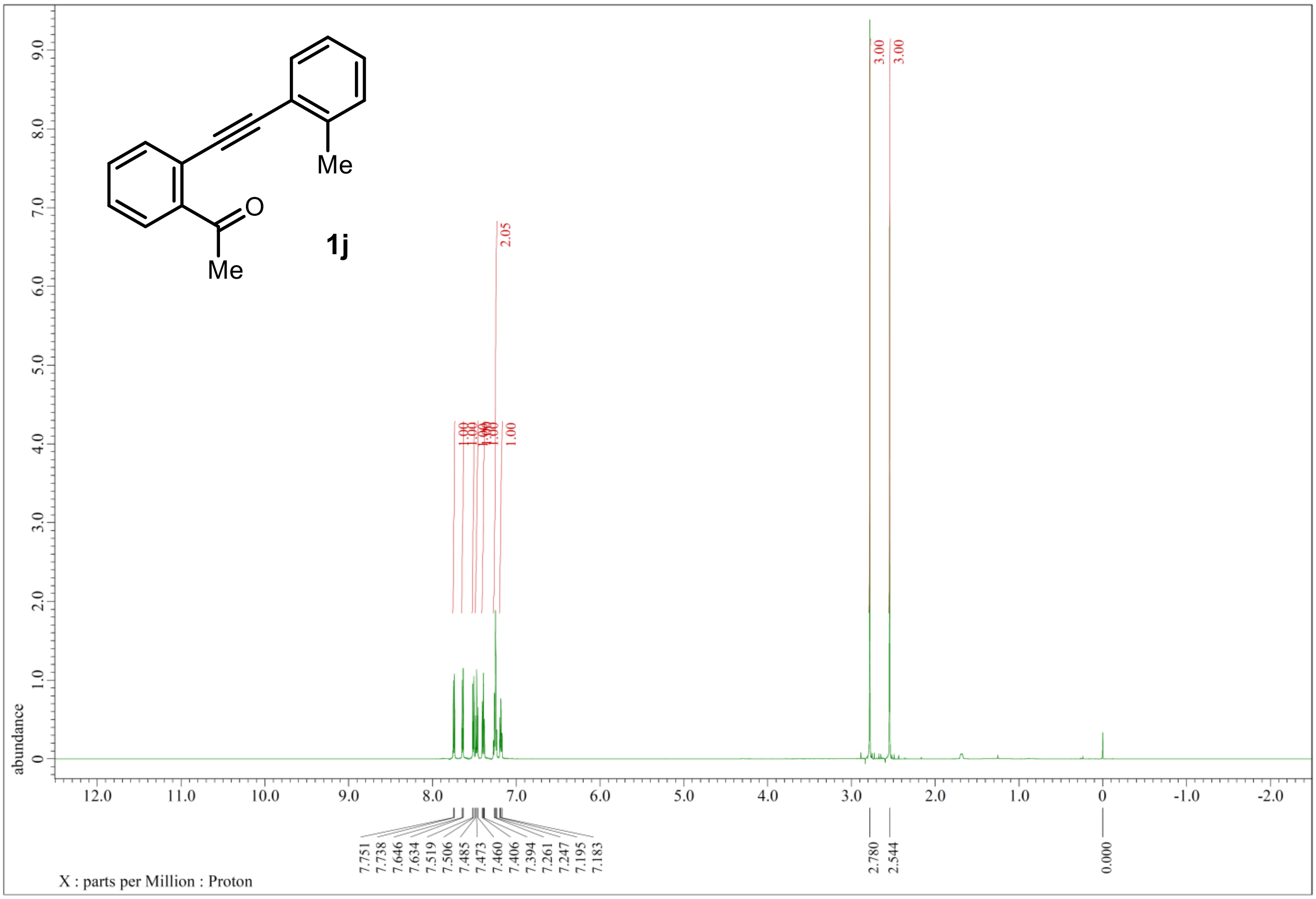

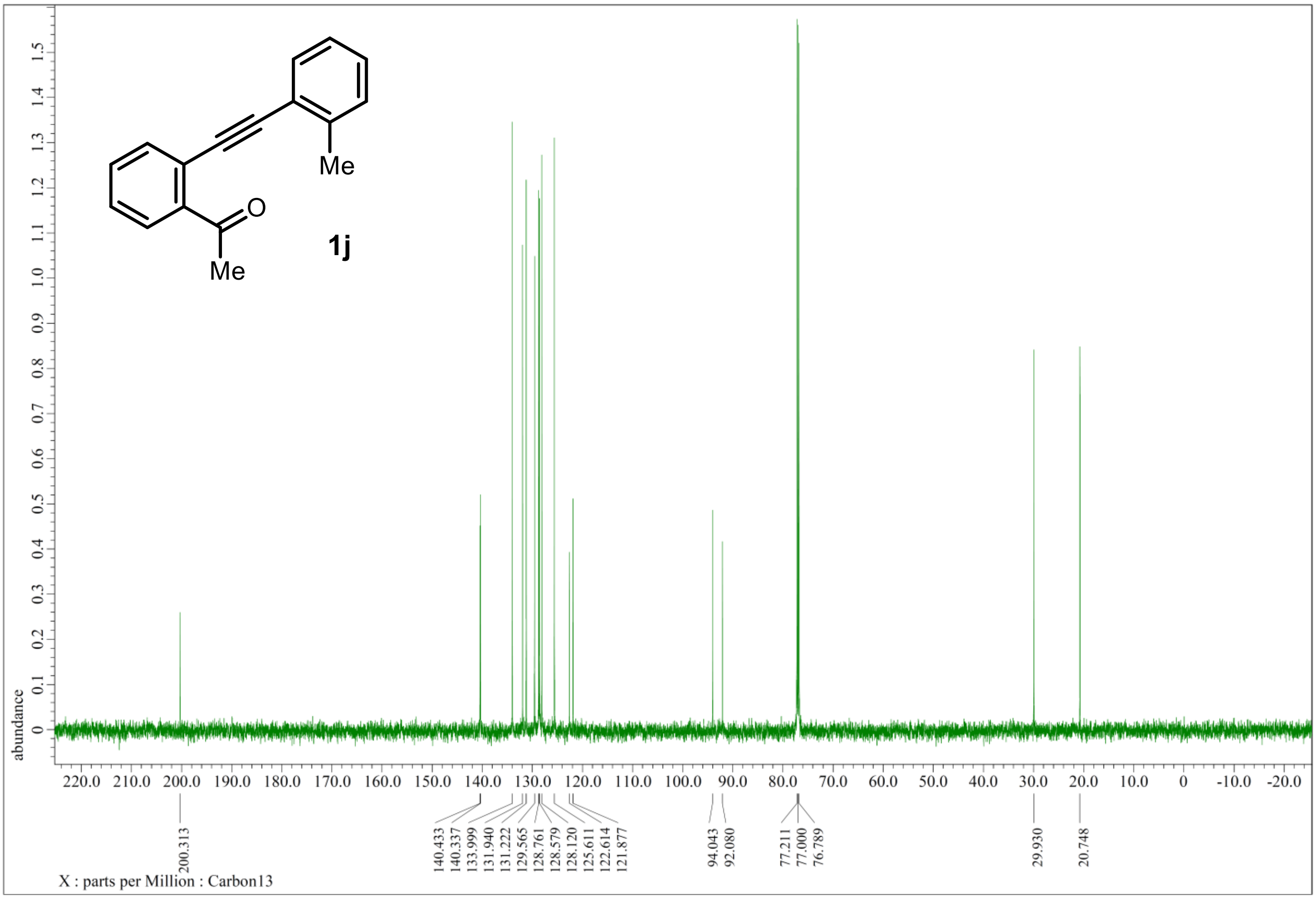

^{1}H NMR (600 MHz, $CDCl_3$) and $^{13}C\{^{1}H\}$ NMR (151 MHz, $CDCl_3$) spectra of **1k**.

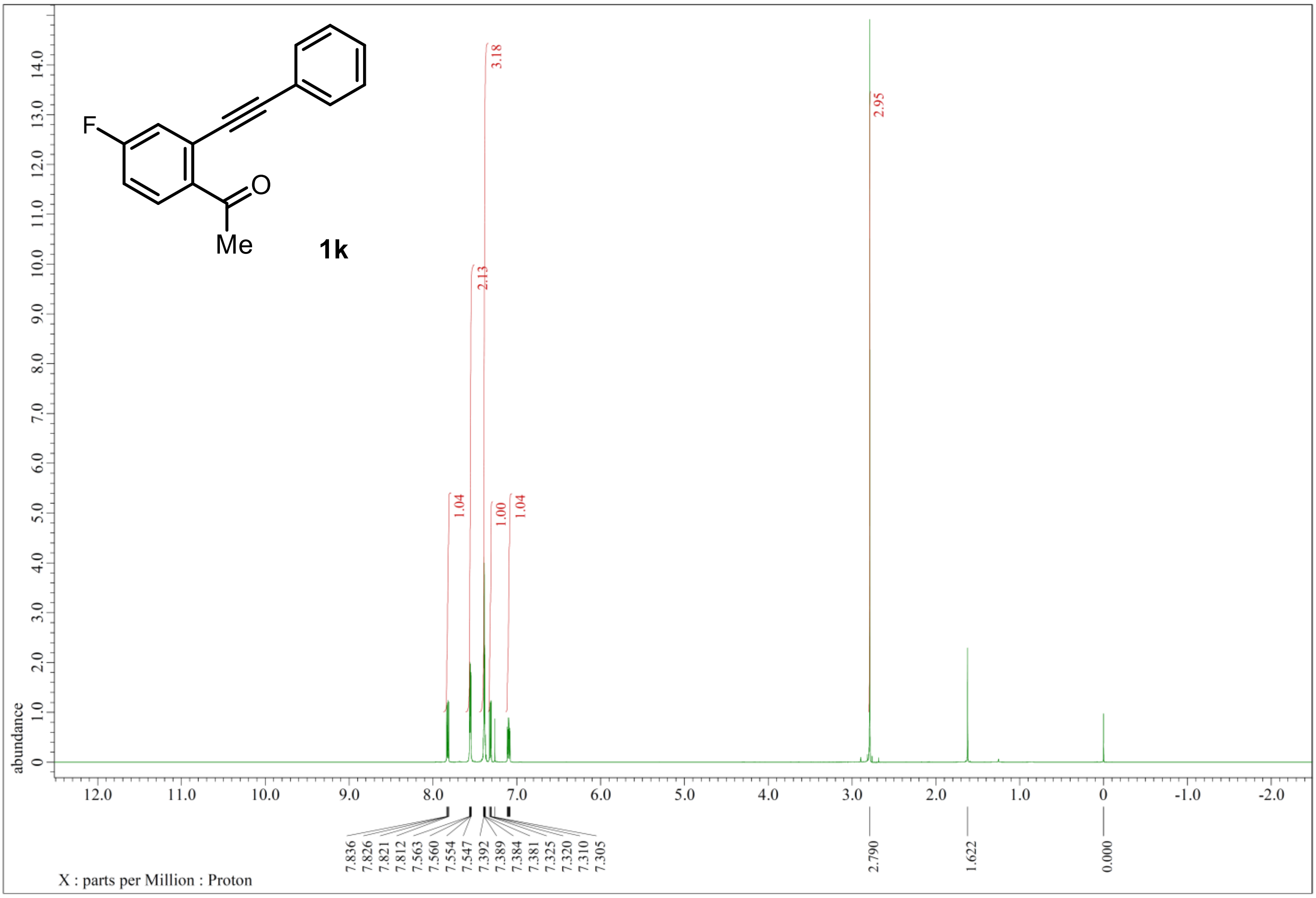

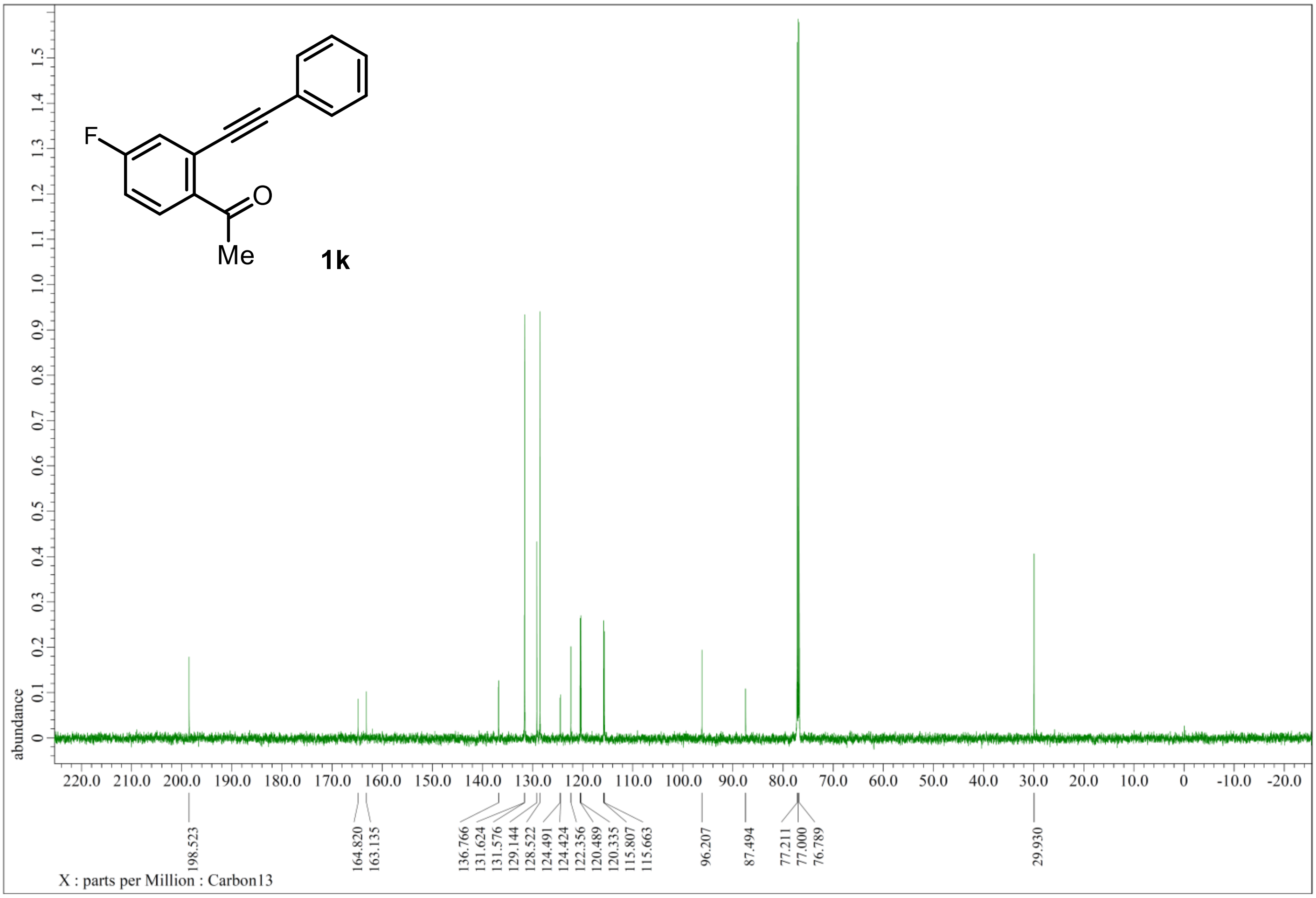

^{1}H NMR (600 MHz, $CDCl_3$) and $^{13}C\{^{1}H\}$ NMR (151 MHz, $CDCl_3$) spectra of **1l**.

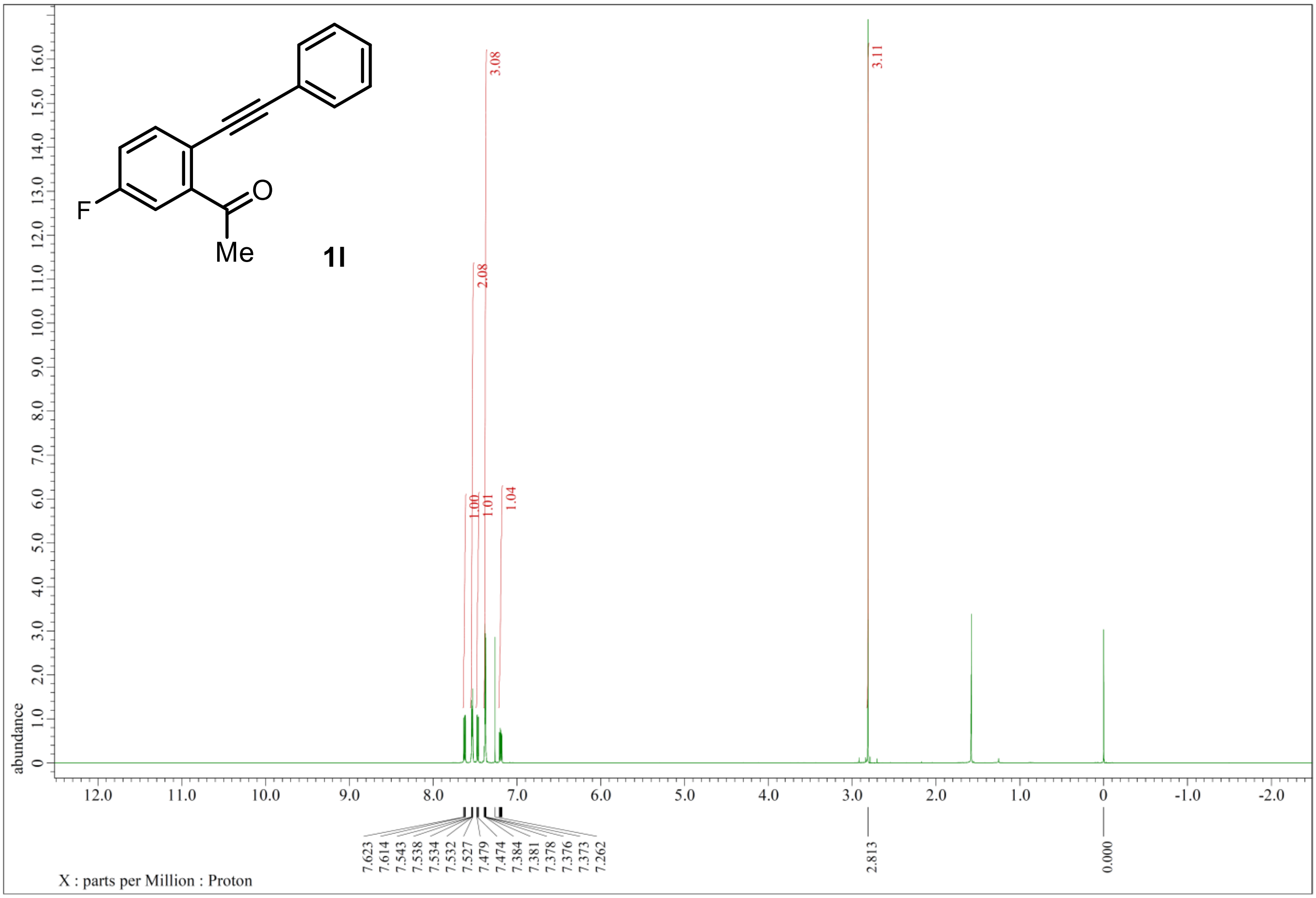

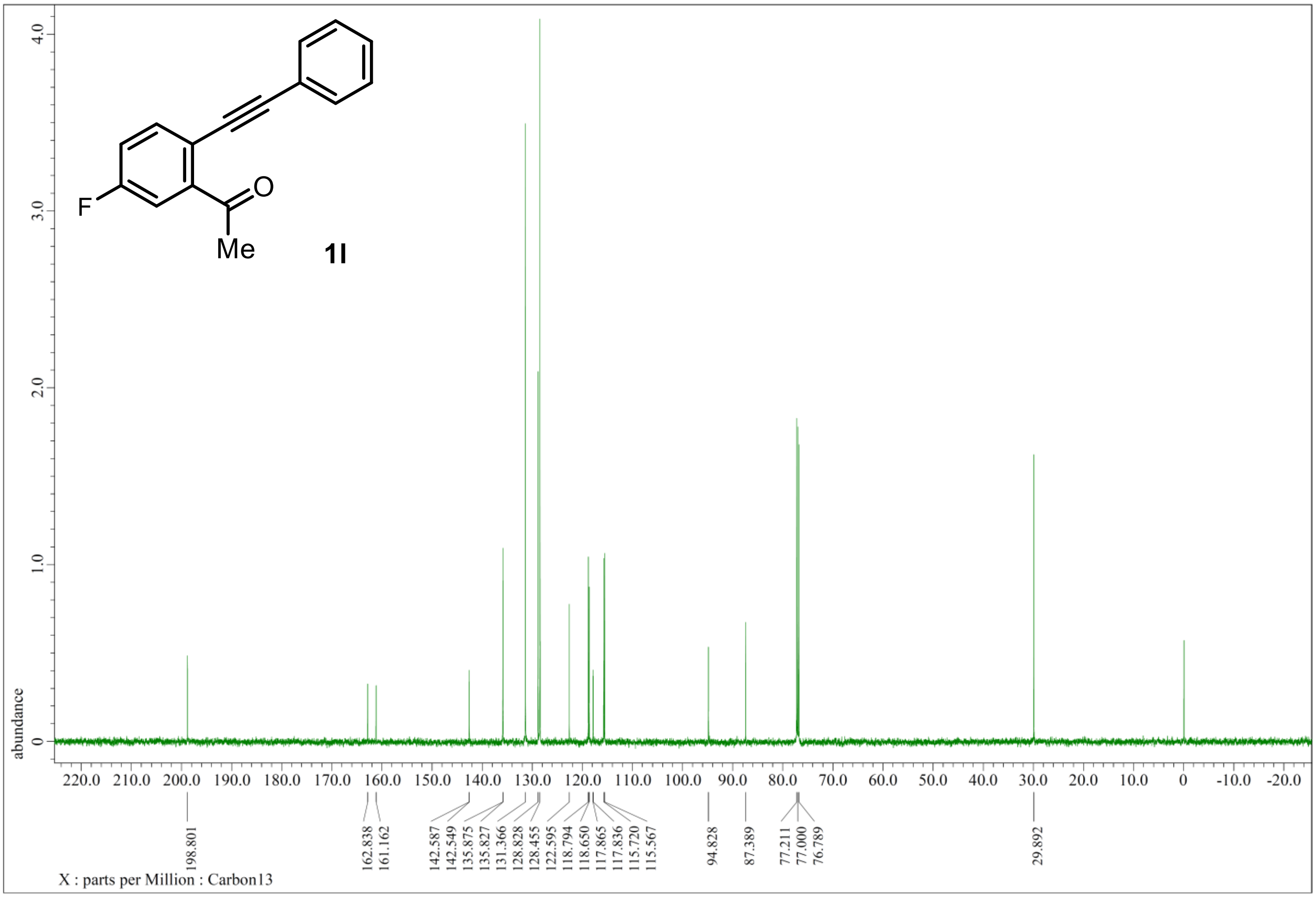

^1H NMR (600 MHz, $CDCl_3$) and $^{13}C\{^1H\}$ NMR (151 MHz, $CDCl_3$) spectra of **1m**.

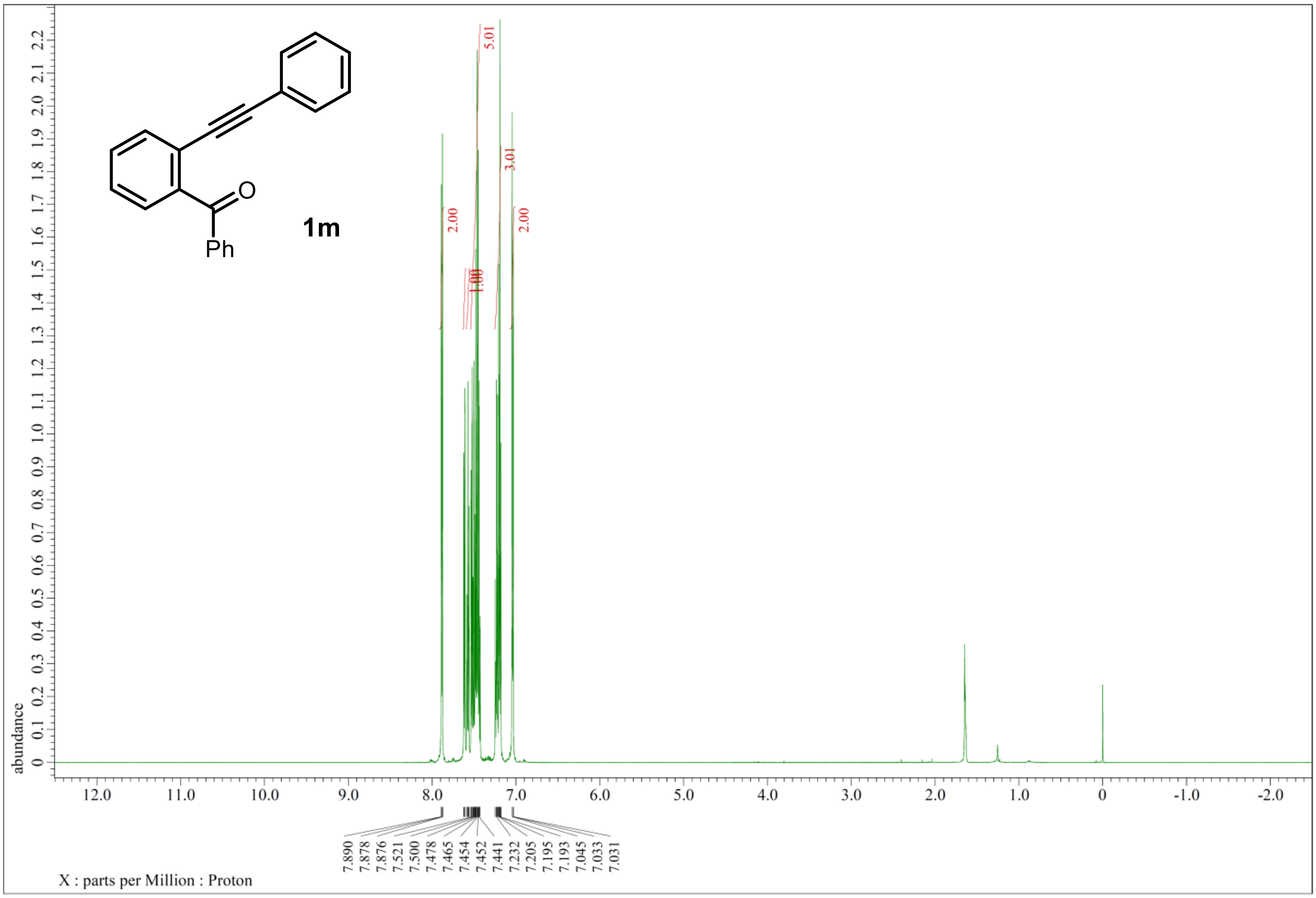

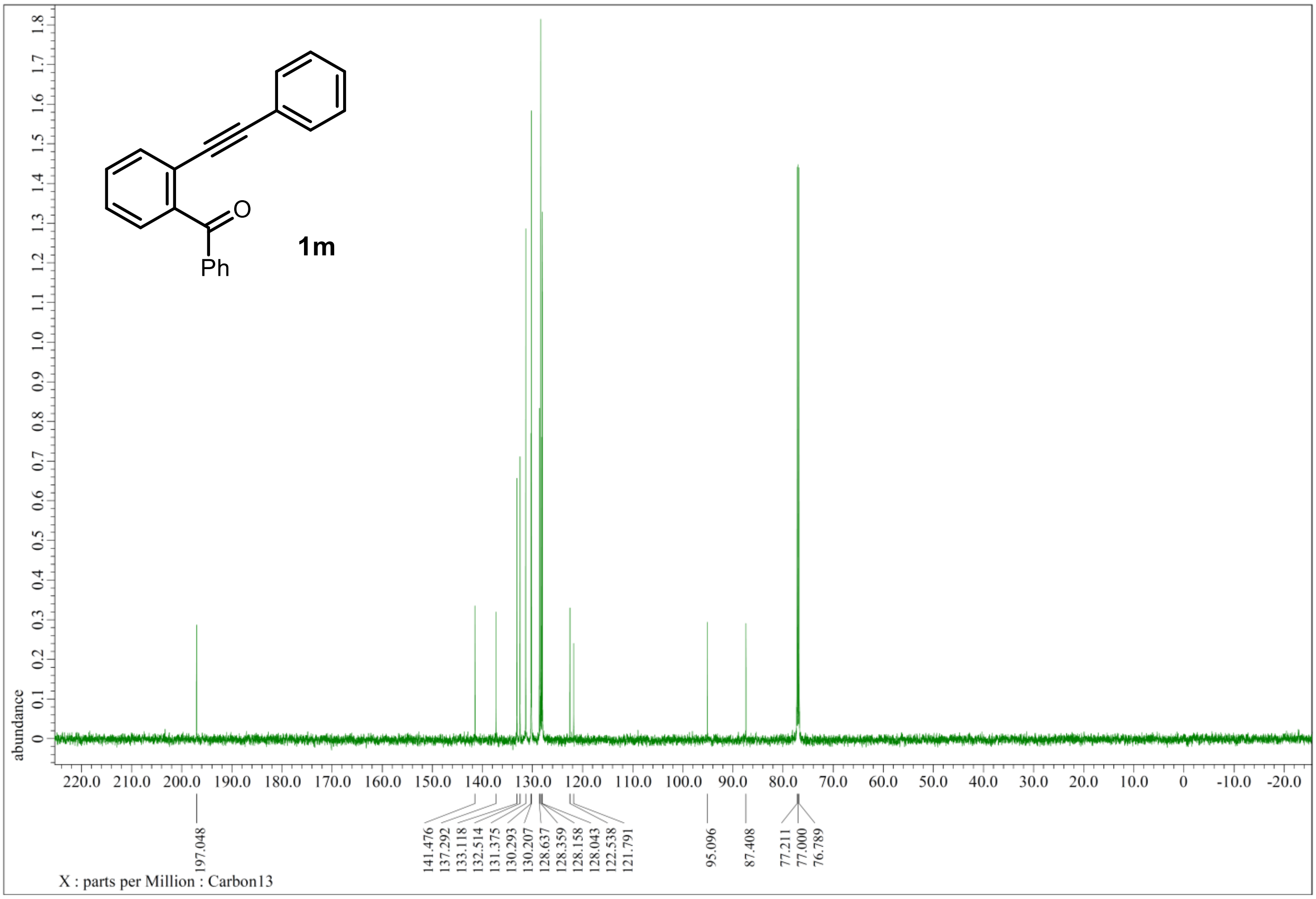

^1H NMR (600 MHz, $CDCl_3$) and $^{13}C\{^1H\}$ NMR (151 MHz, $CDCl_3$) spectra of **1n**.

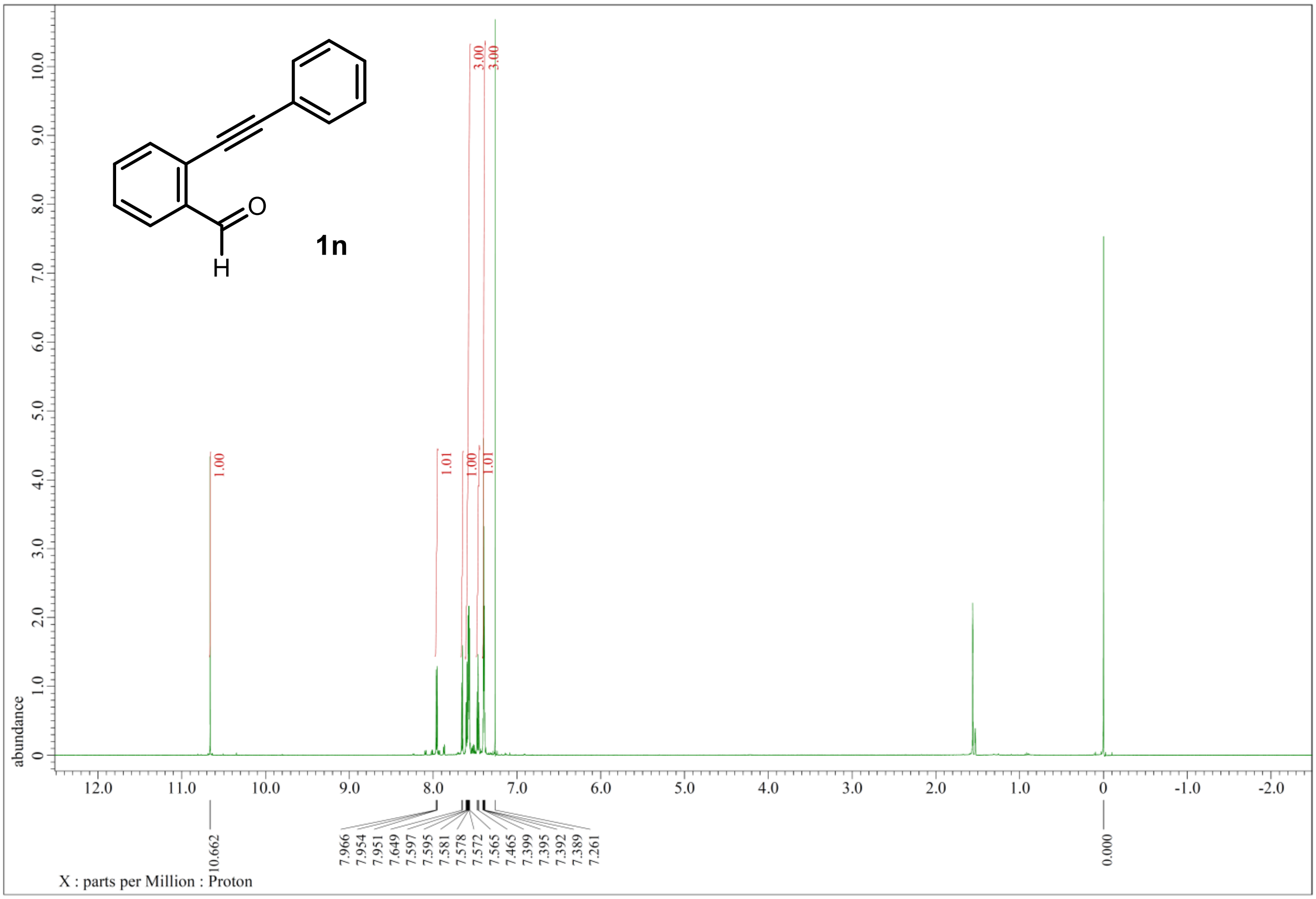

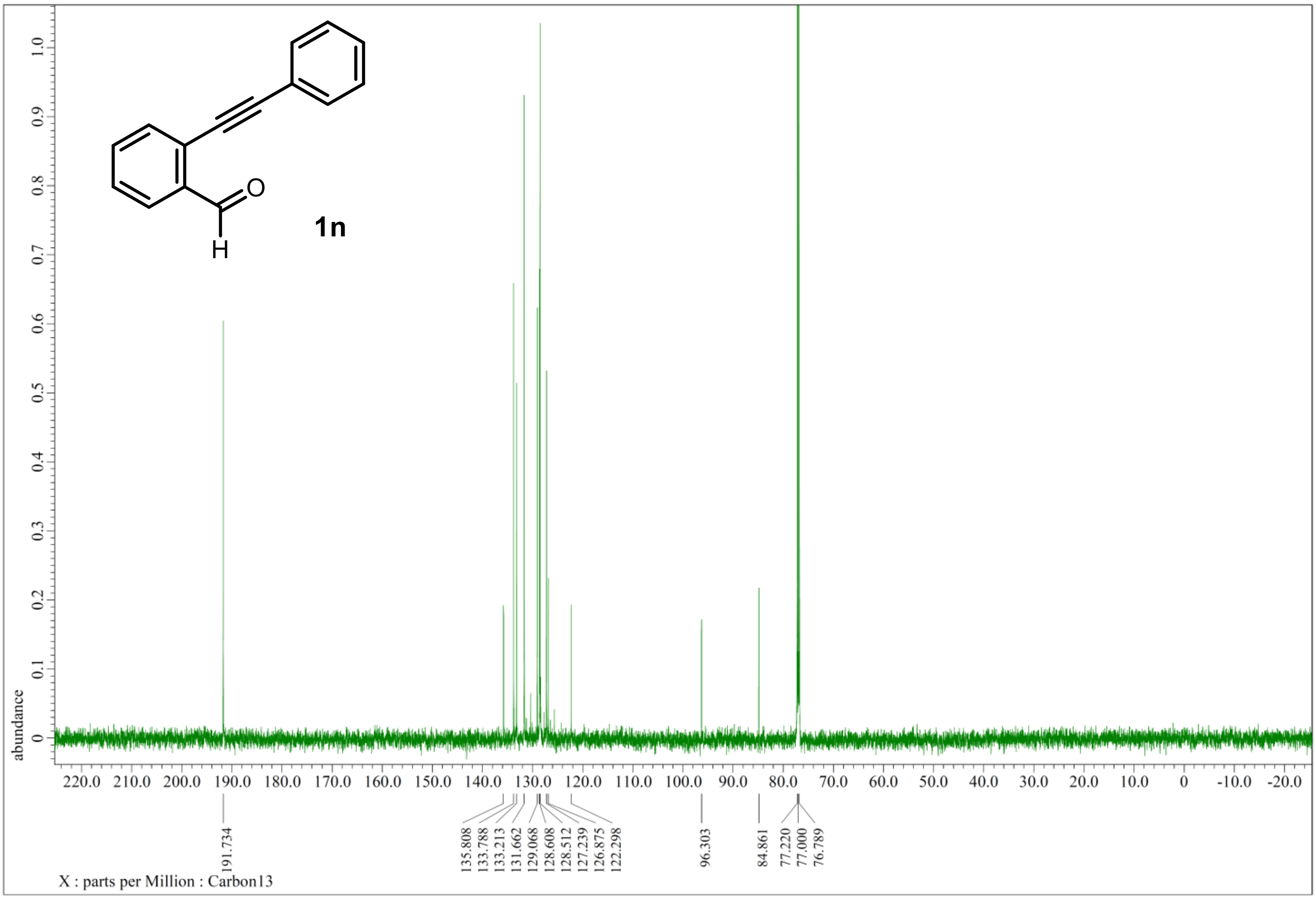

^{1}H NMR (600 MHz, $CDCl_3$) and $^{13}C\{^{1}H\}$ NMR (151 MHz, $CDCl_3$) spectra of **1o**.

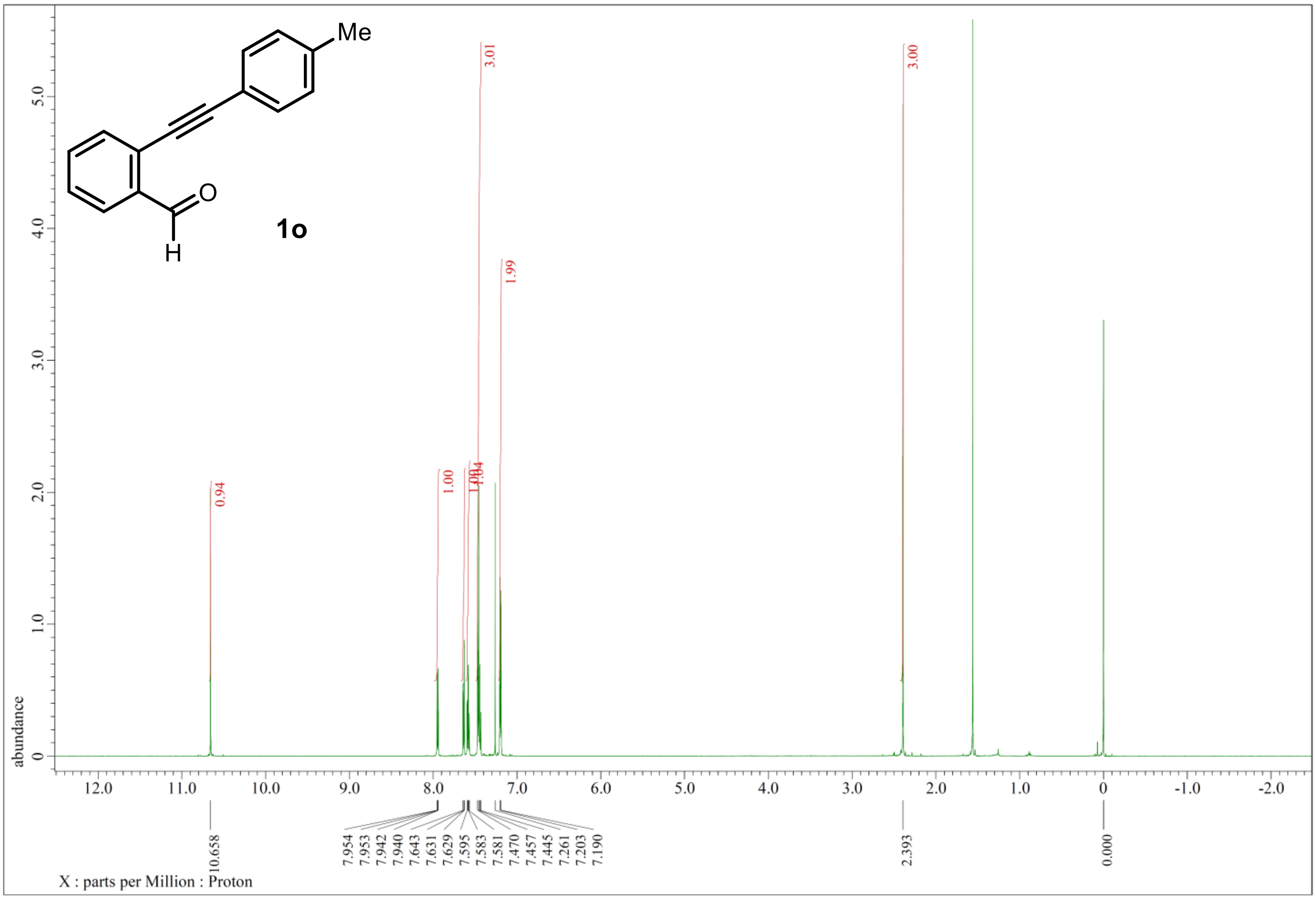

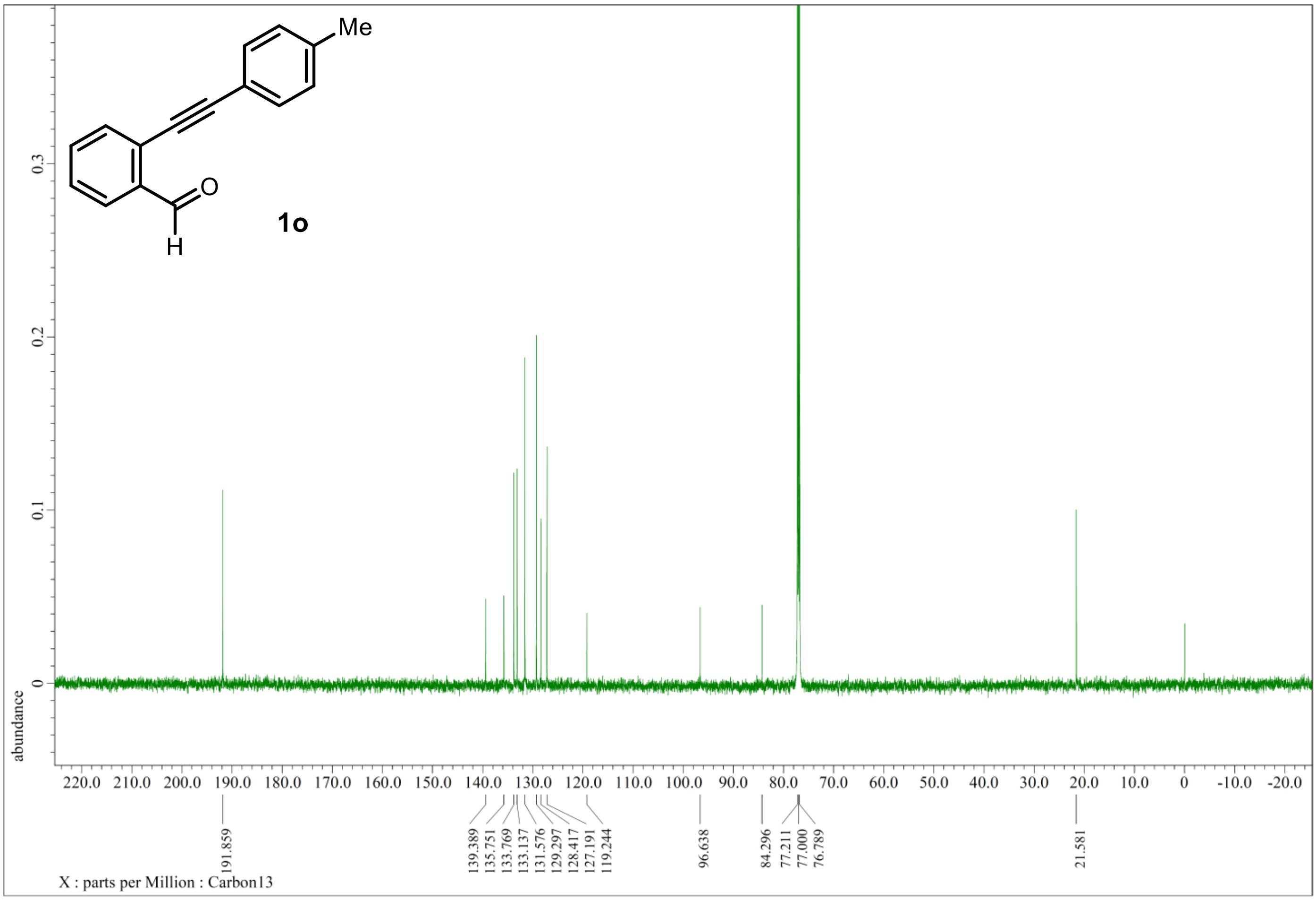

^{1}H NMR (600 MHz, $CDCl_3$) and $^{13}C\{^{1}H\}$ NMR (151 MHz, $CDCl_3$) spectra of **1p**.

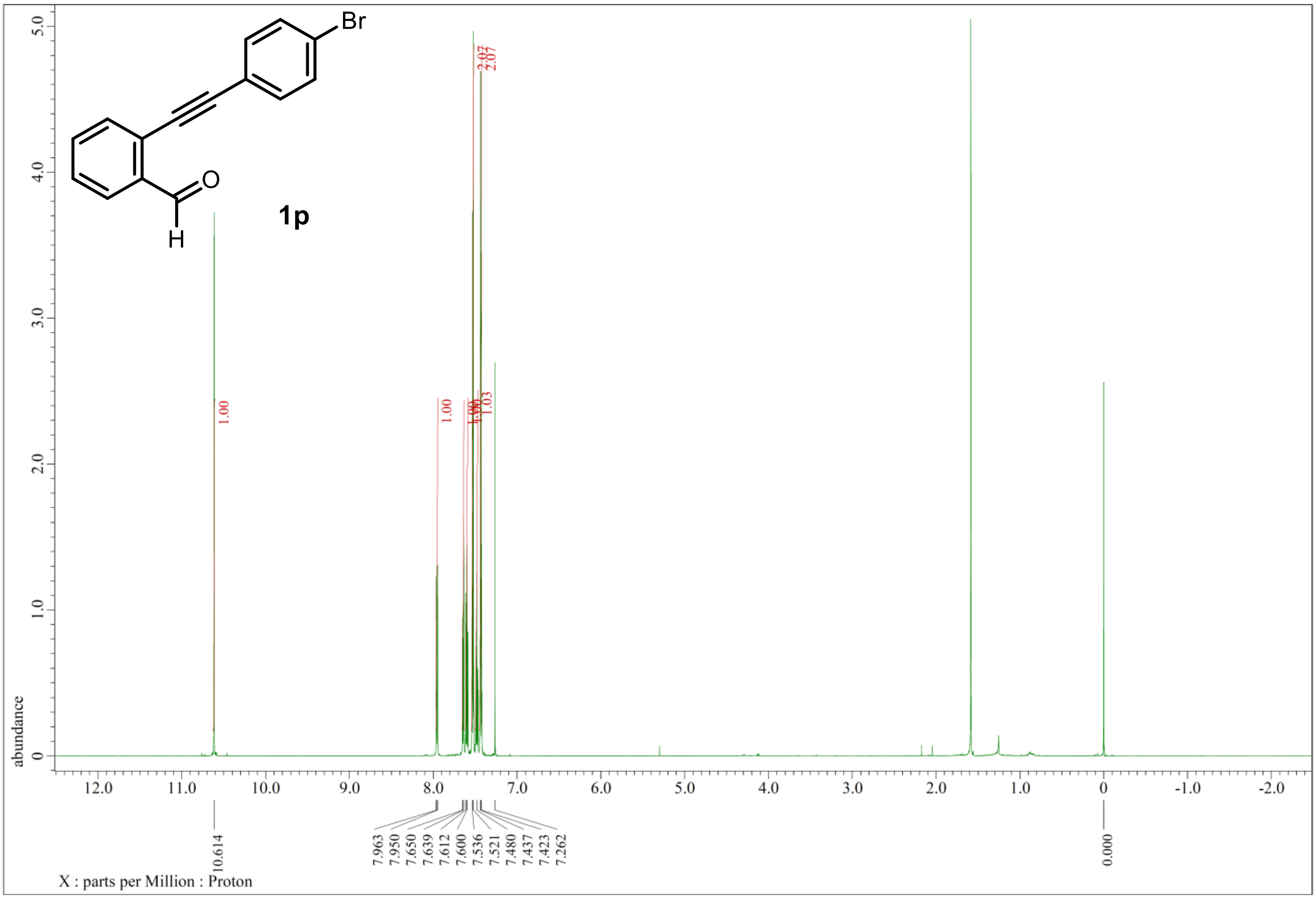

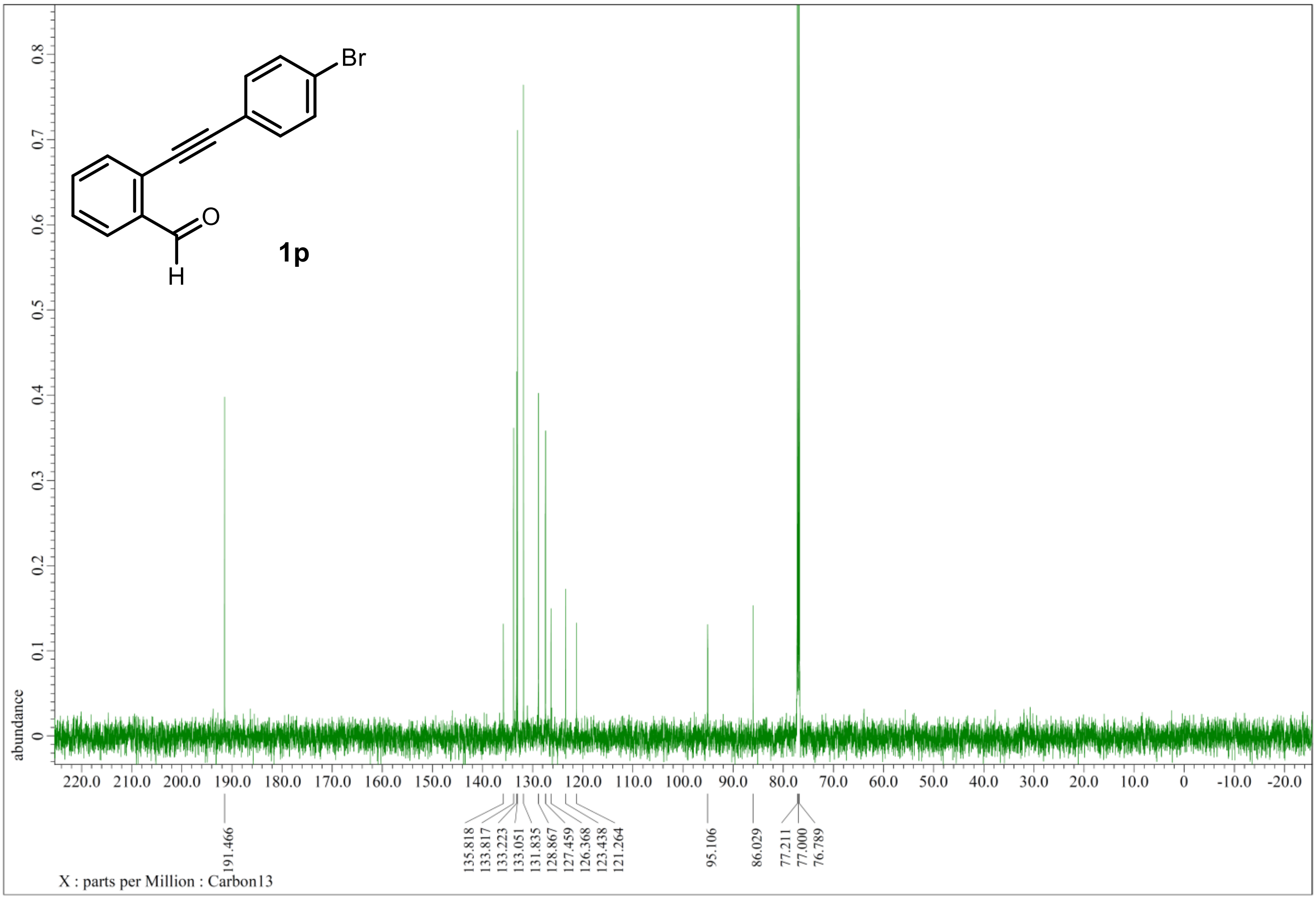

^{1}H NMR (600 MHz, $CDCl_3$) and $^{13}C\{^1H\}$ NMR (151 MHz, $CDCl_3$) spectra of **1q**.

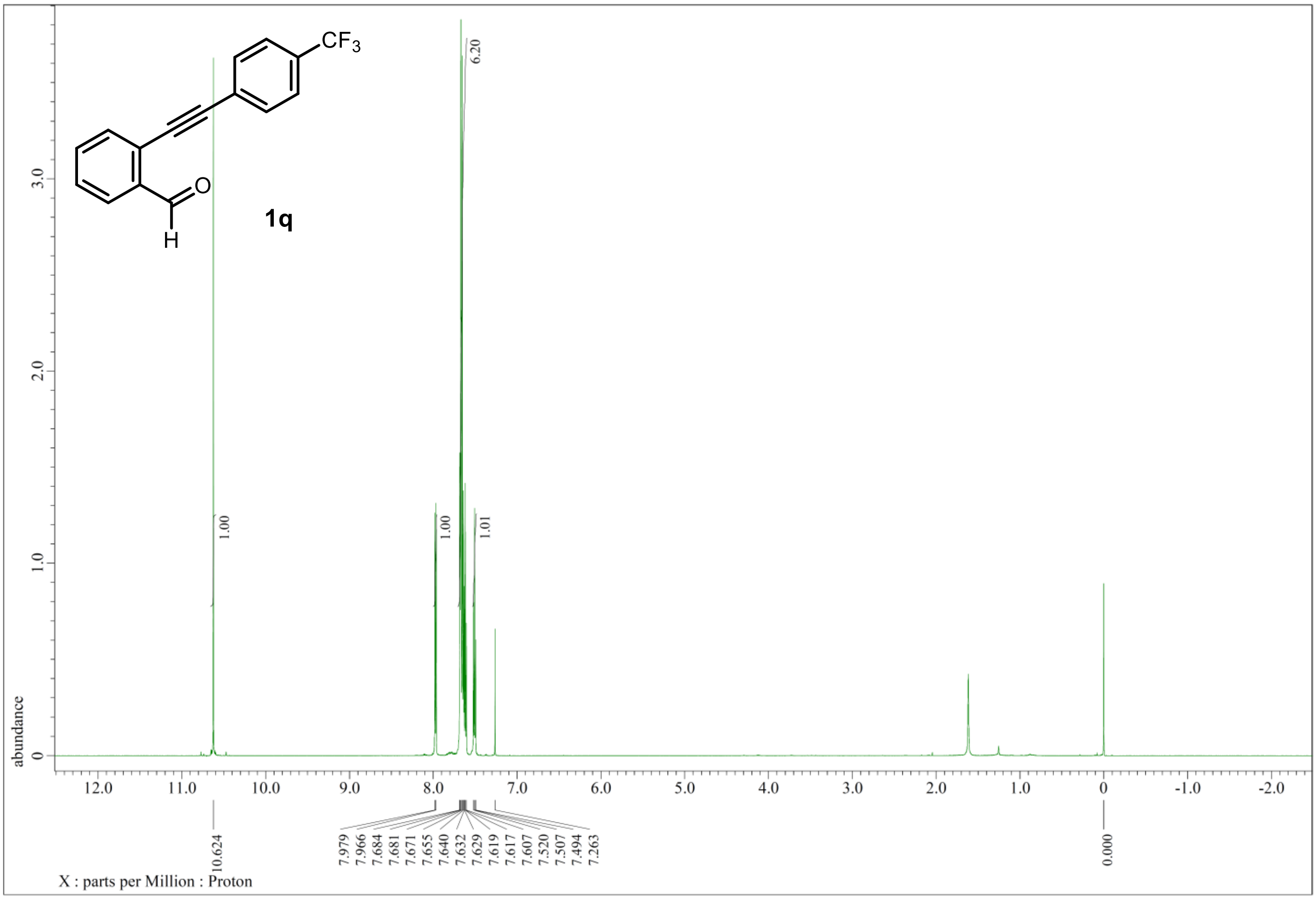

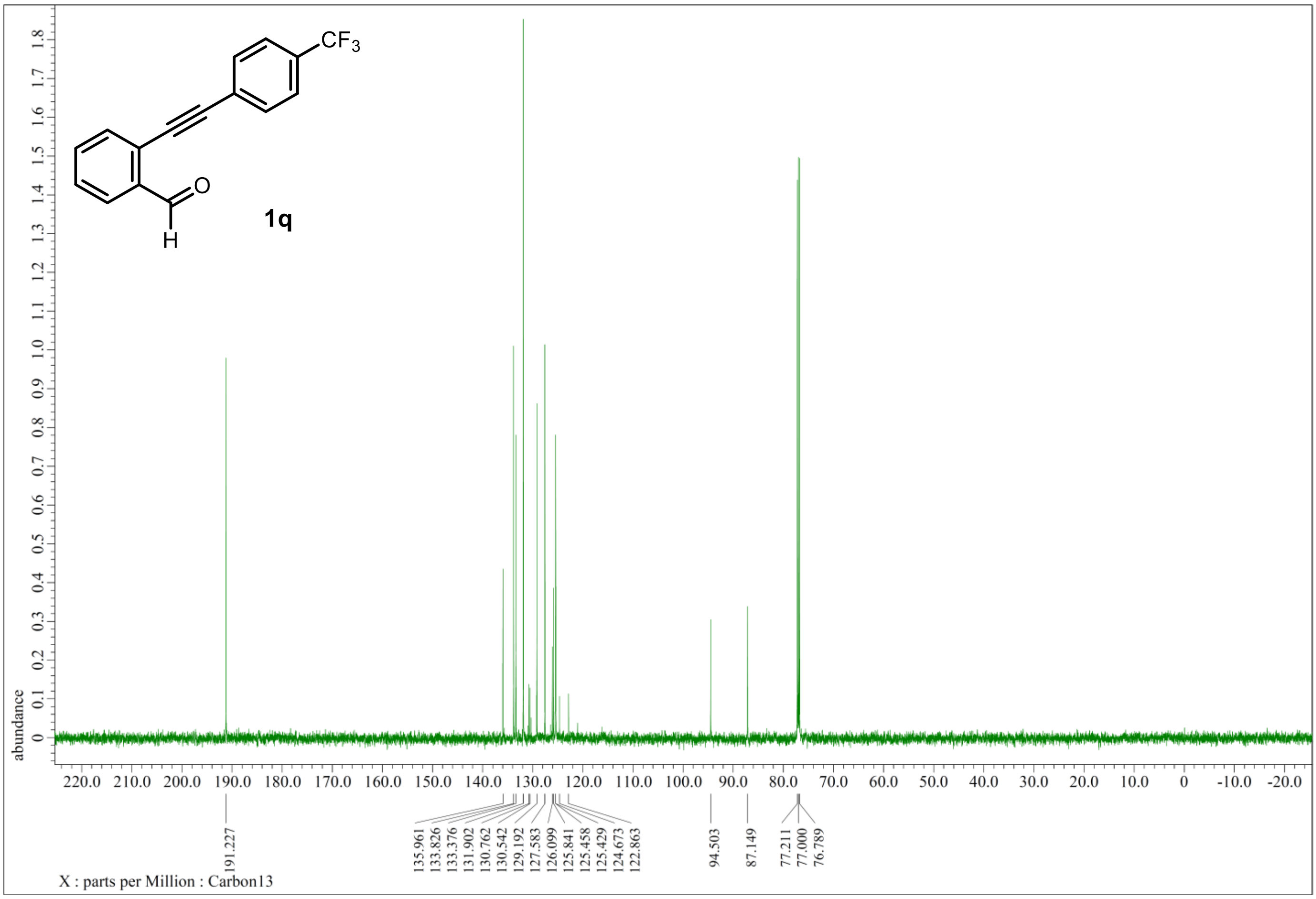

^{1}H NMR (600 MHz, $CDCl_3$) and $^{13}C\{^{1}H\}$ NMR (151 MHz, $CDCl_3$) spectra of **1r**.

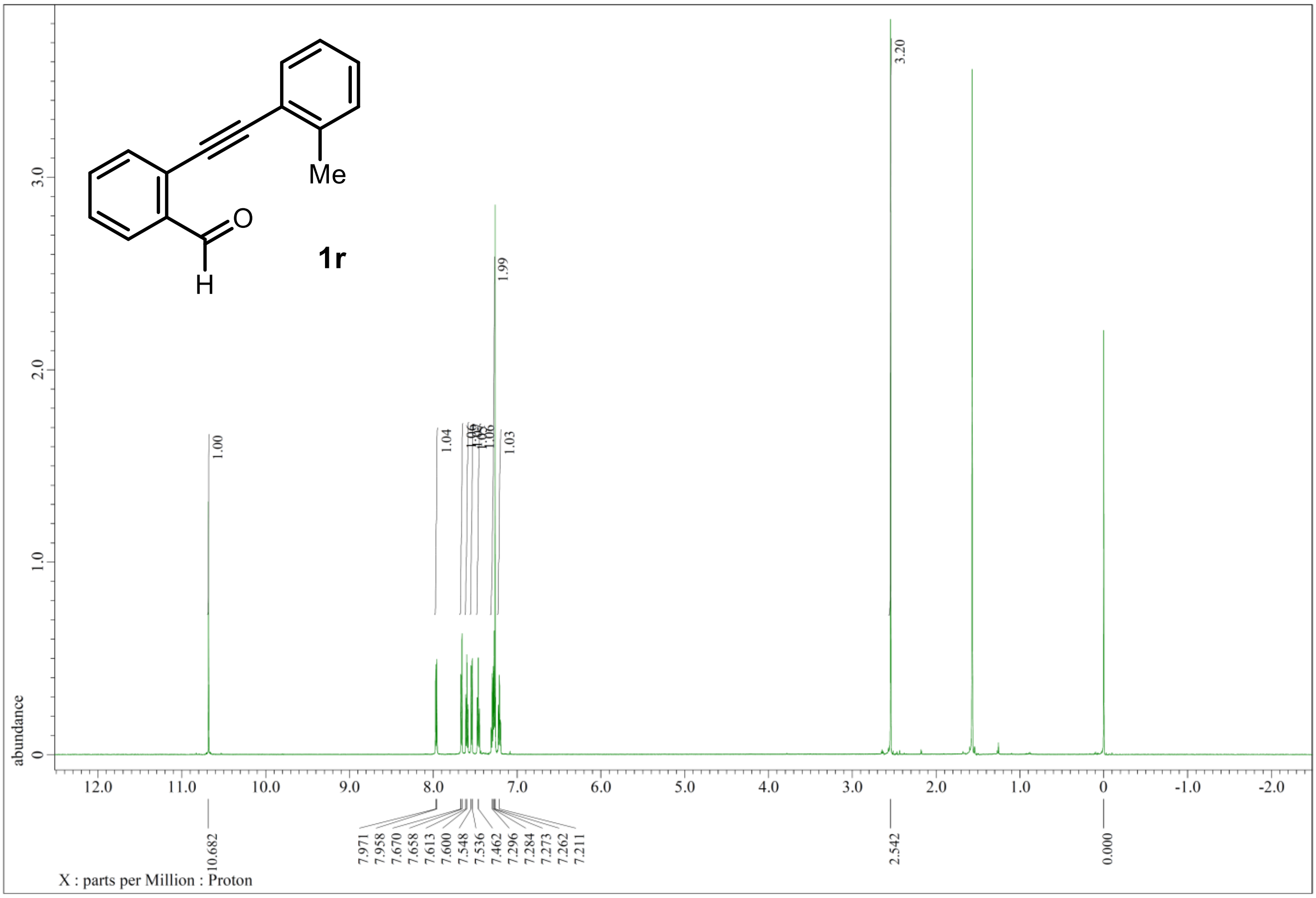

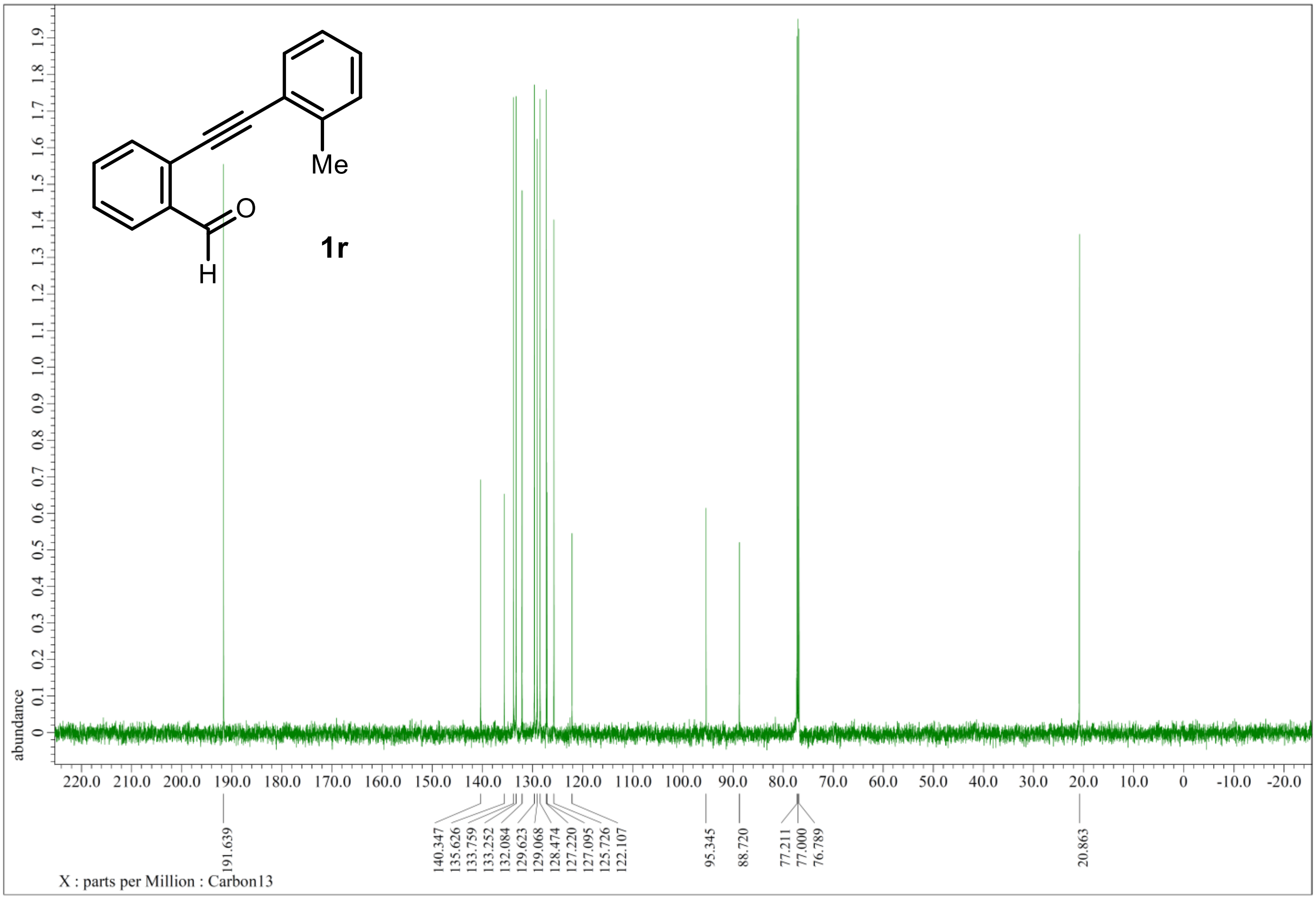

^1H NMR (600 MHz, $CDCl_3$) and $^{13}C\{^1H\}$ NMR (151 MHz, $CDCl_3$) spectra of **3a**.

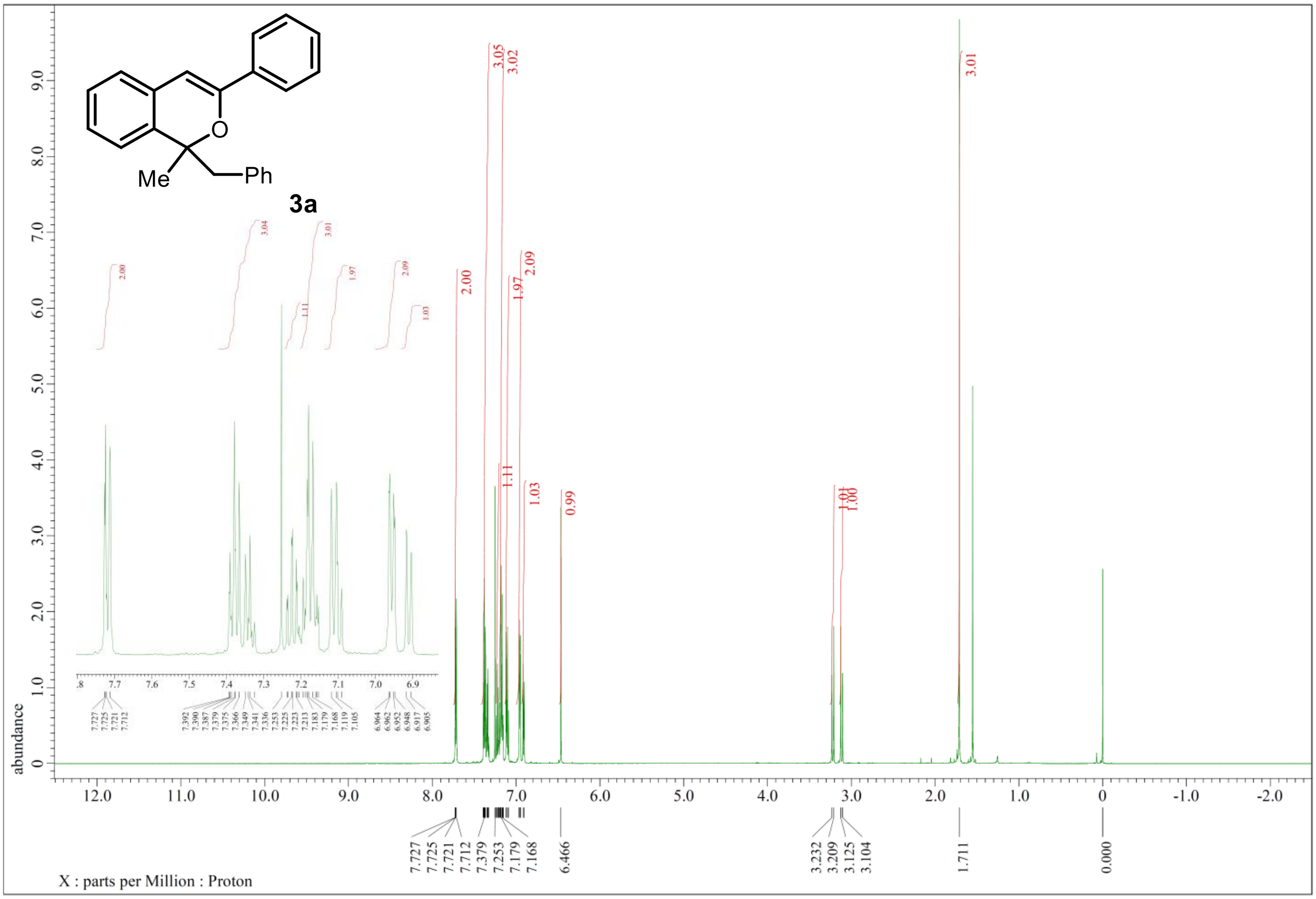

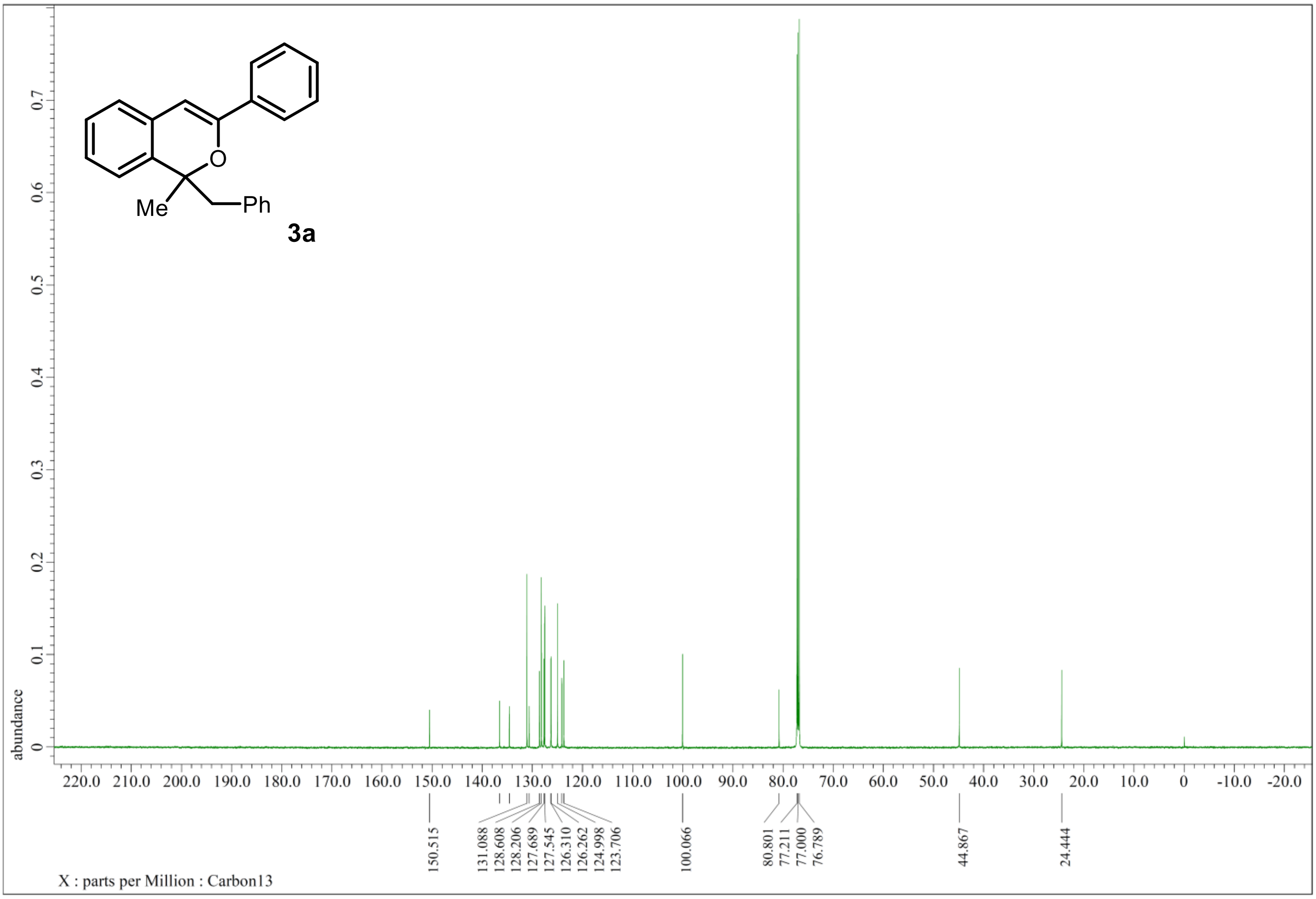

^1H NMR (600 MHz, $CDCl_3$) and $^{13}C\{^1H\}$ NMR (151 MHz, $CDCl_3$) spectra of **3b**.

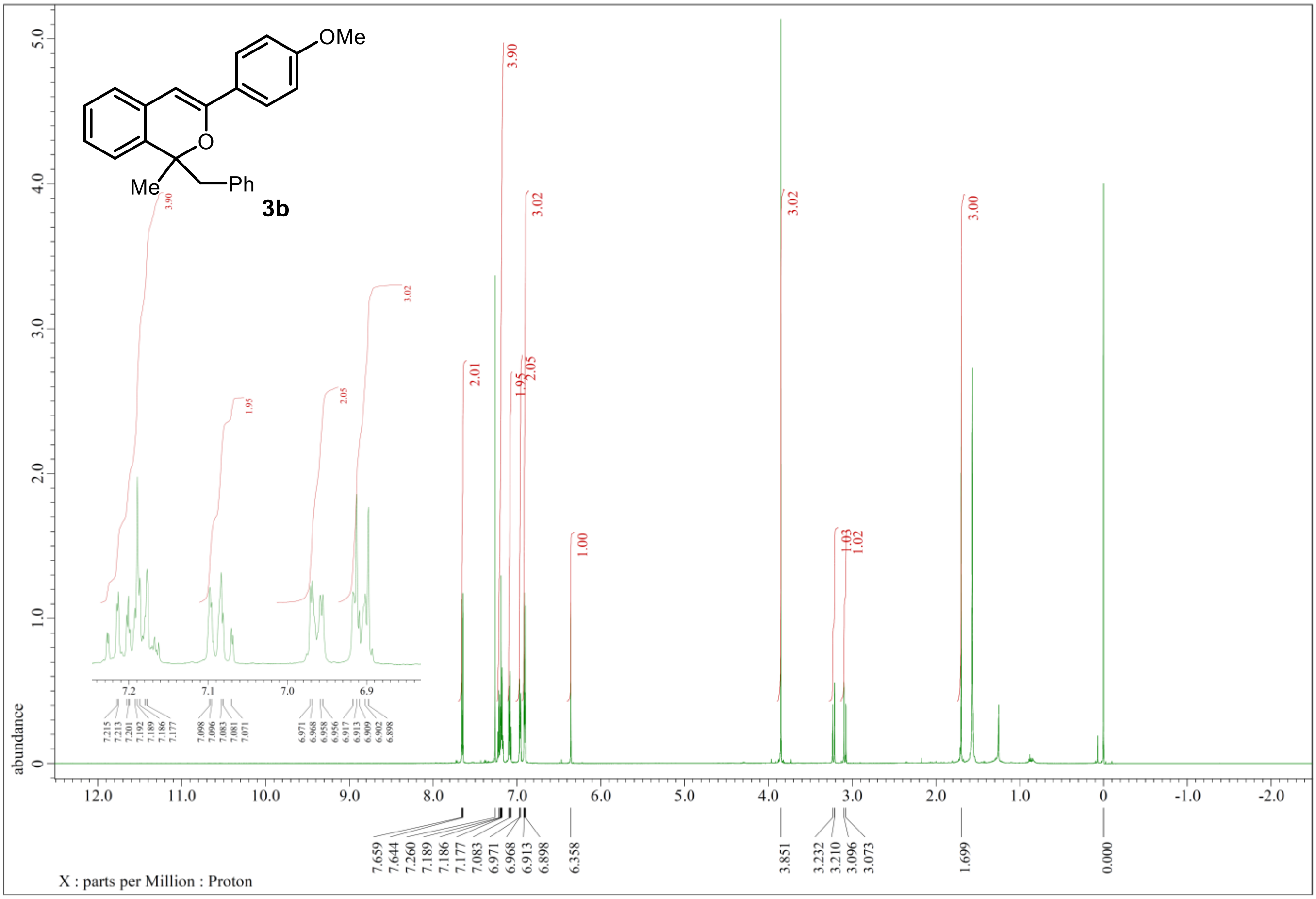

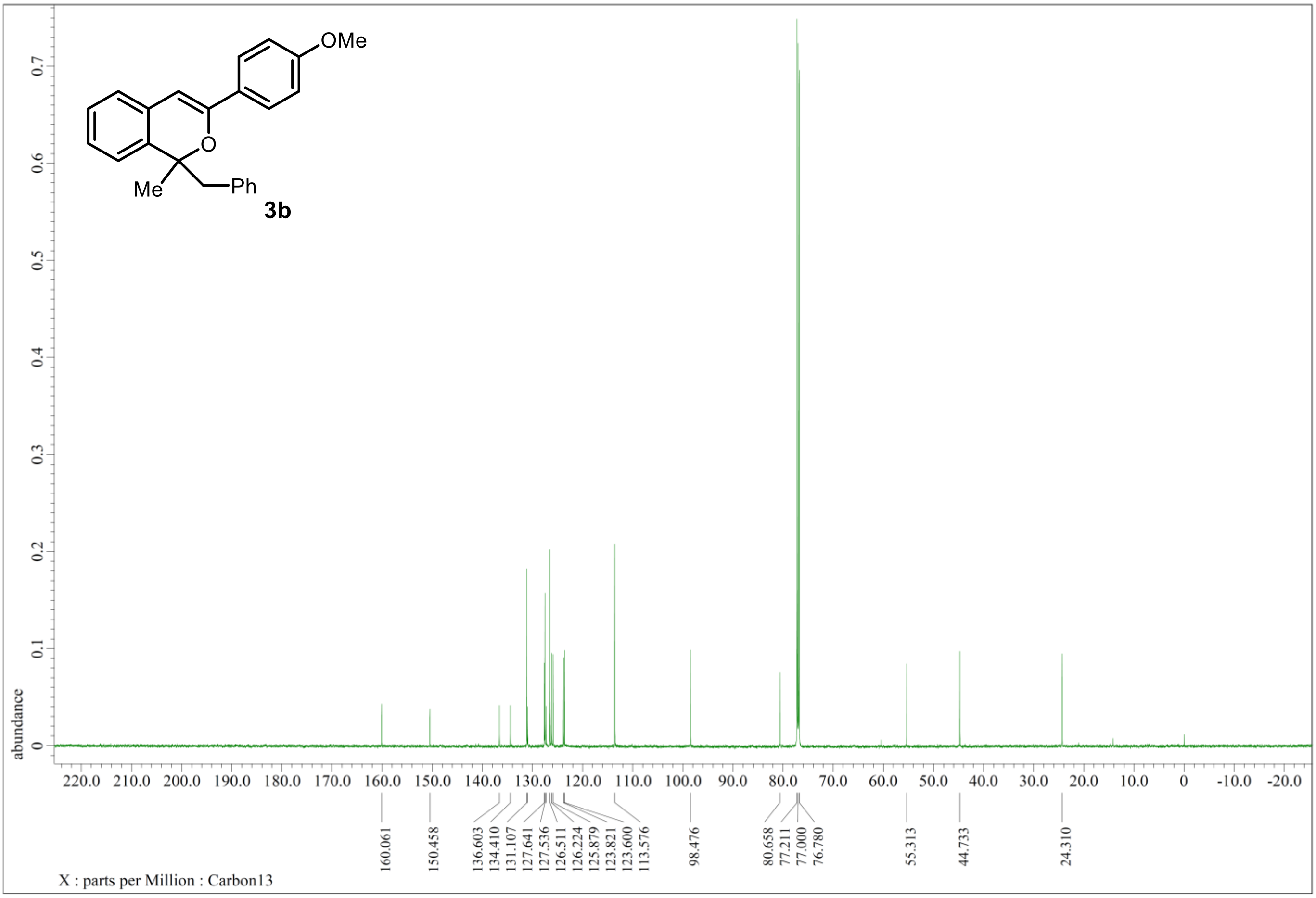

^{1}H NMR (600 MHz, $CDCl_3$) and ^{13}C{^{1}H} NMR (151 MHz, $CDCl_3$) spectra of **3c**.

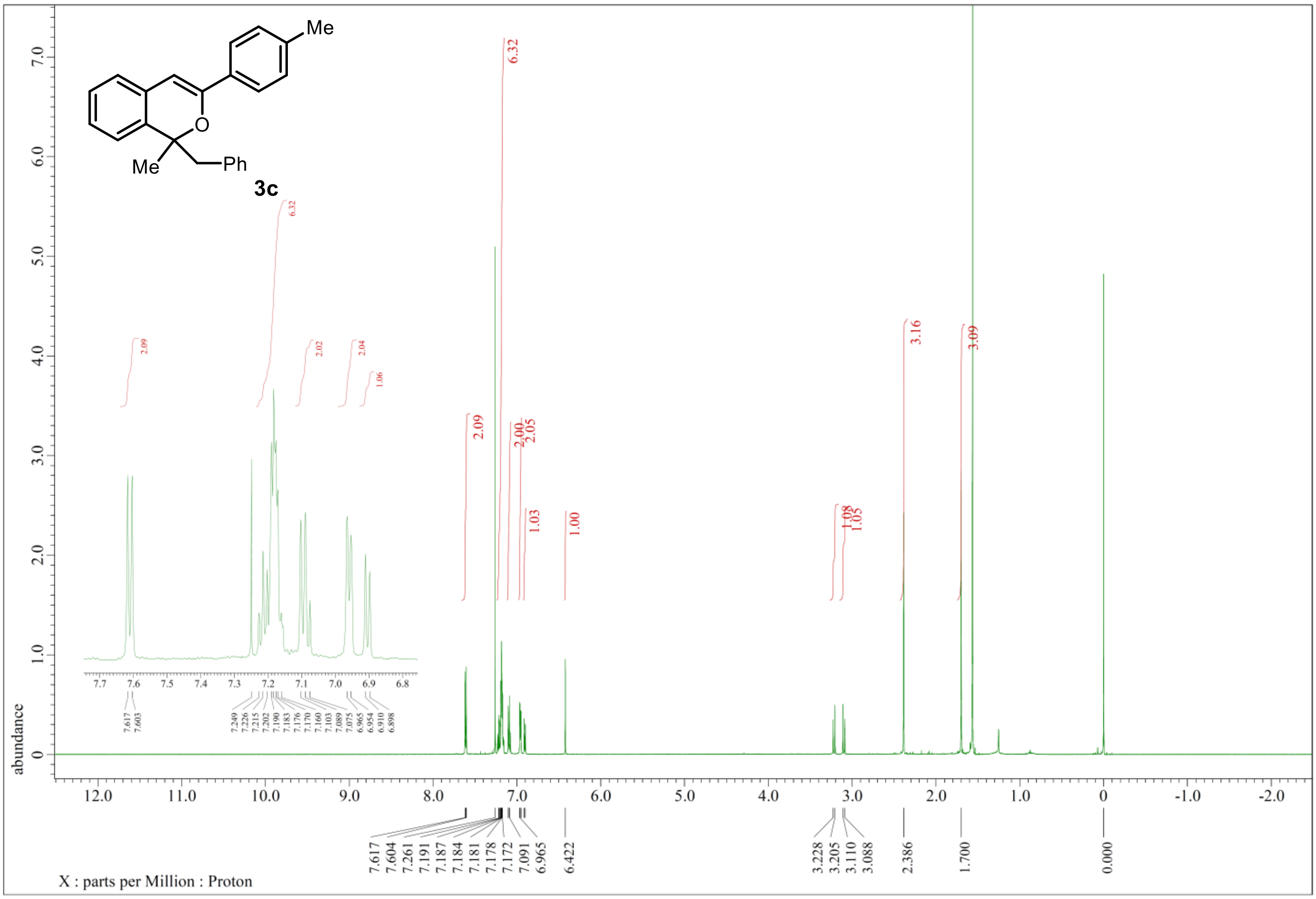

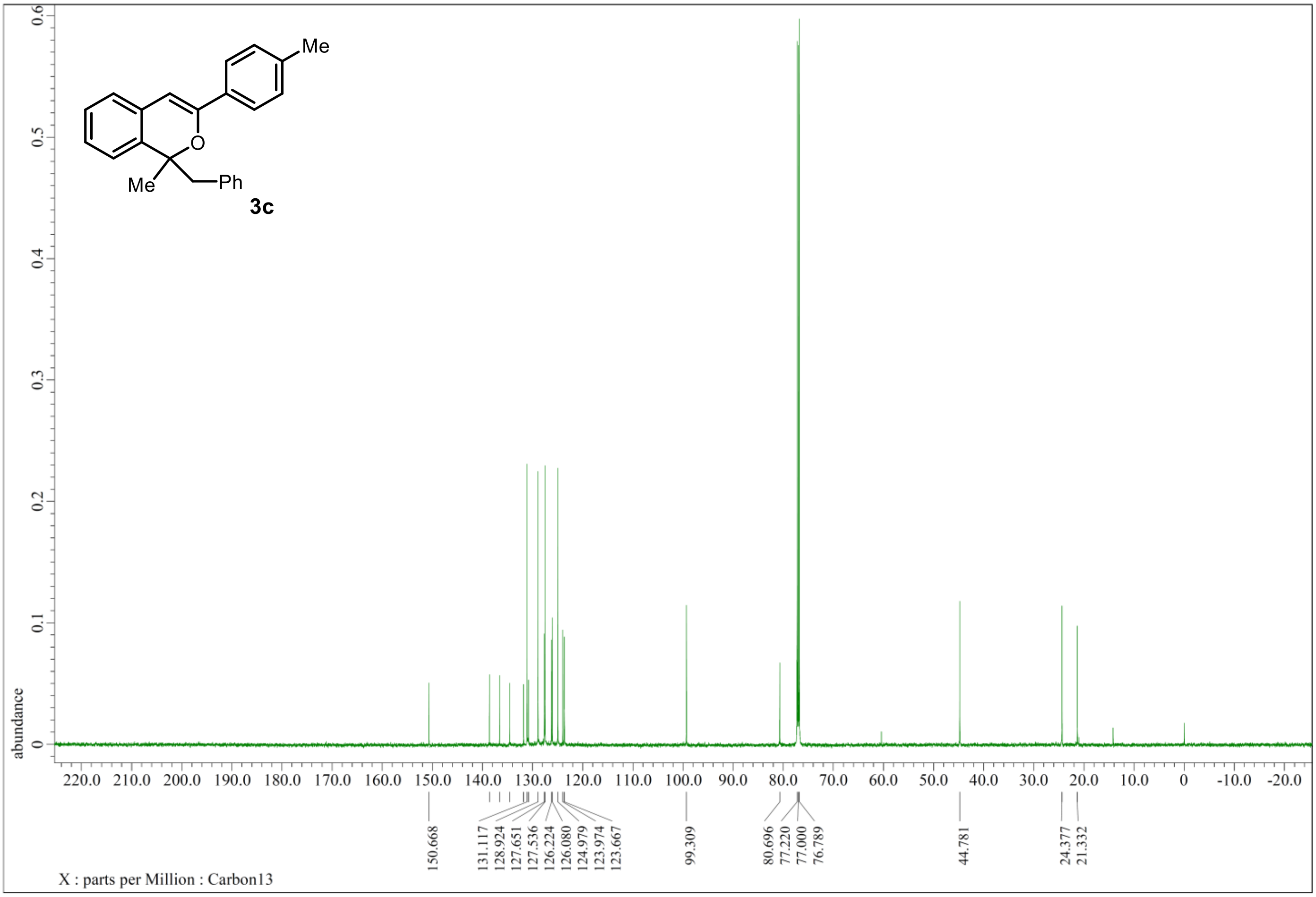

^{1}H NMR (600 MHz, $CDCl_3$) and $^{13}C\{^1H\}$ NMR (151 MHz, $CDCl_3$) spectra of **3d**.

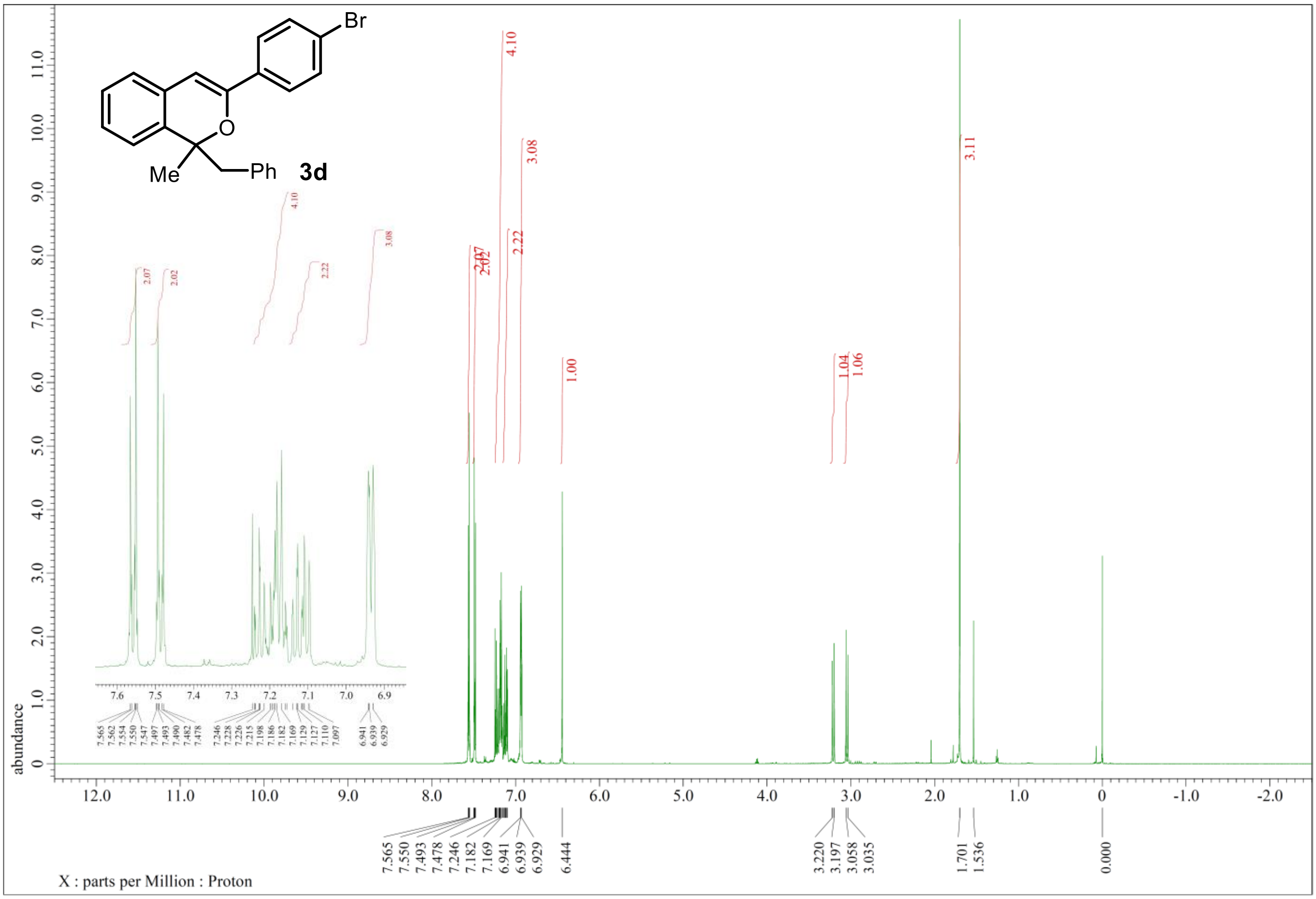

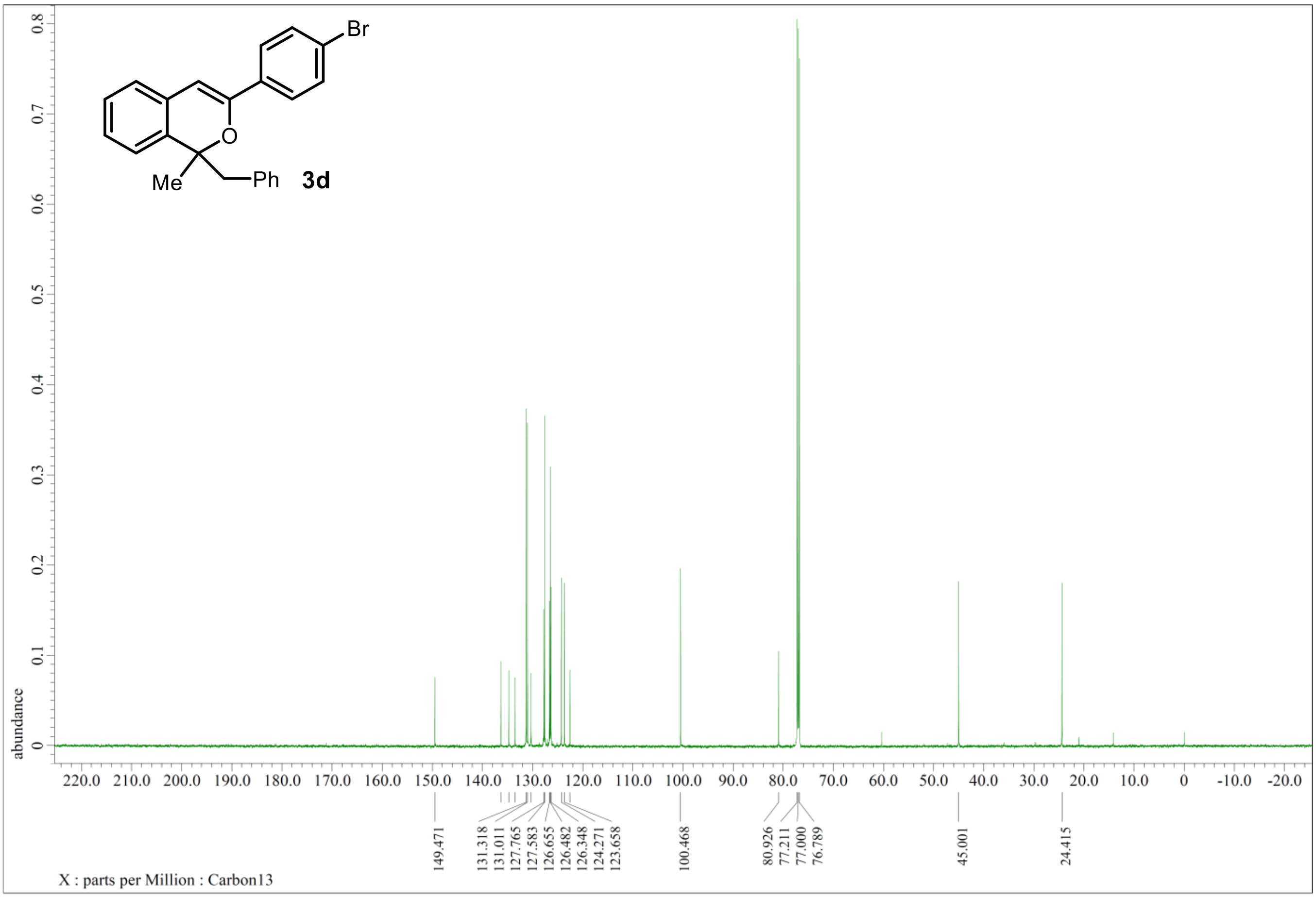

^1H NMR (600 MHz, $CDCl_3$) and $^{13}C\{^1H\}$ NMR (151 MHz, $CDCl_3$) spectra of **3e**.

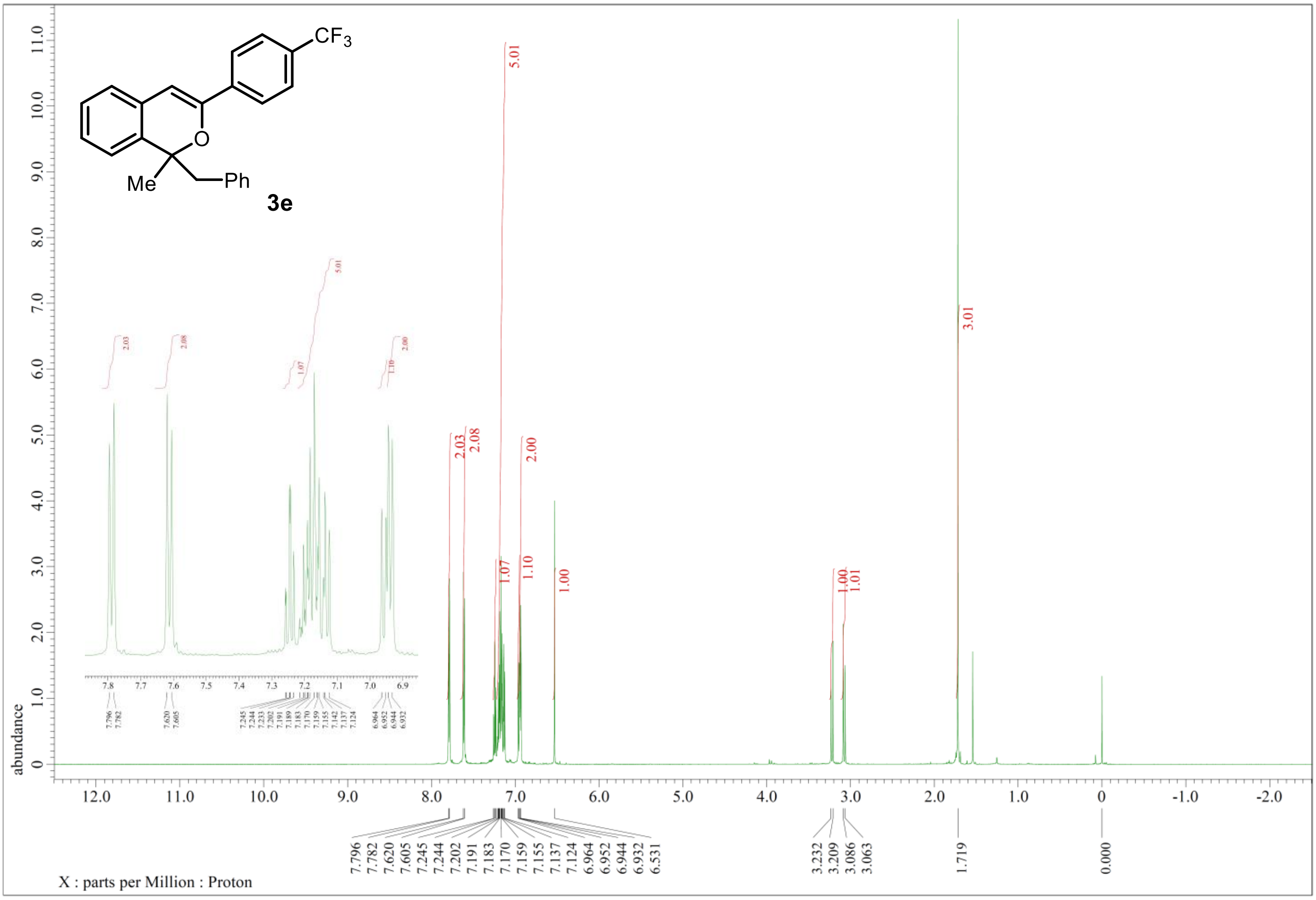

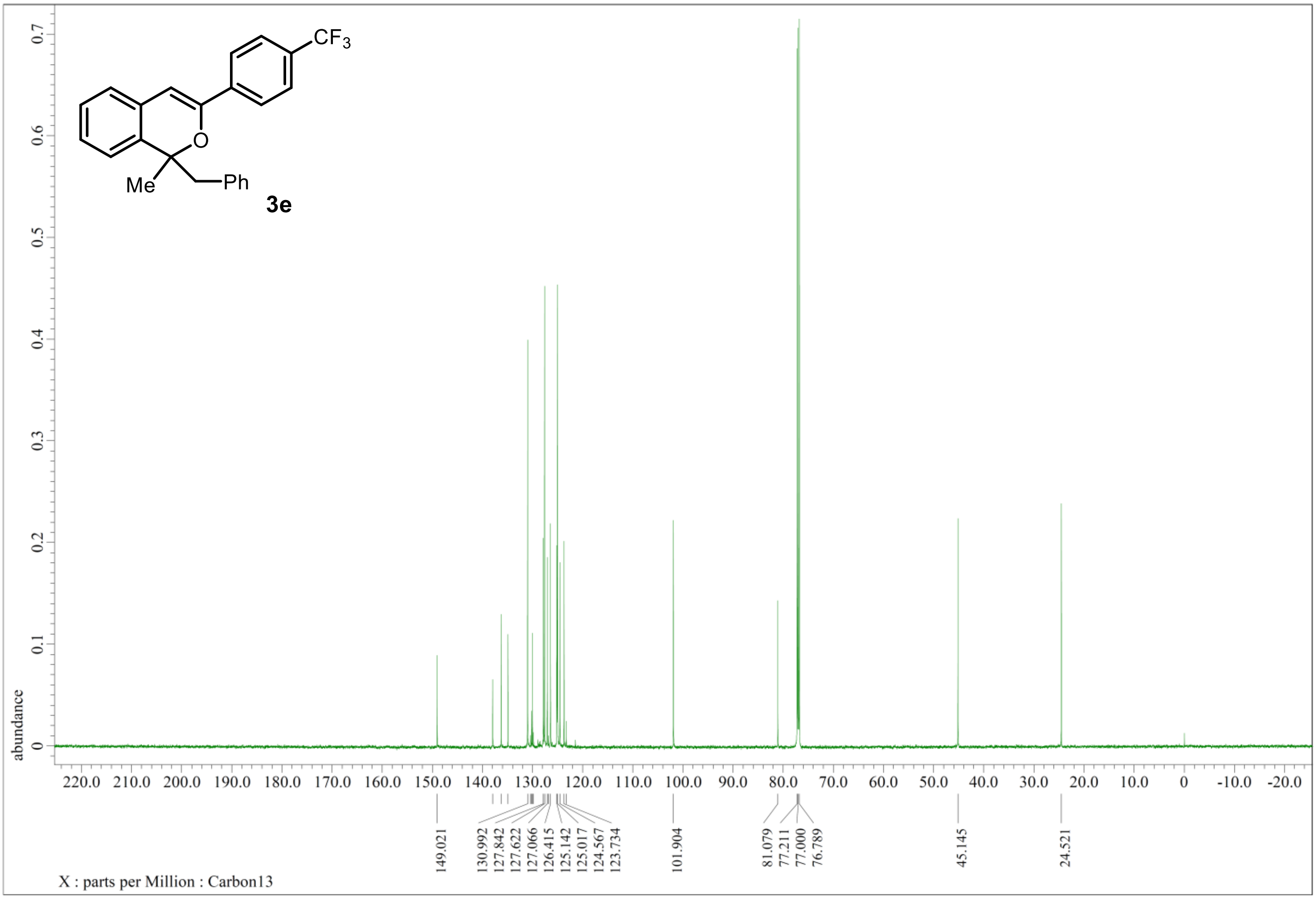

^{1}H NMR (600 MHz, $CDCl_3$) and $^{13}C\{^{1}H\}$ NMR (151 MHz, $CDCl_3$) spectra of **3f**.

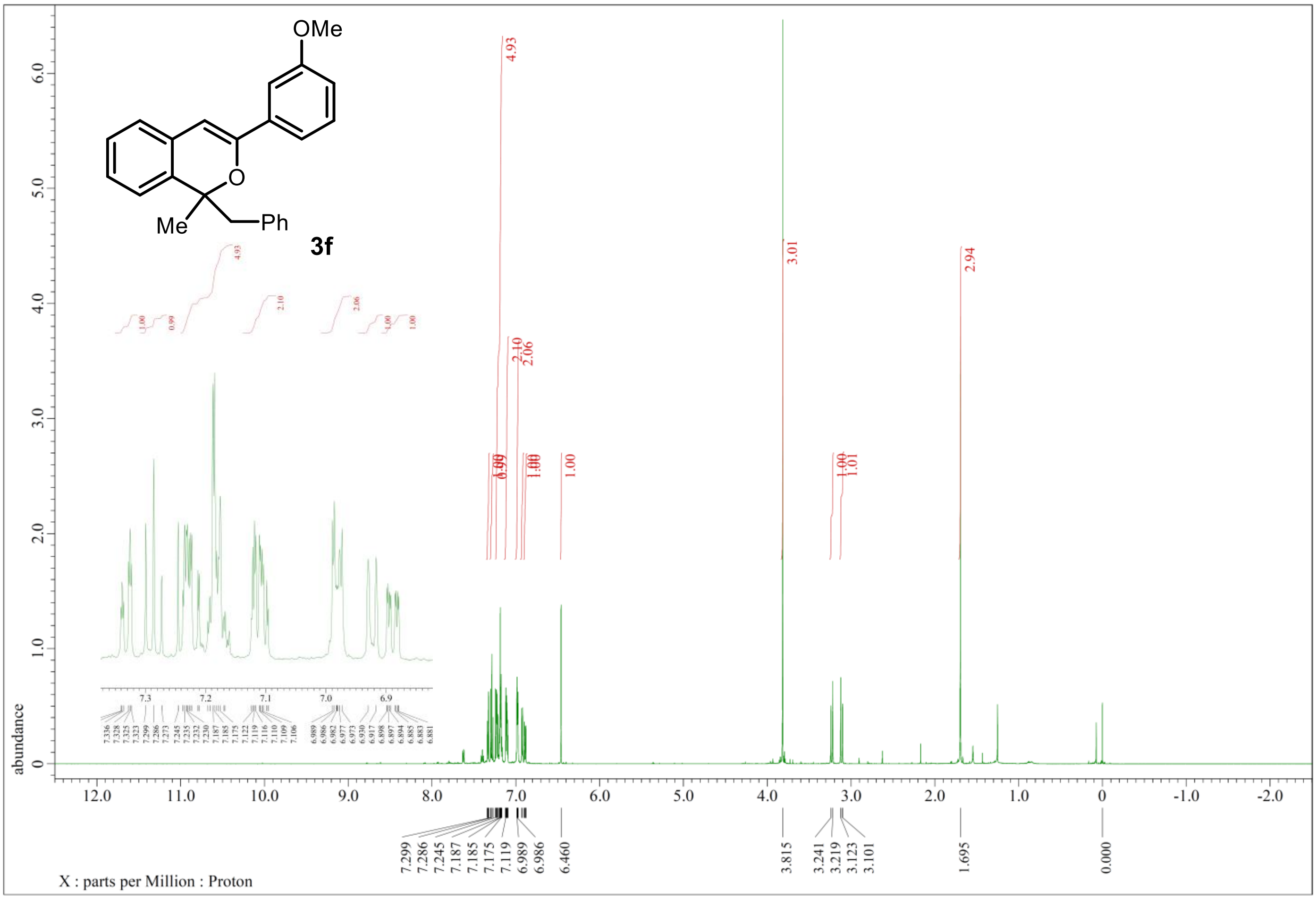

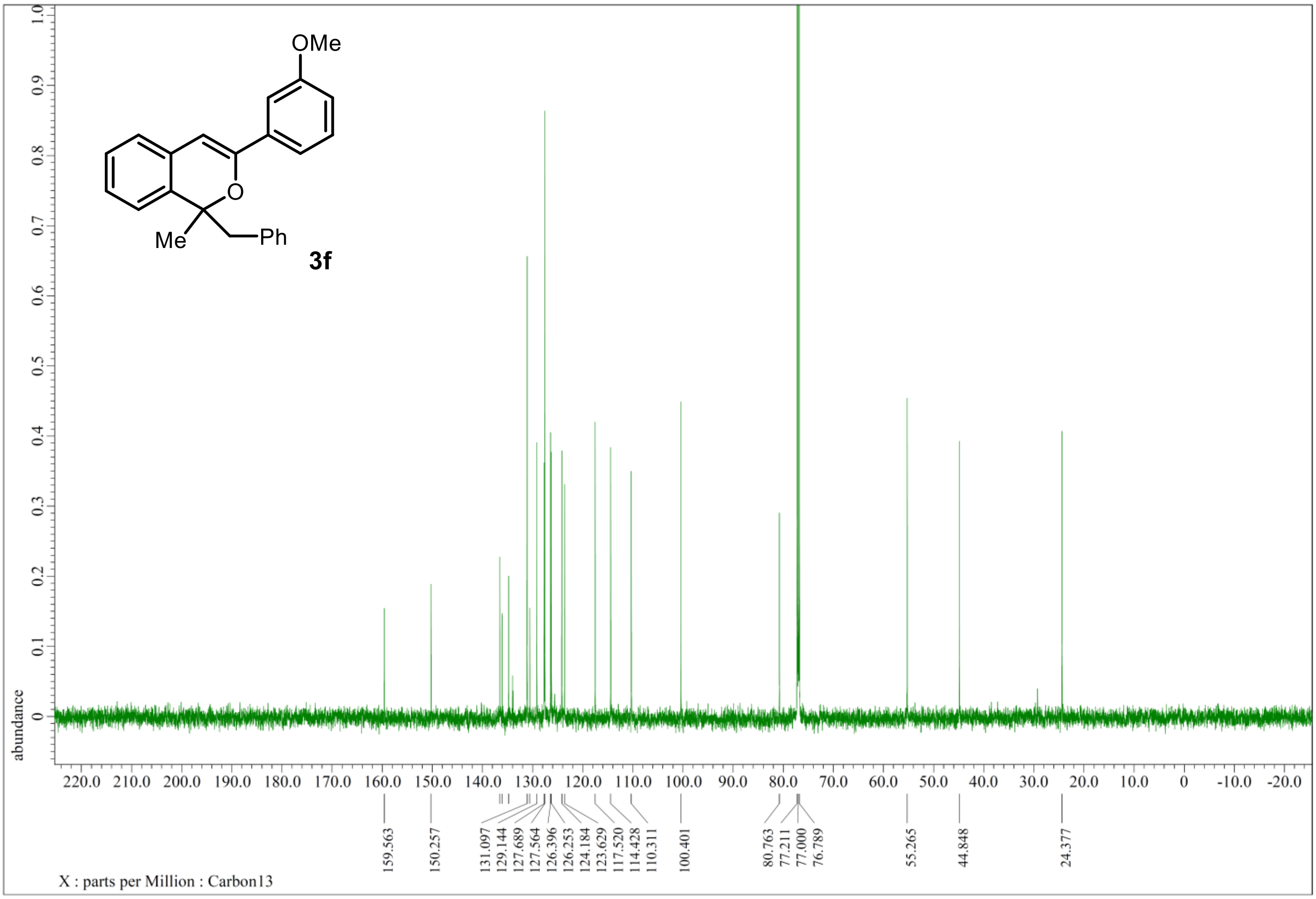

^{1}H NMR (600 MHz, $CDCl_3$) and $^{13}C\{^{1}H\}$ NMR (151 MHz, $CDCl_3$) spectra of **3g**.

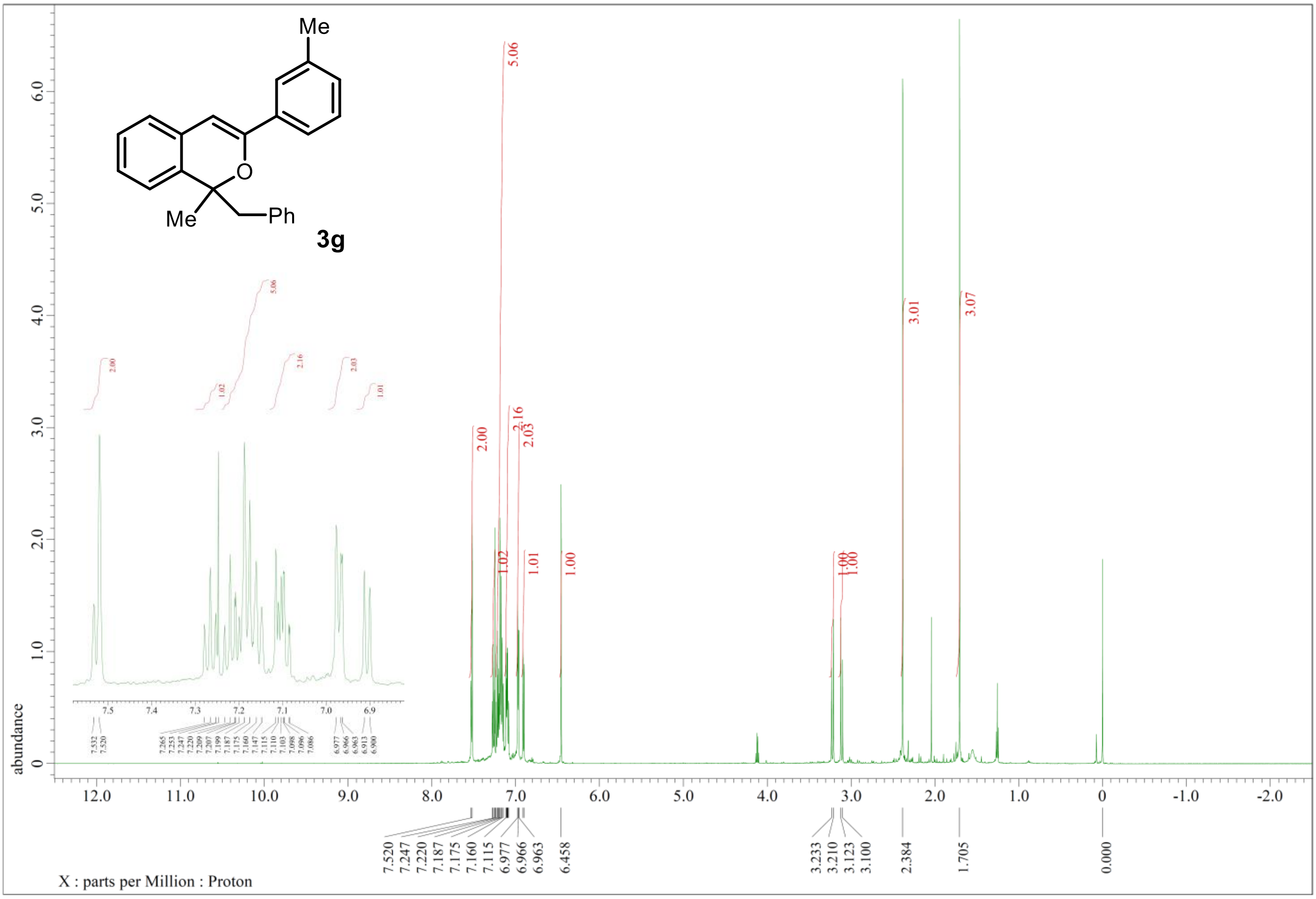

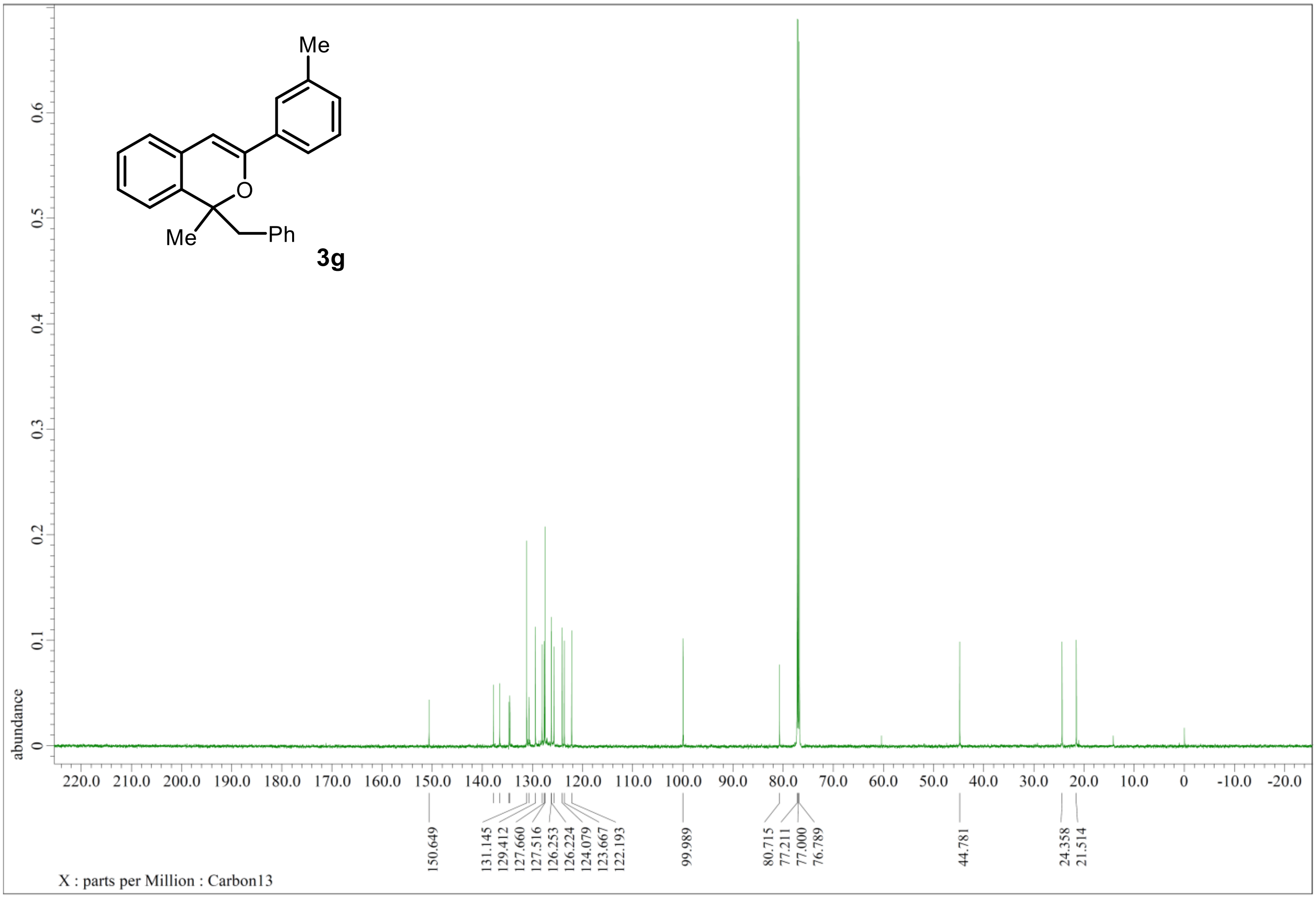

^{1}H NMR (600 MHz, $CDCl_3$) and $^{13}C\{^{1}H\}$ NMR (151 MHz, $CDCl_3$) spectra of **3h**.

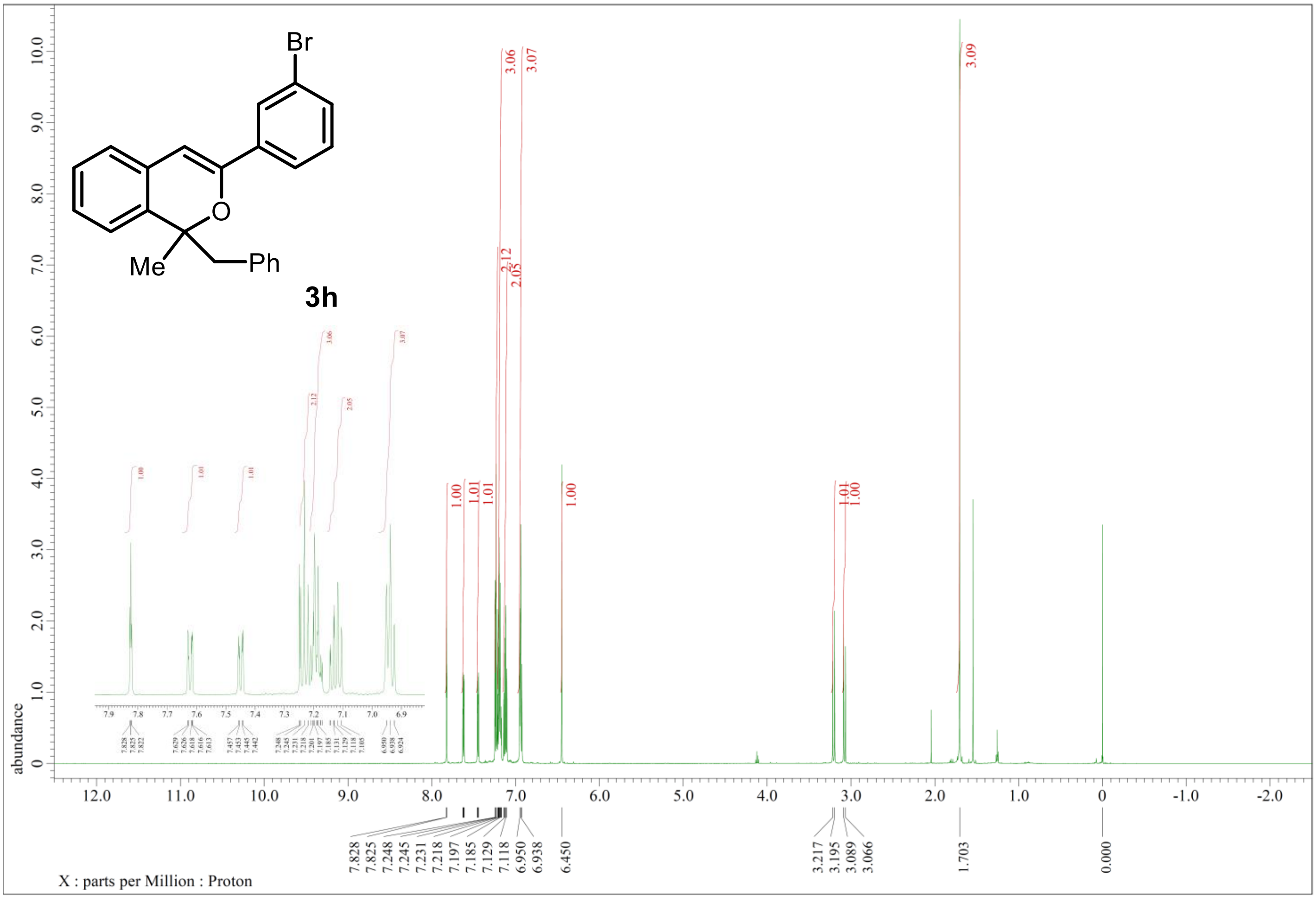

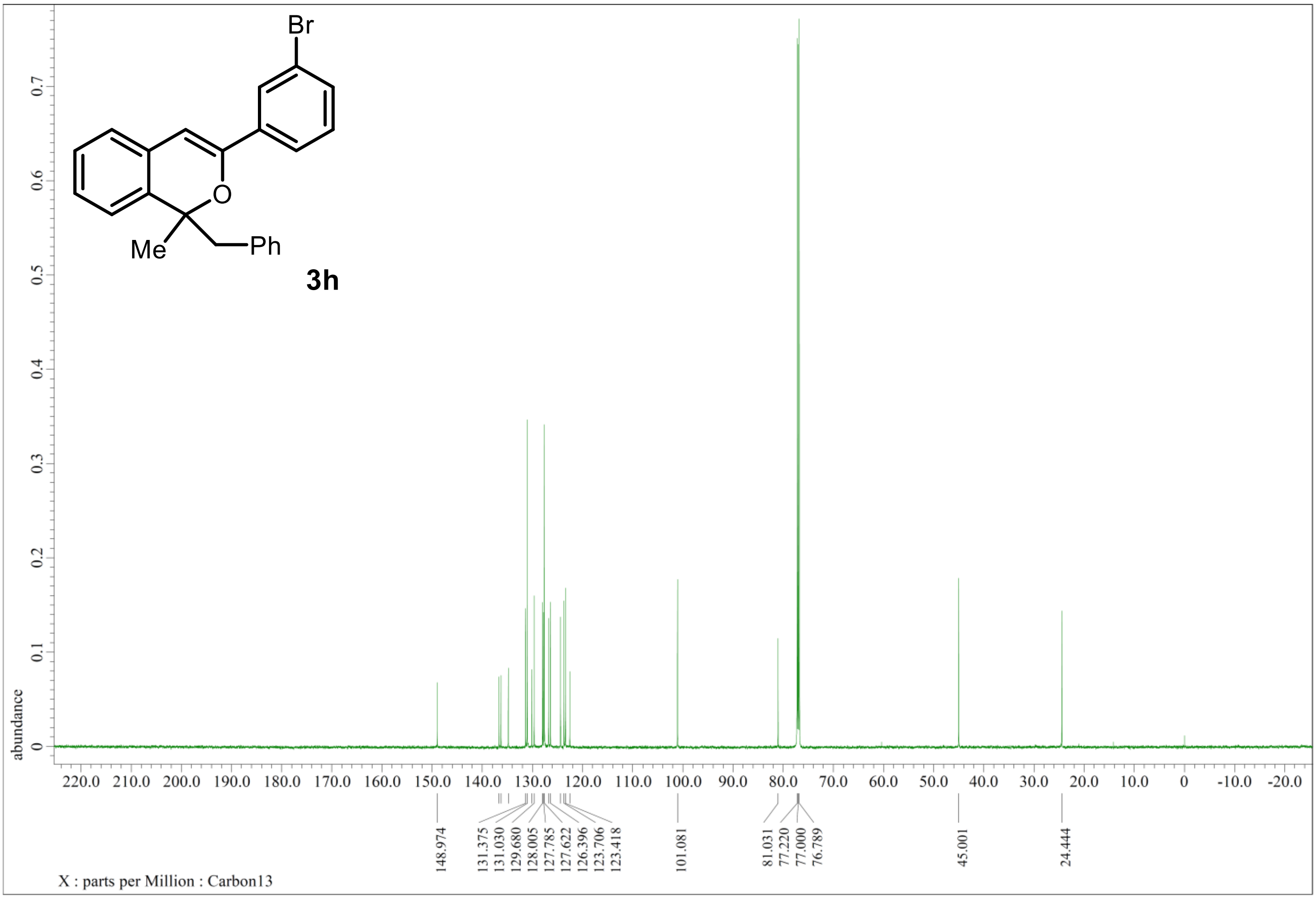

^{1}H NMR (600 MHz, $CDCl_3$) and $^{13}C\{^{1}H\}$ NMR (151 MHz, $CDCl_3$) spectra of **3i**.

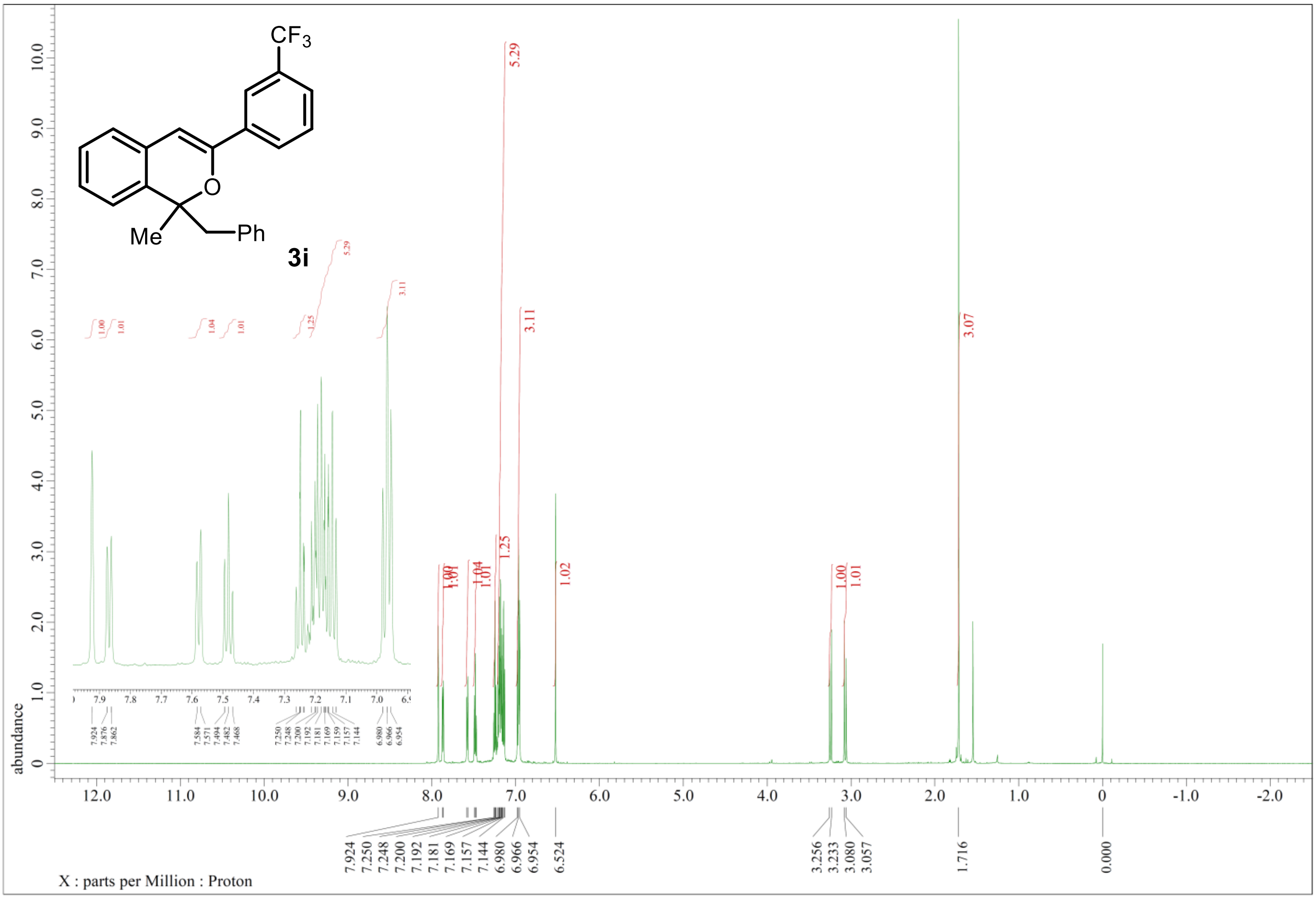

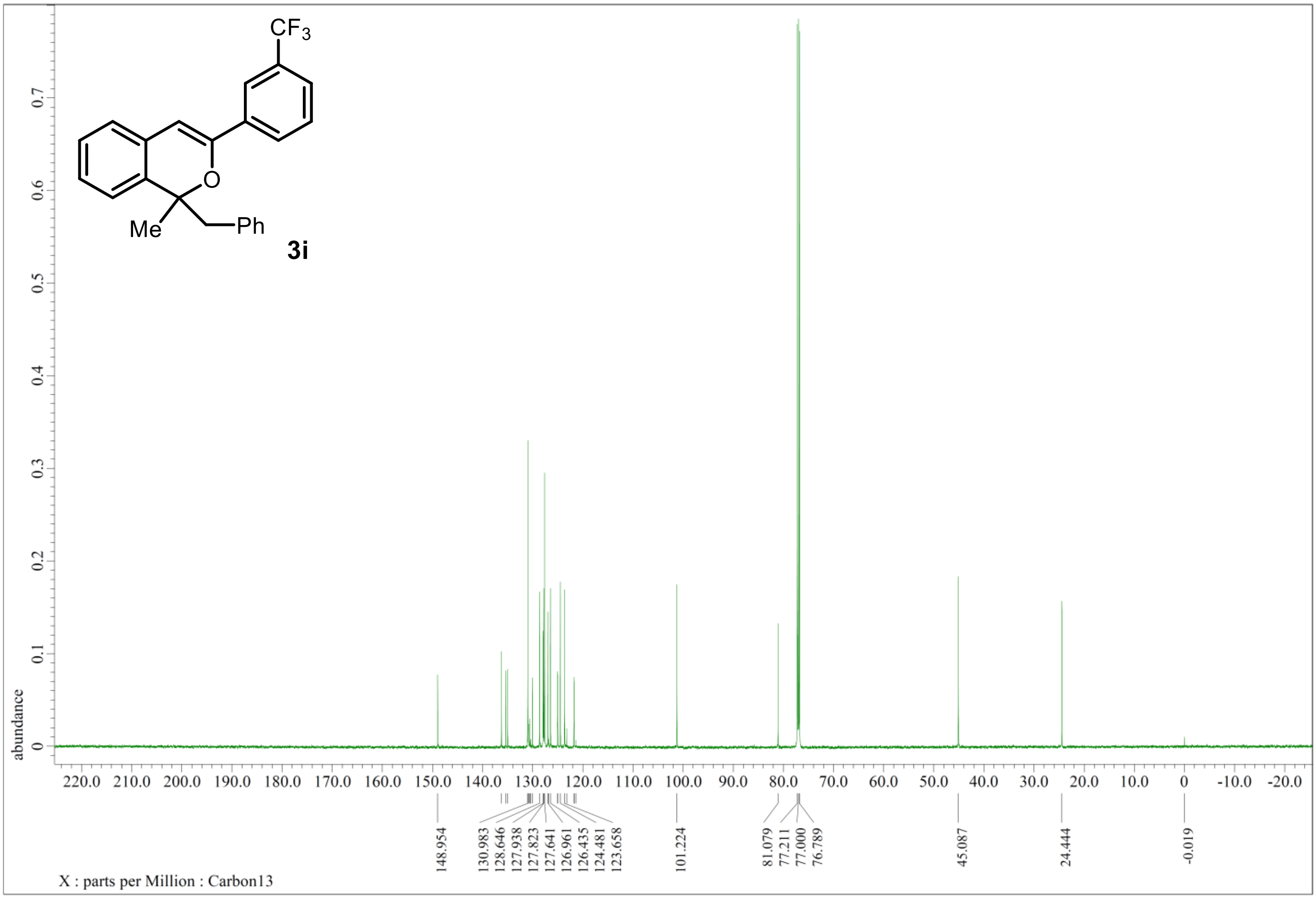

^1H NMR (600 MHz, $CDCl_3$) and $^{13}C\{^1H\}$ NMR (151 MHz, $CDCl_3$) spectra of **3j**.

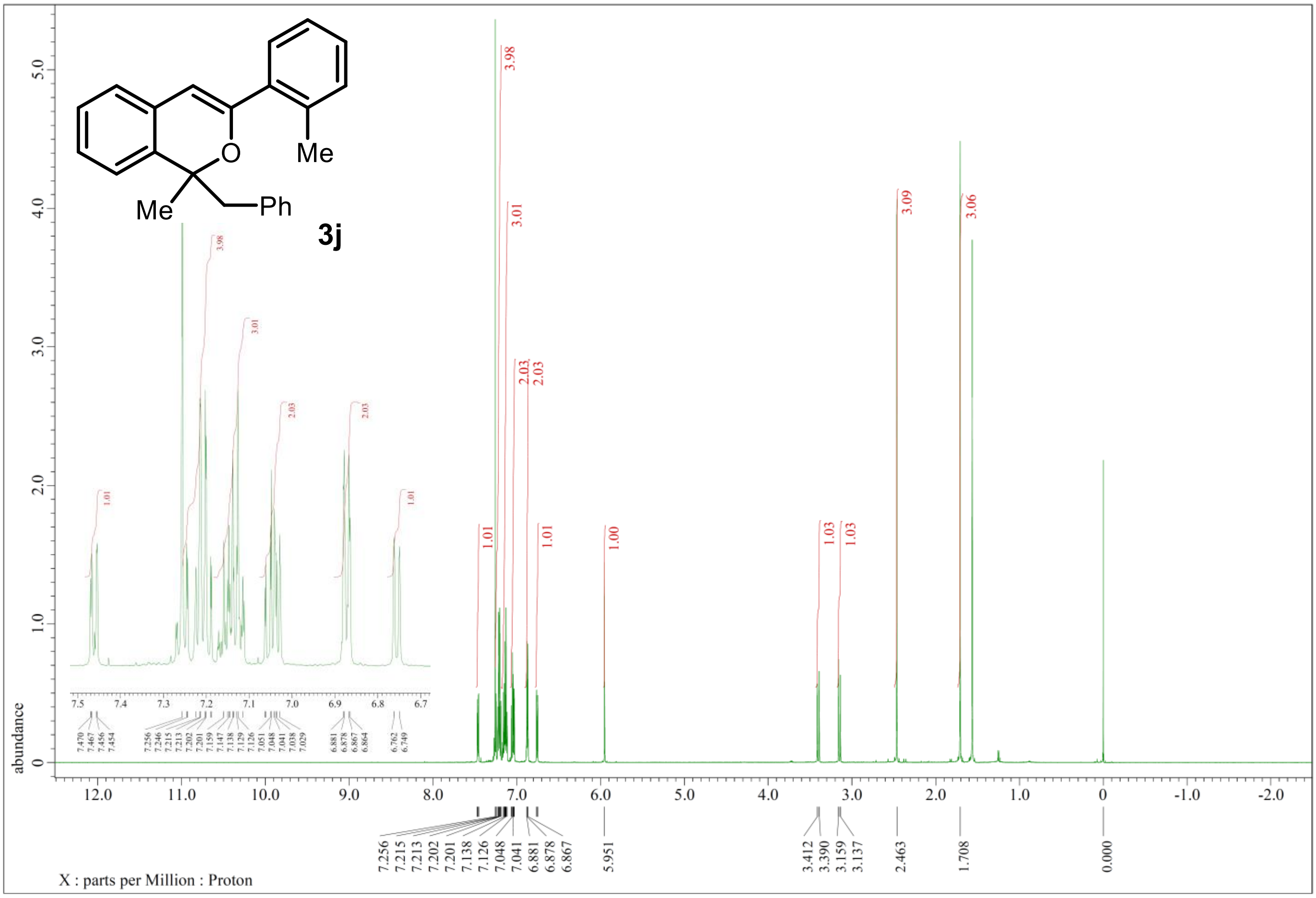

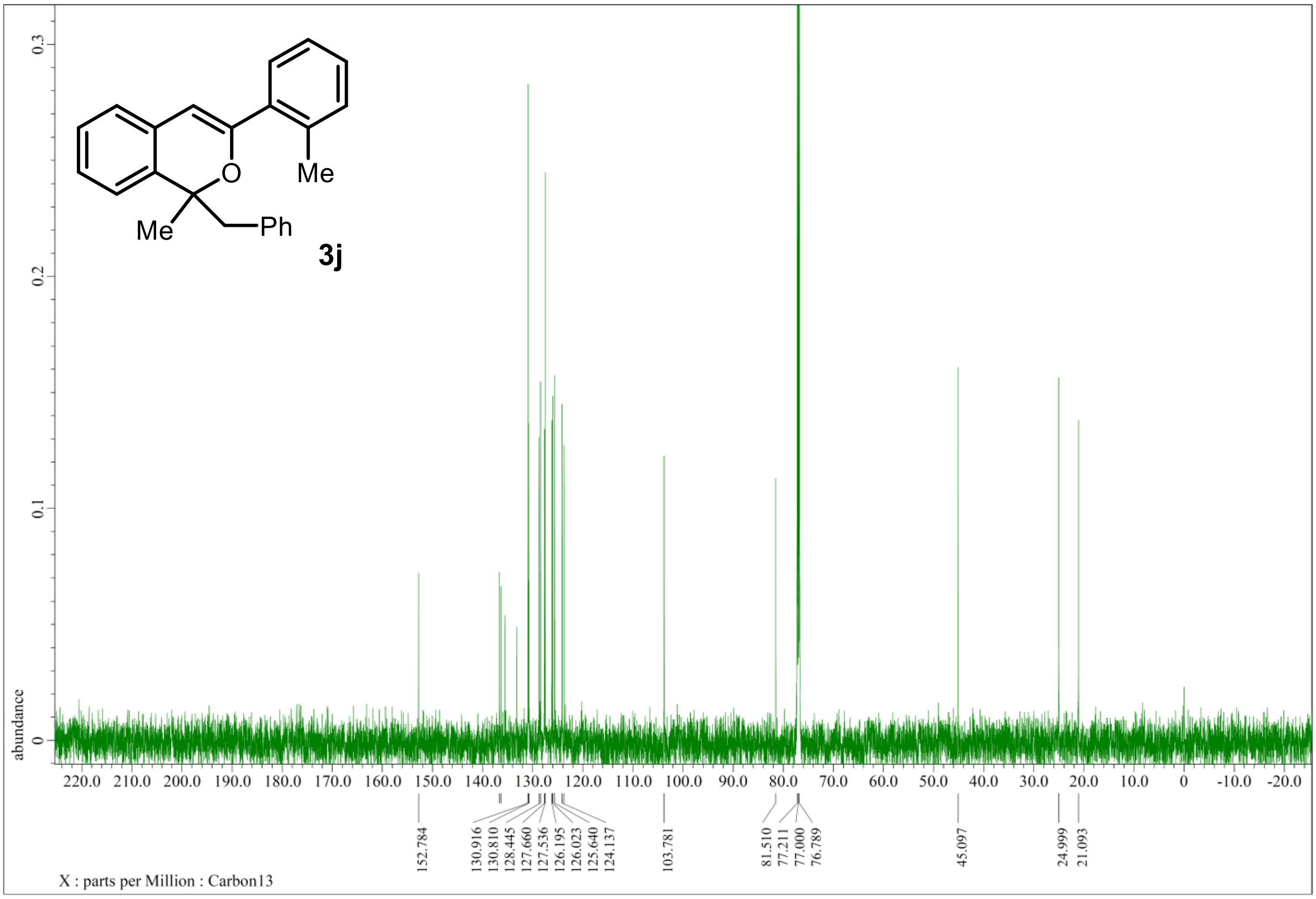

^{1}H NMR (600 MHz, $CDCl_3$) and $^{13}C\{^{1}H\}$ NMR (151 MHz, $CDCl_3$) spectra of **3k**.

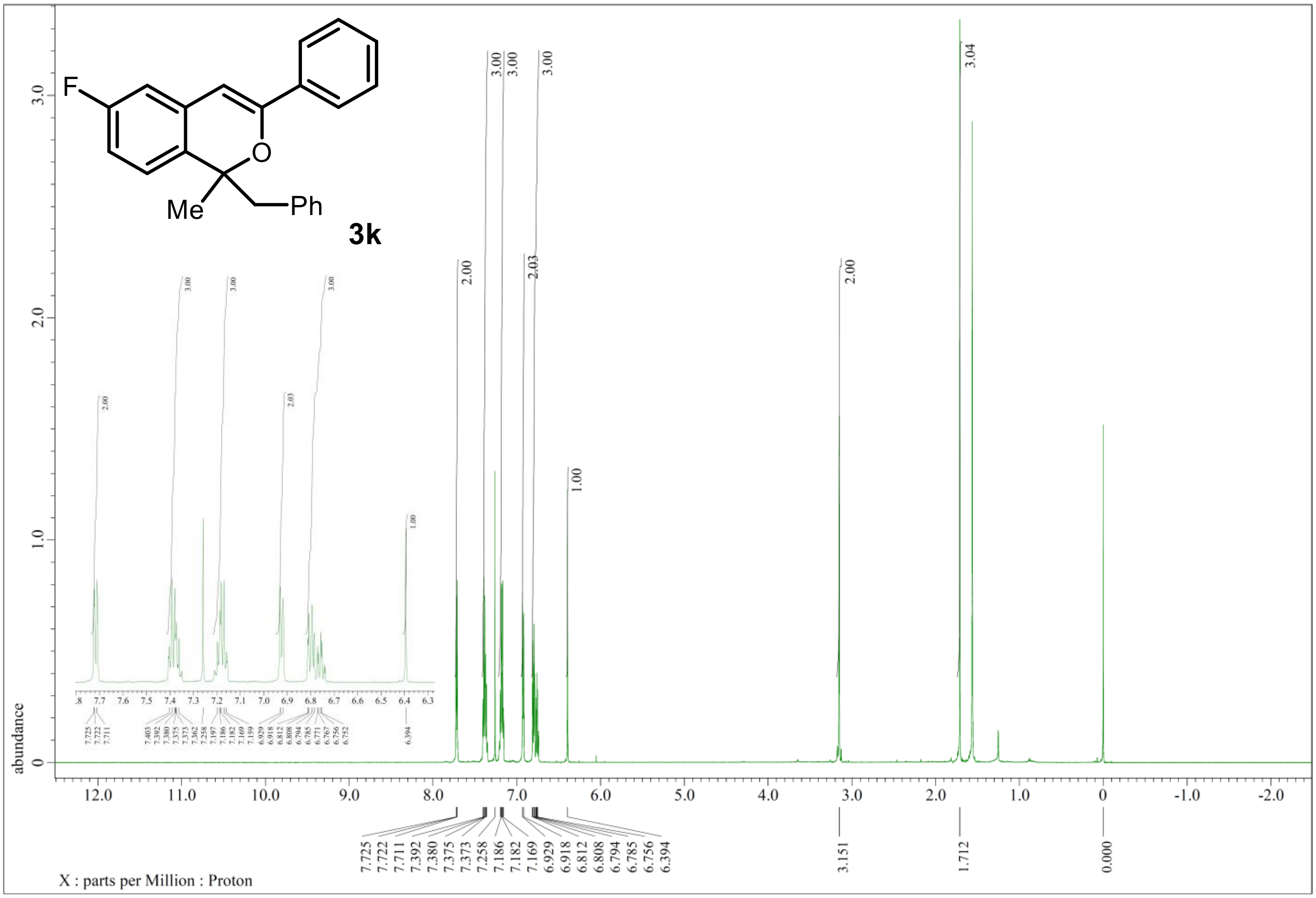

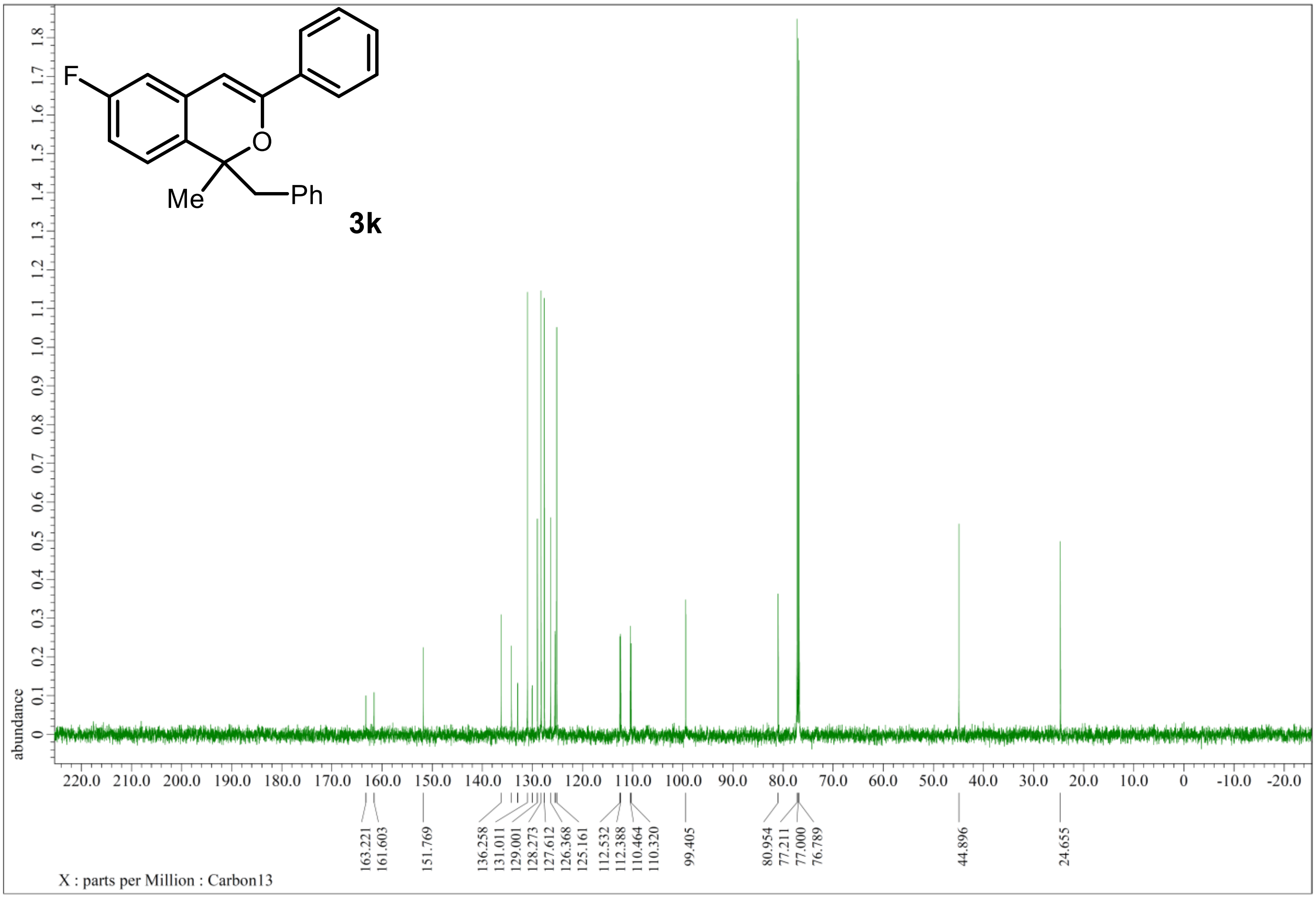

^1H NMR (600 MHz, $CDCl_3$) and $^{13}C\{^1H\}$ NMR (151 MHz, $CDCl_3$) spectra of **3l**.

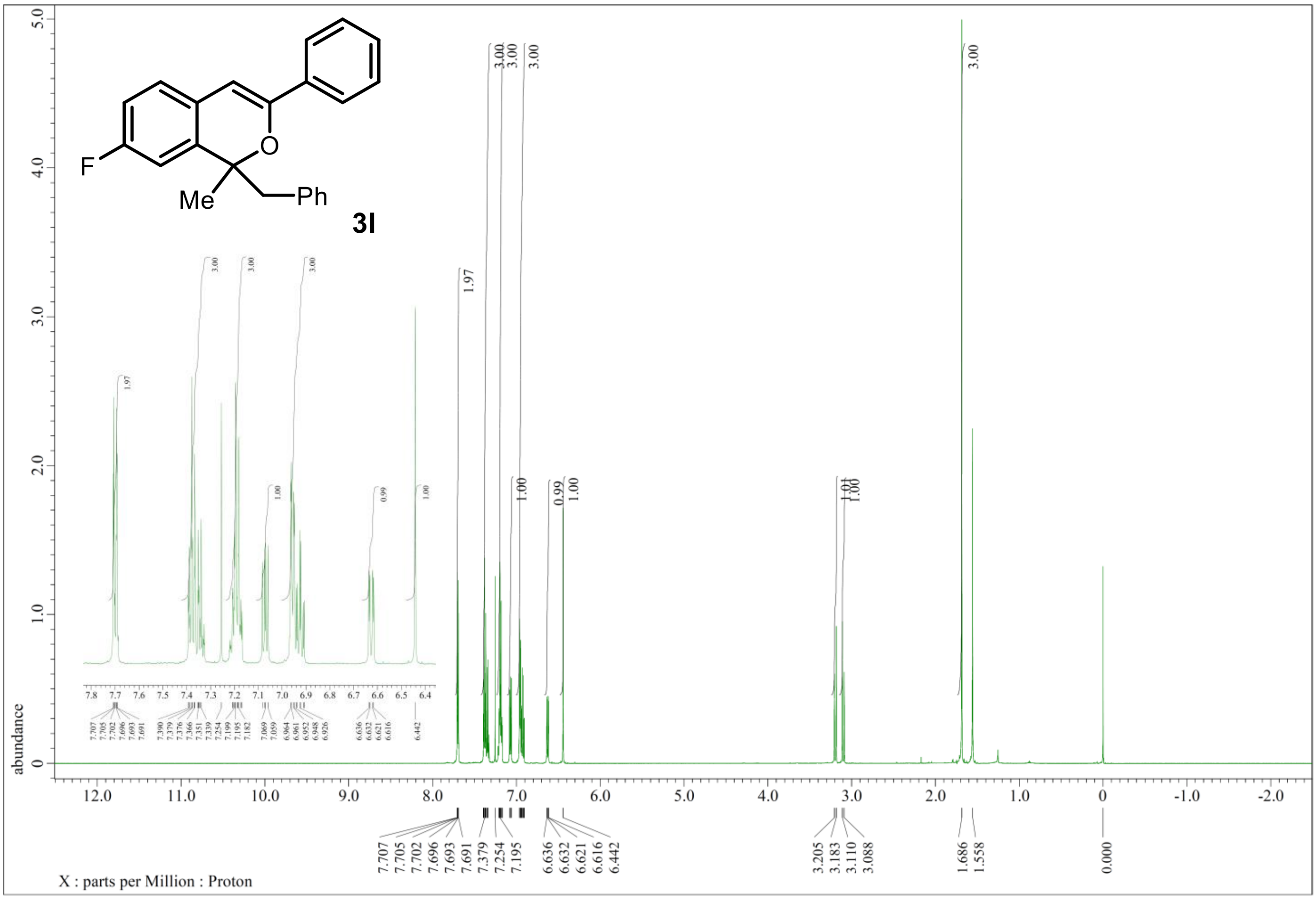

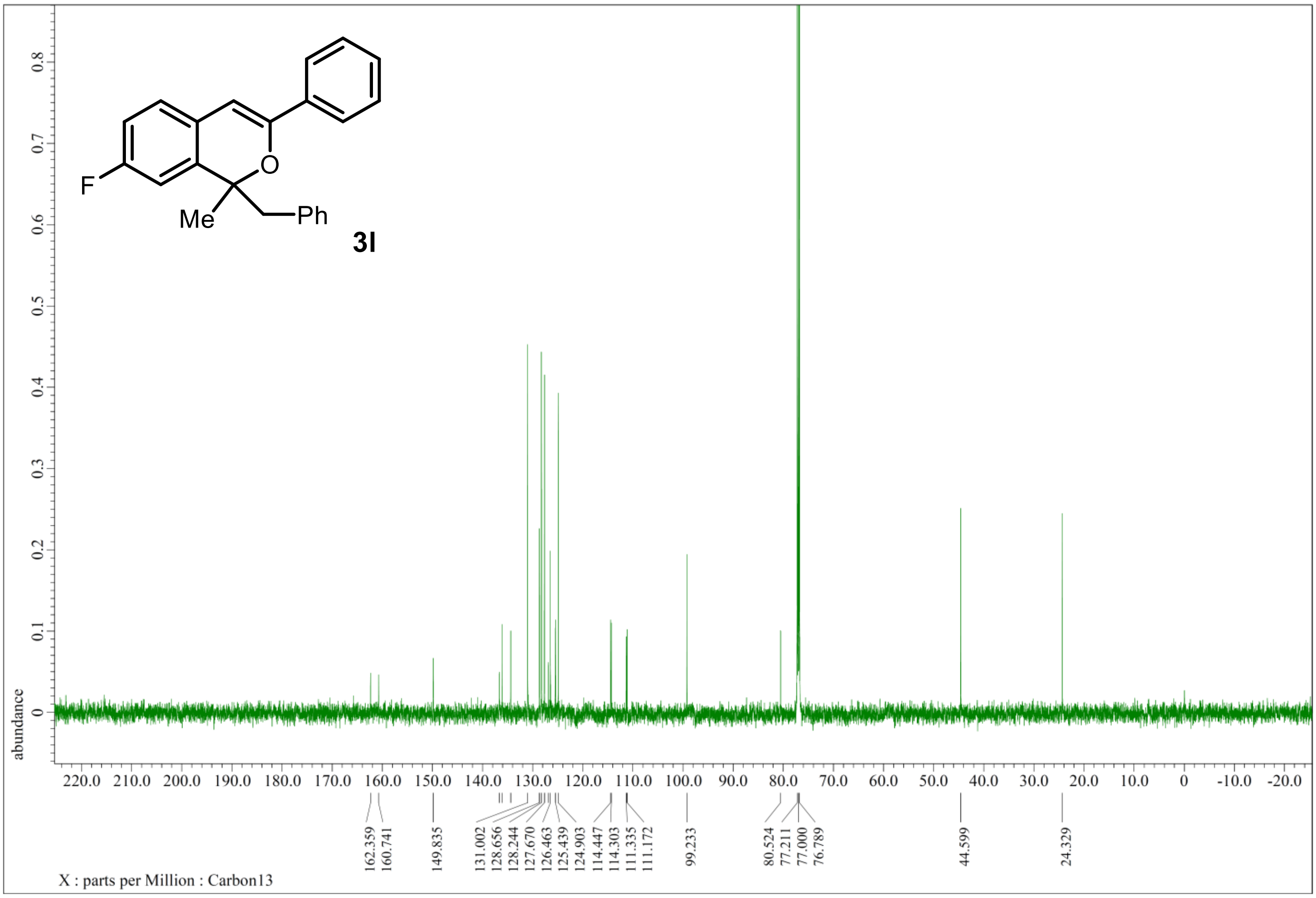

^{1}H NMR (600 MHz, $CDCl_3$) and $^{13}C\{^1H\}$ NMR (151 MHz, $CDCl_3$) spectra of **3m**.

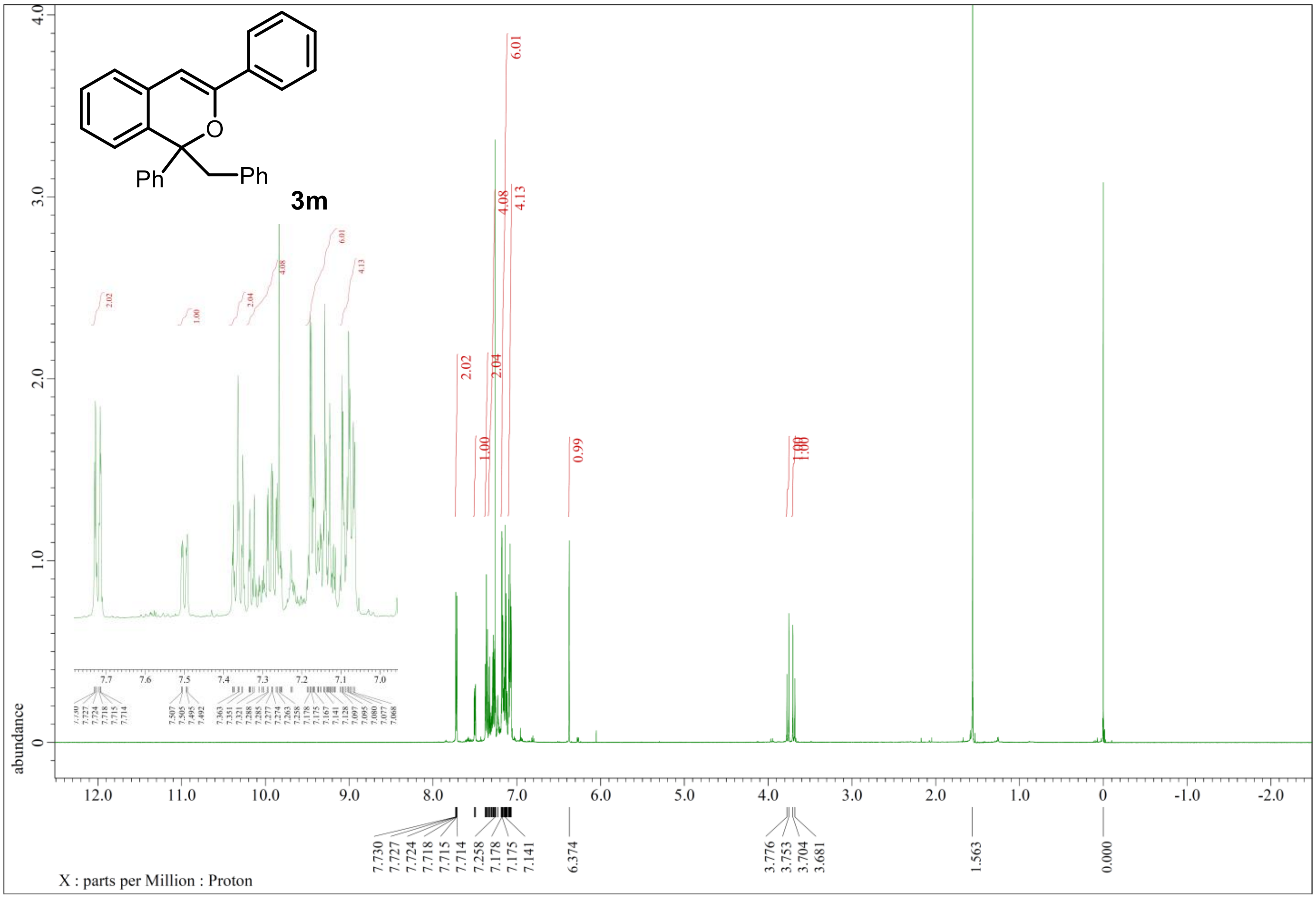

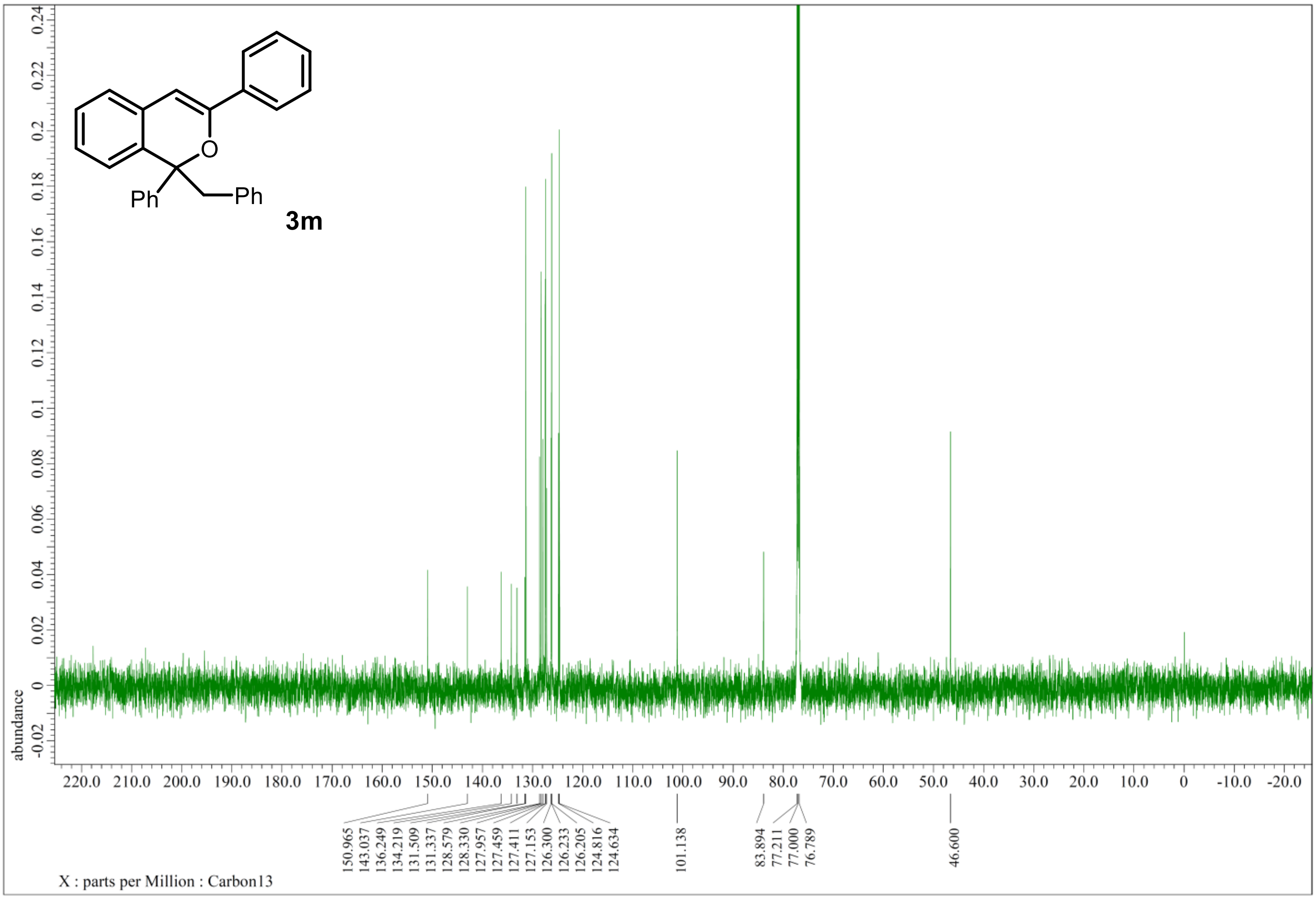

^{1}H NMR (600 MHz, $CDCl_3$) and $^{13}C\{^1H\}$ NMR (151 MHz, $CDCl_3$) spectra of **3n**.

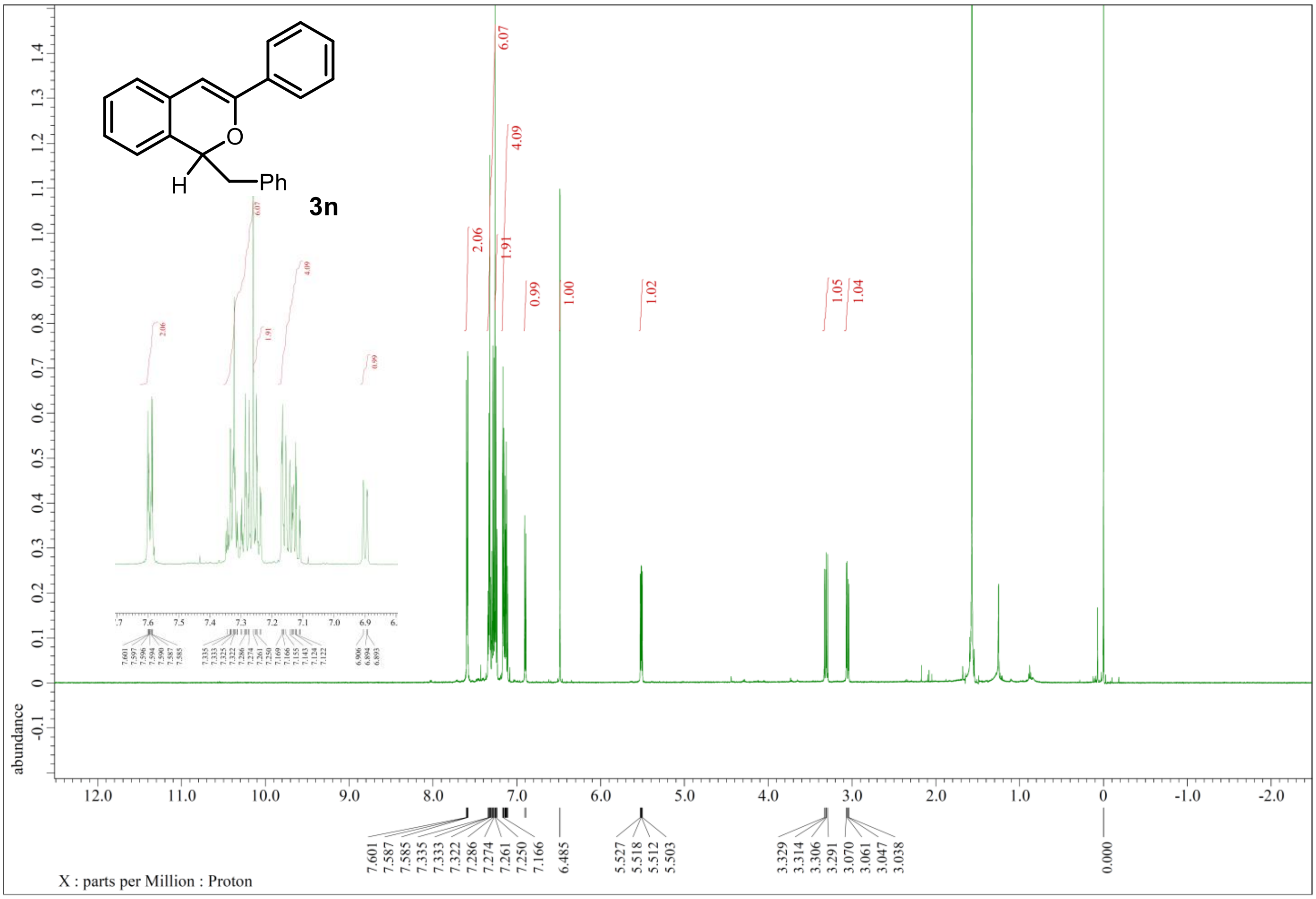

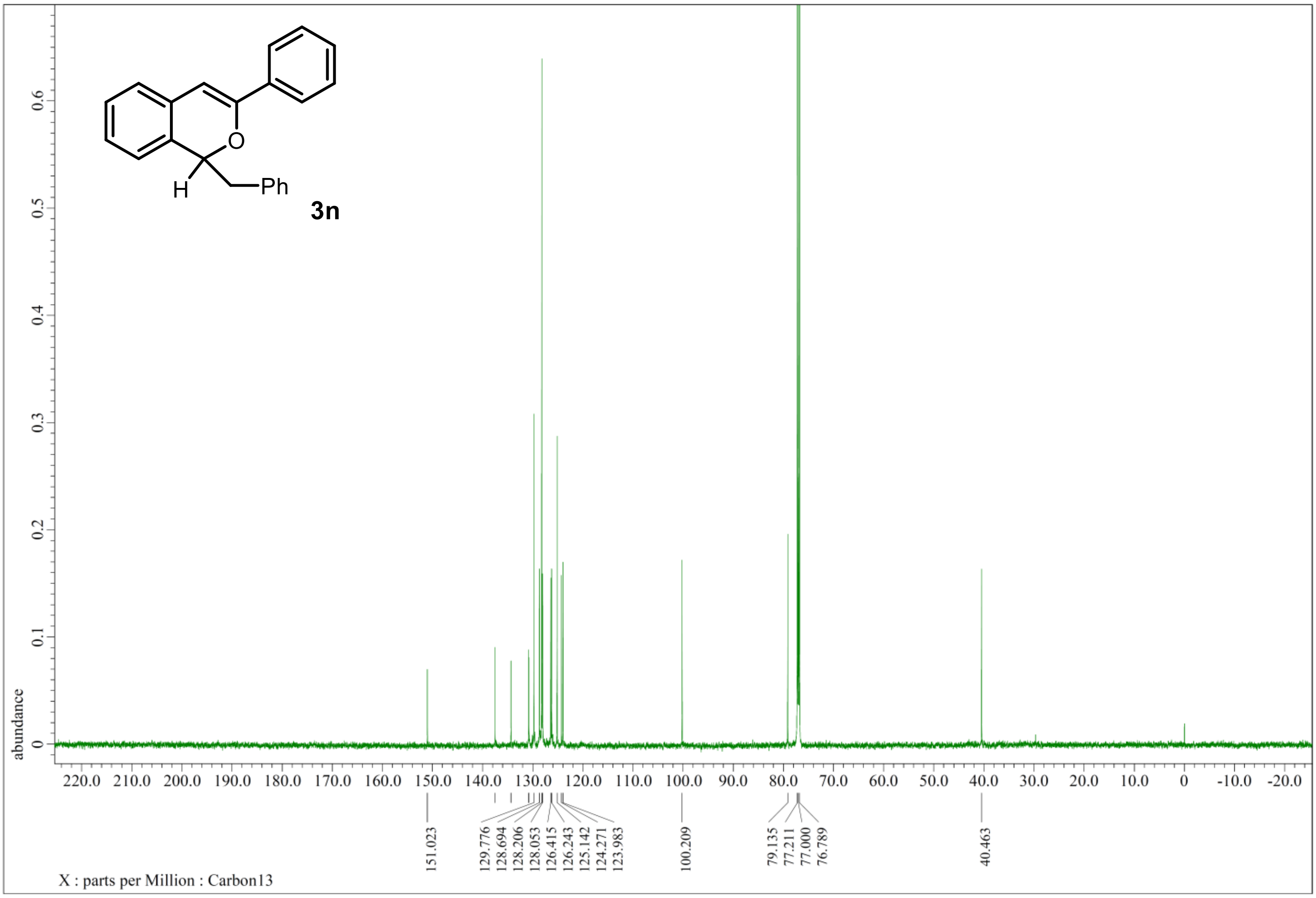

^1H NMR (600 MHz, $CDCl_3$) and $^{13}C\{^1H\}$ NMR (151 MHz, $CDCl_3$) spectra of **3o**.

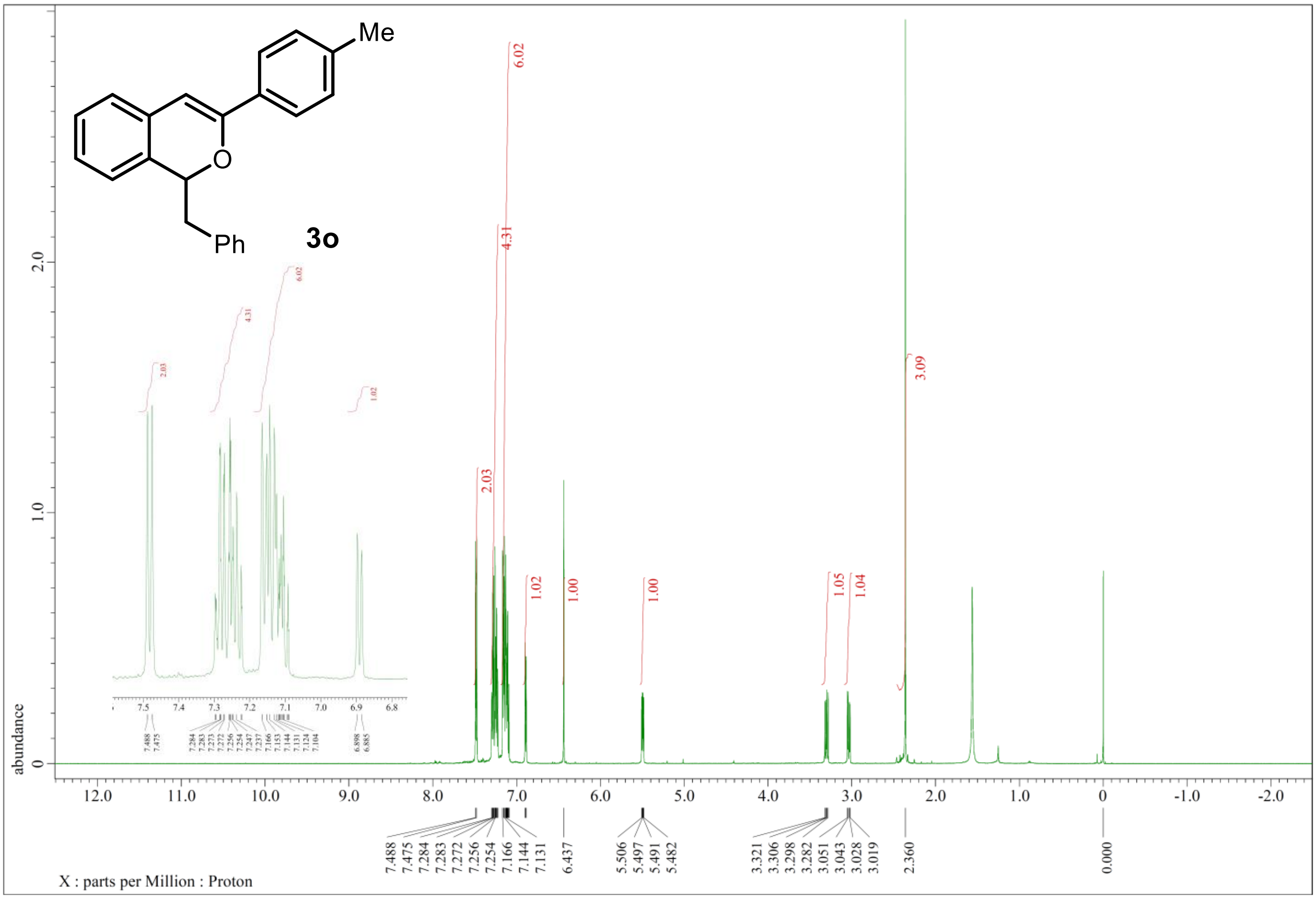

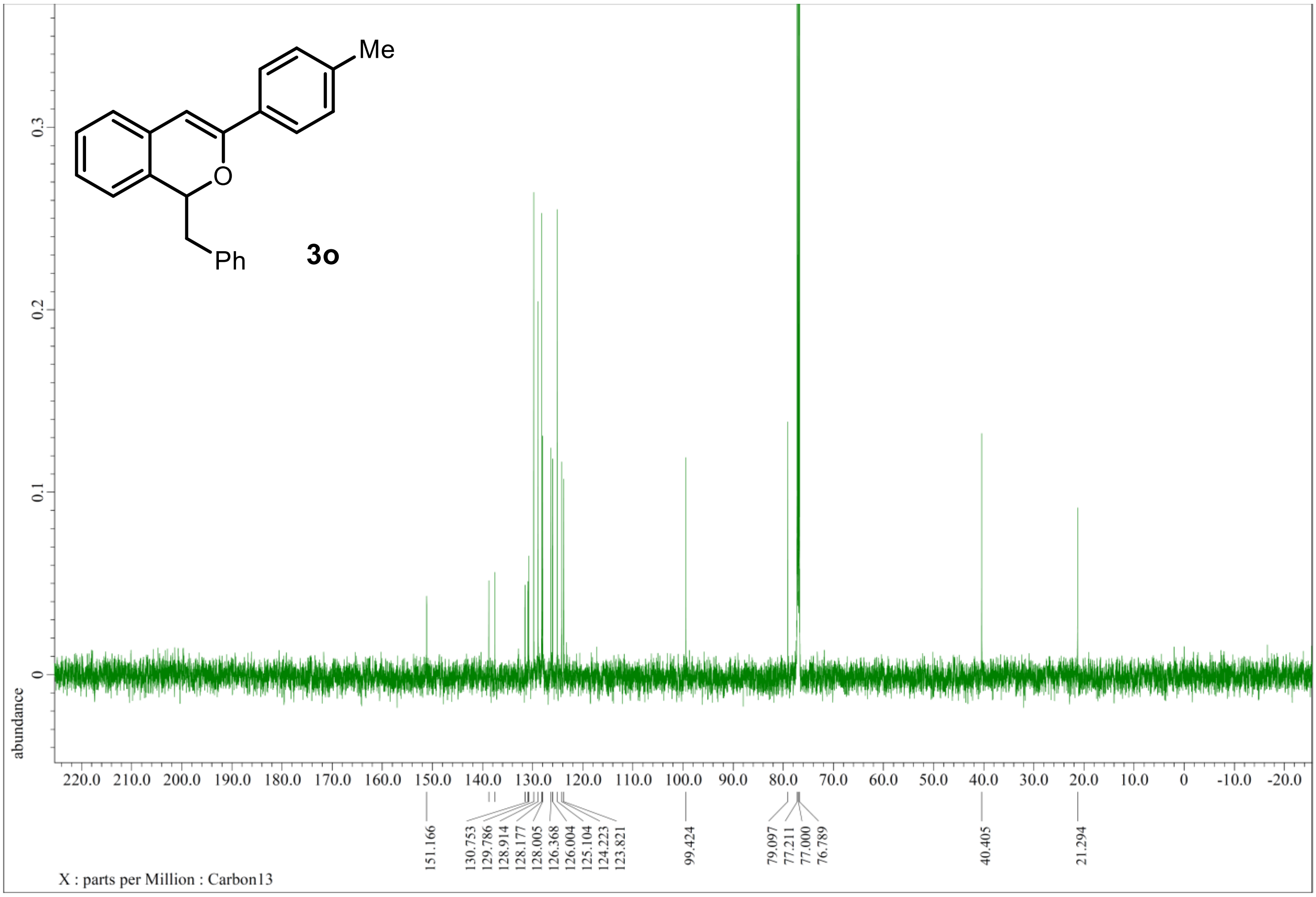

^{1}H NMR (600 MHz, $CDCl_3$) and $^{13}C\{^{1}H\}$ NMR (151 MHz, $CDCl_3$) spectra of **3p**.

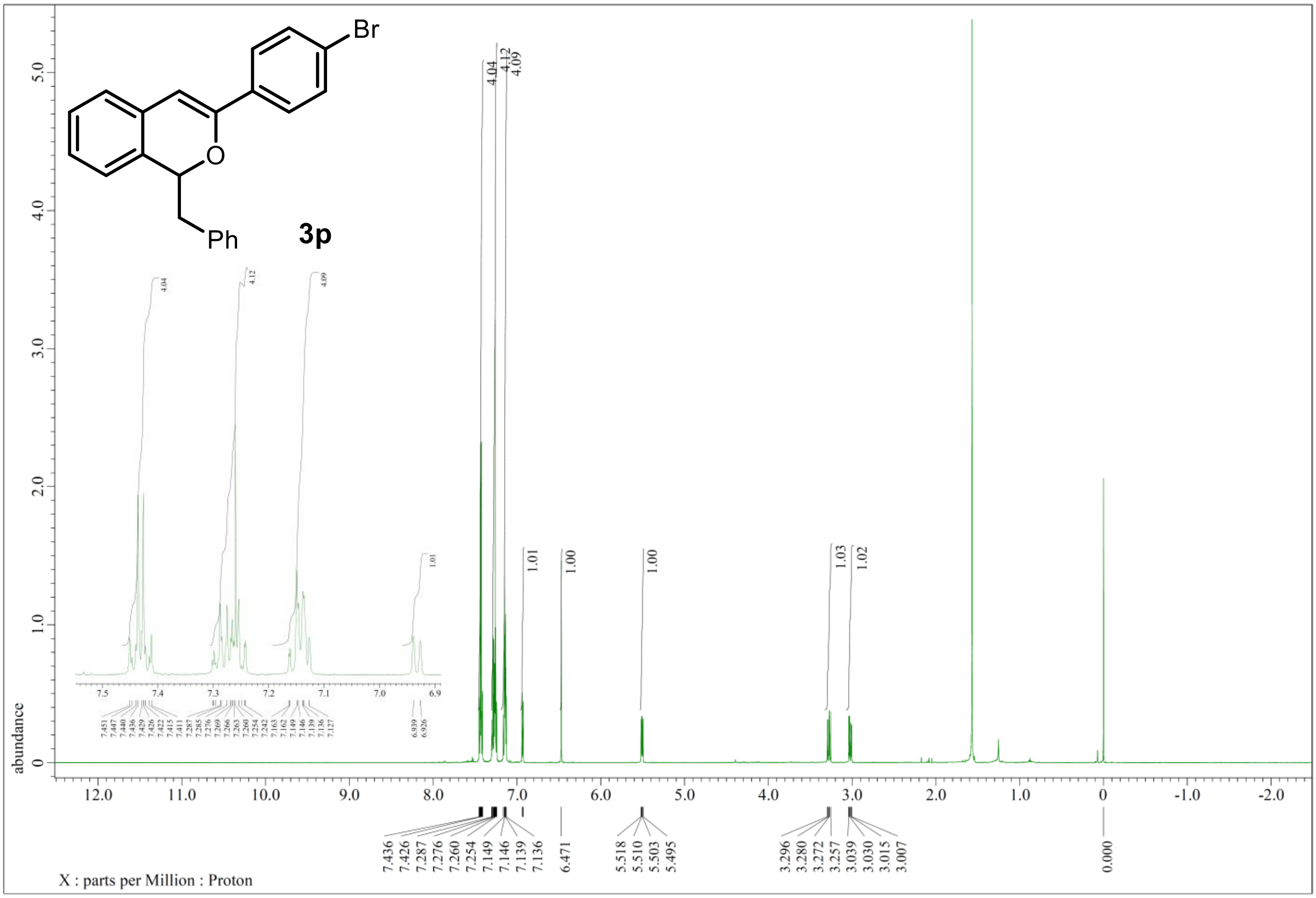

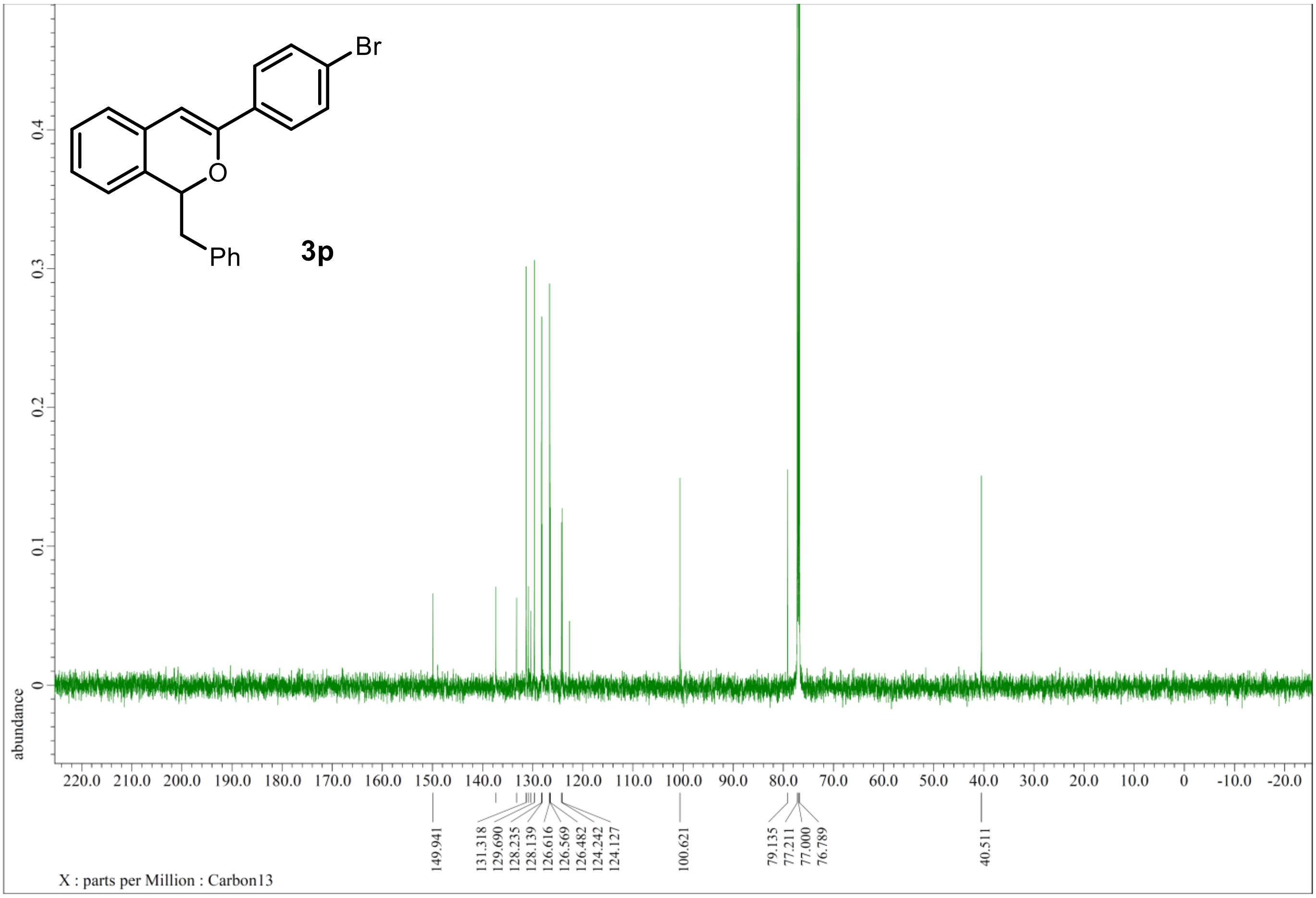

^{1}H NMR (600 MHz, $CDCl_3$) and $^{13}C\{^{1}H\}$ NMR (151 MHz, $CDCl_3$) spectra of **3q**.

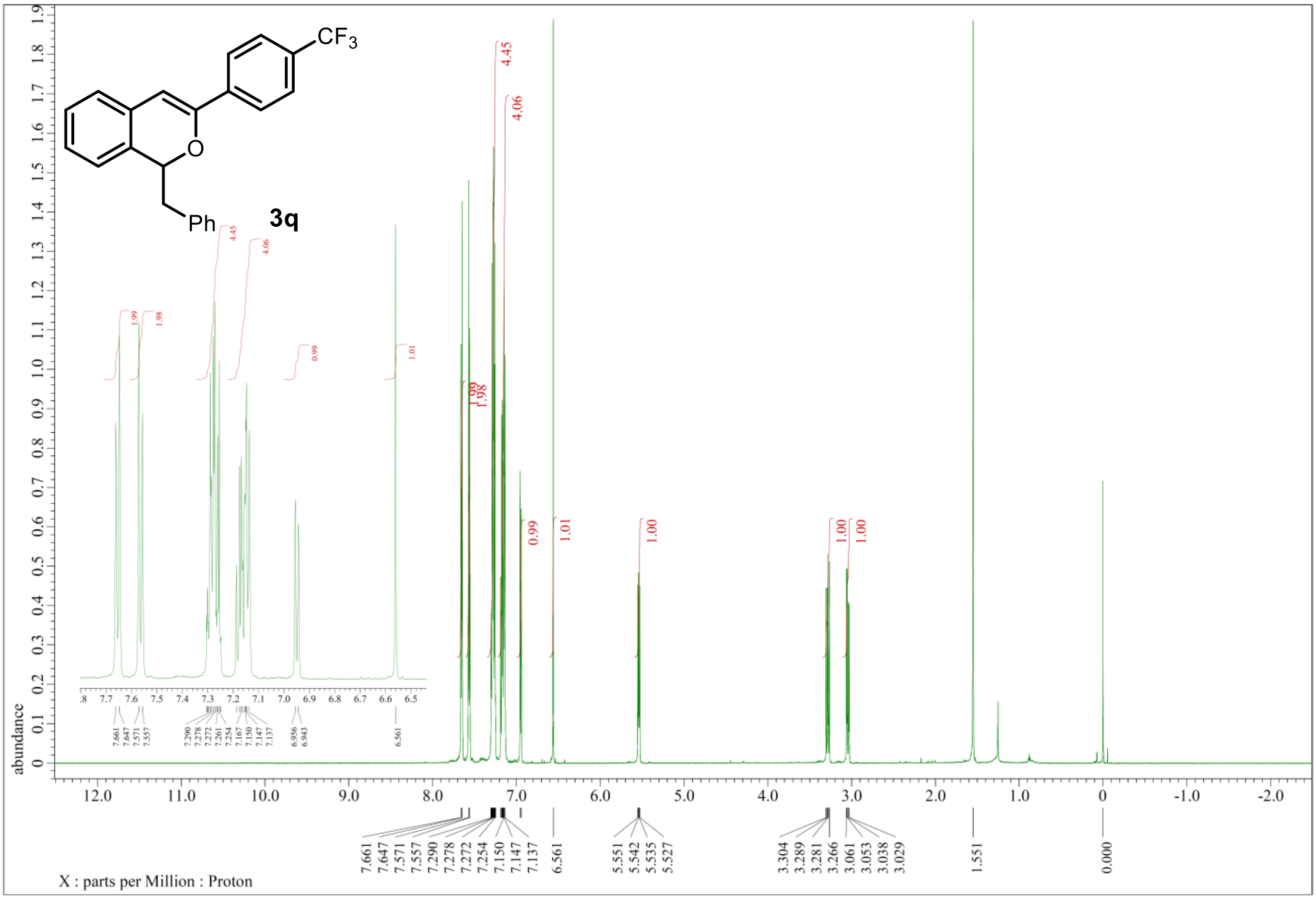

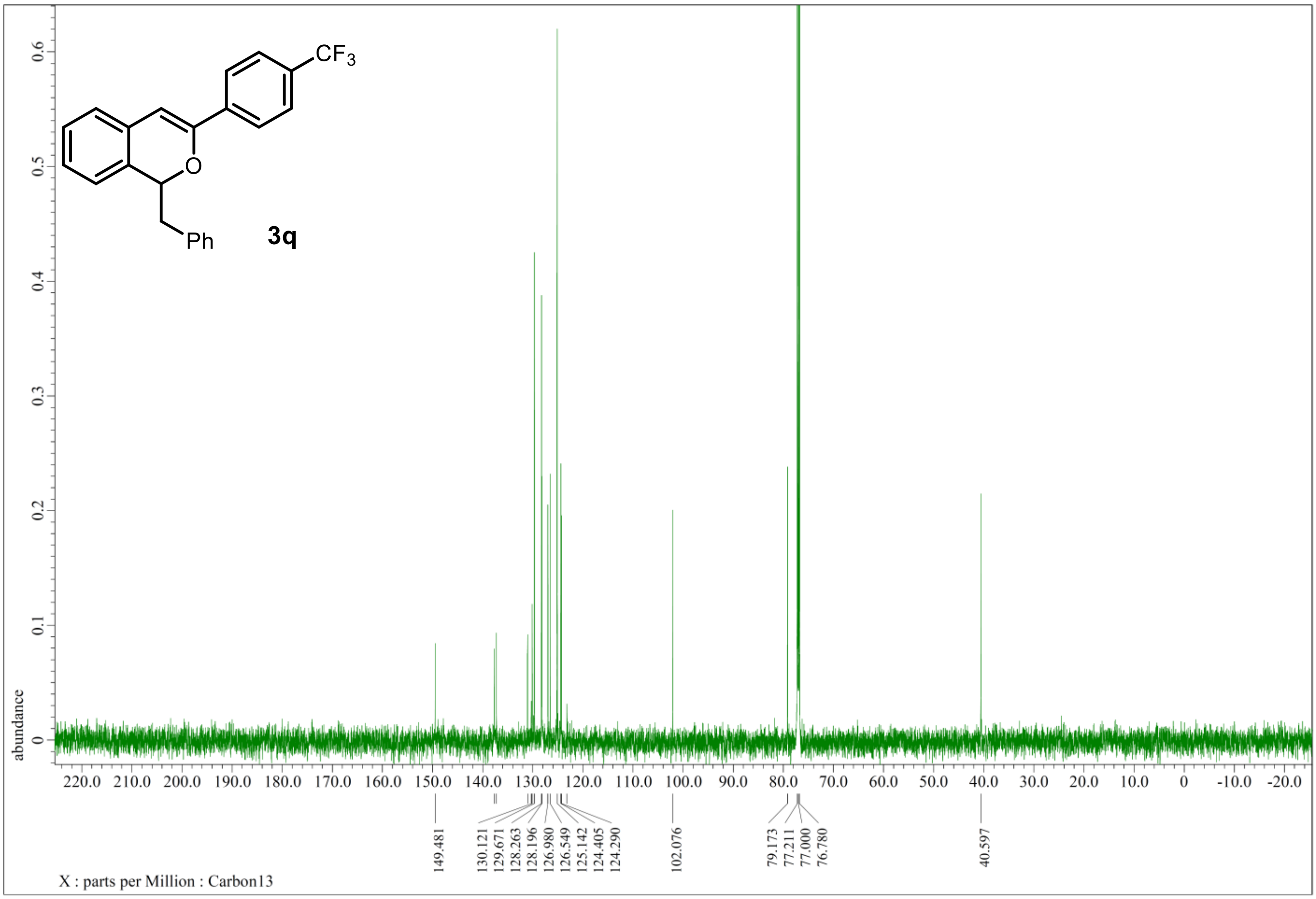

^{1}H NMR (600 MHz, $CDCl_3$) and $^{13}C\{^1H\}$ NMR (151 MHz, $CDCl_3$) spectra of **3r**.

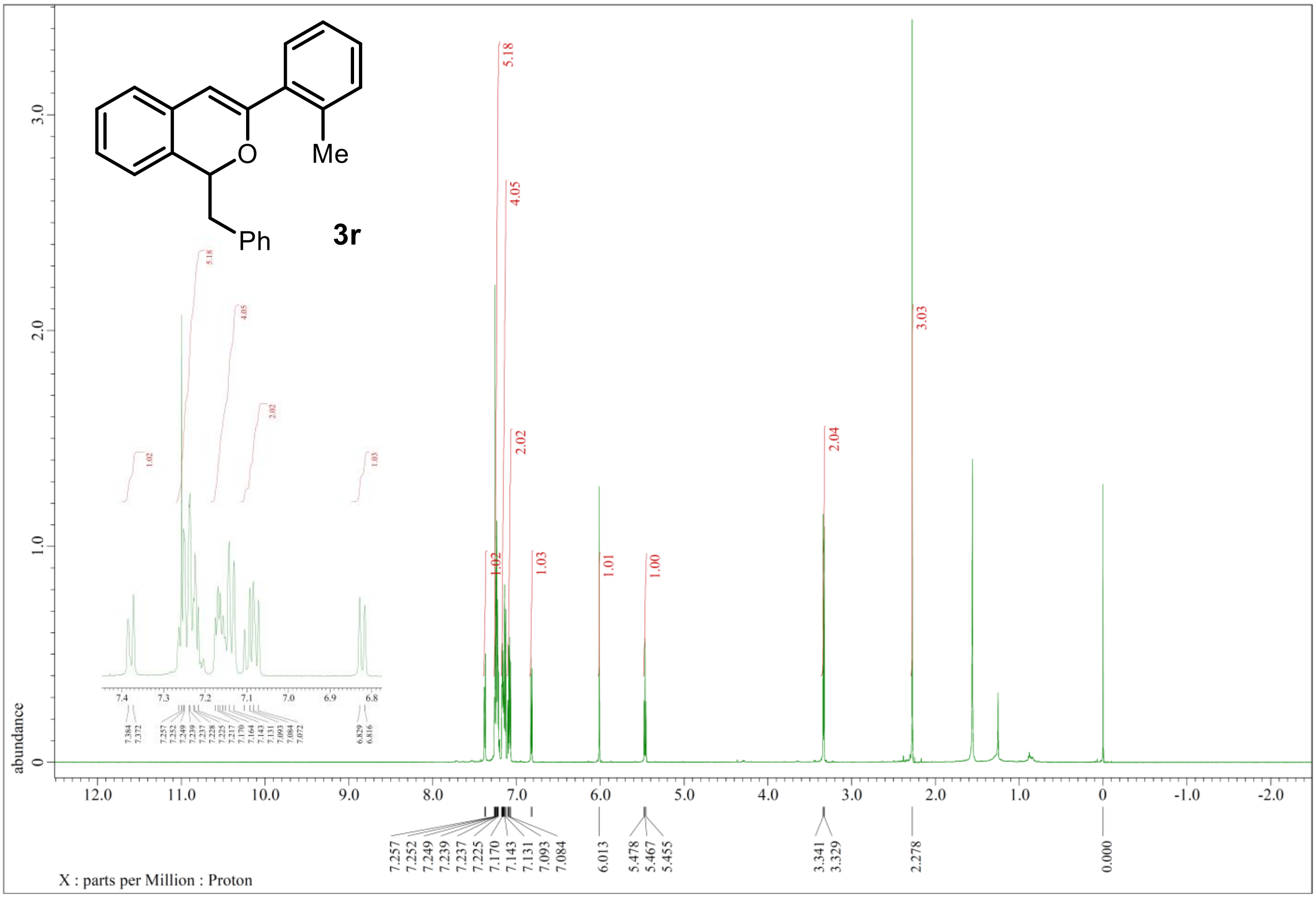

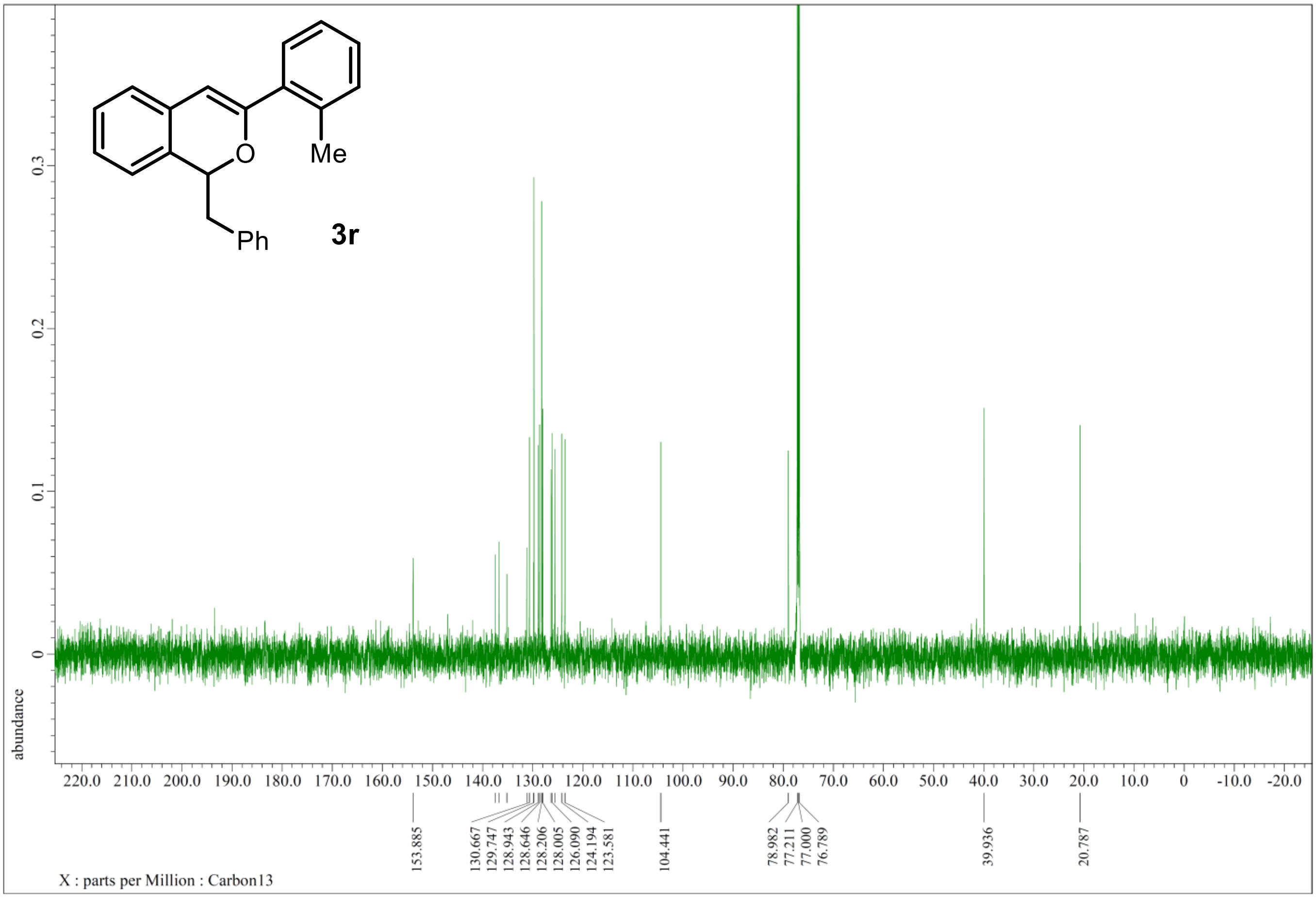

^{1}H NMR (600 MHz, $CDCl_3$) and ^{13}C{^{1}H} NMR (151 MHz, $CDCl_3$) spectra of **3s**.

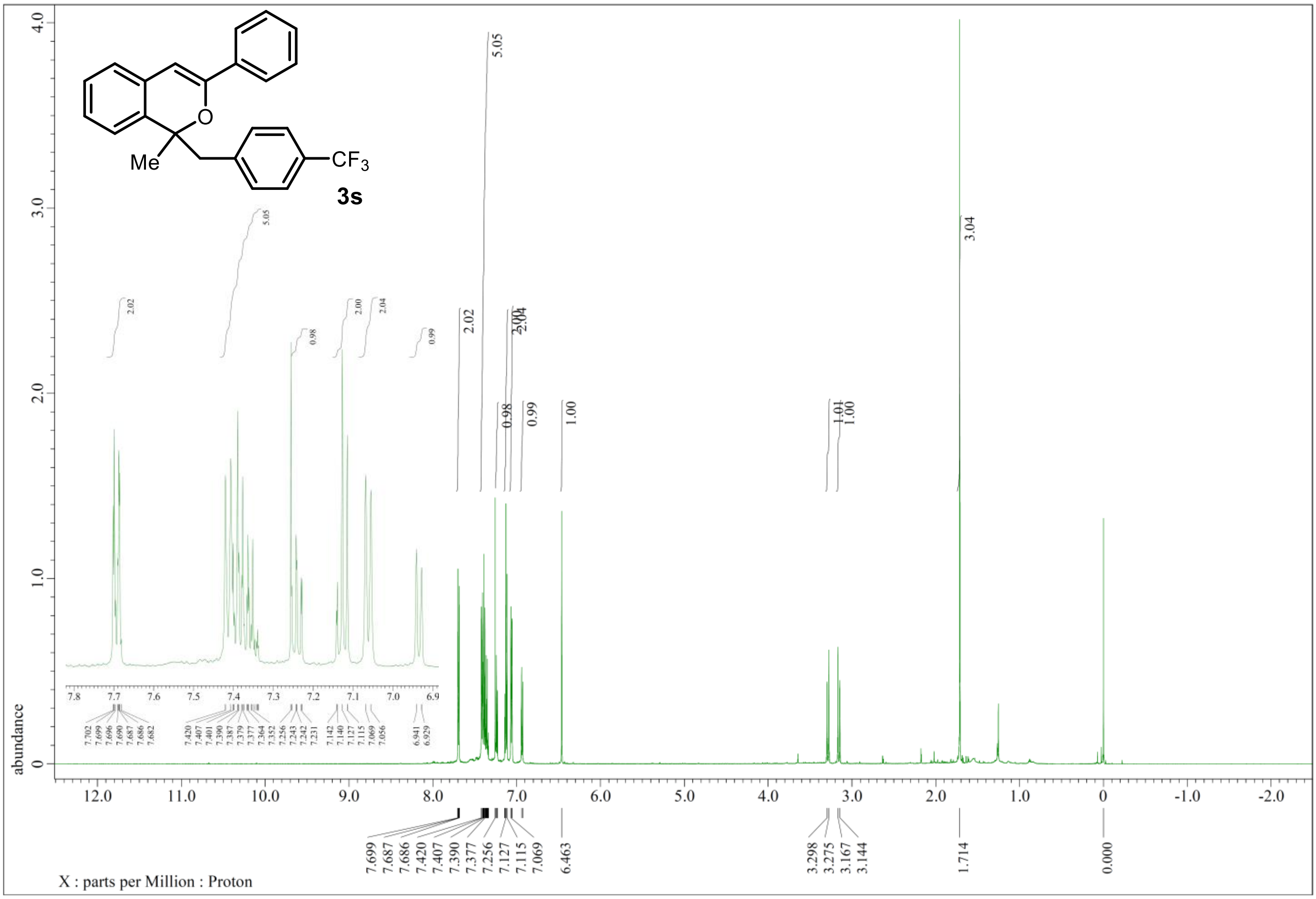

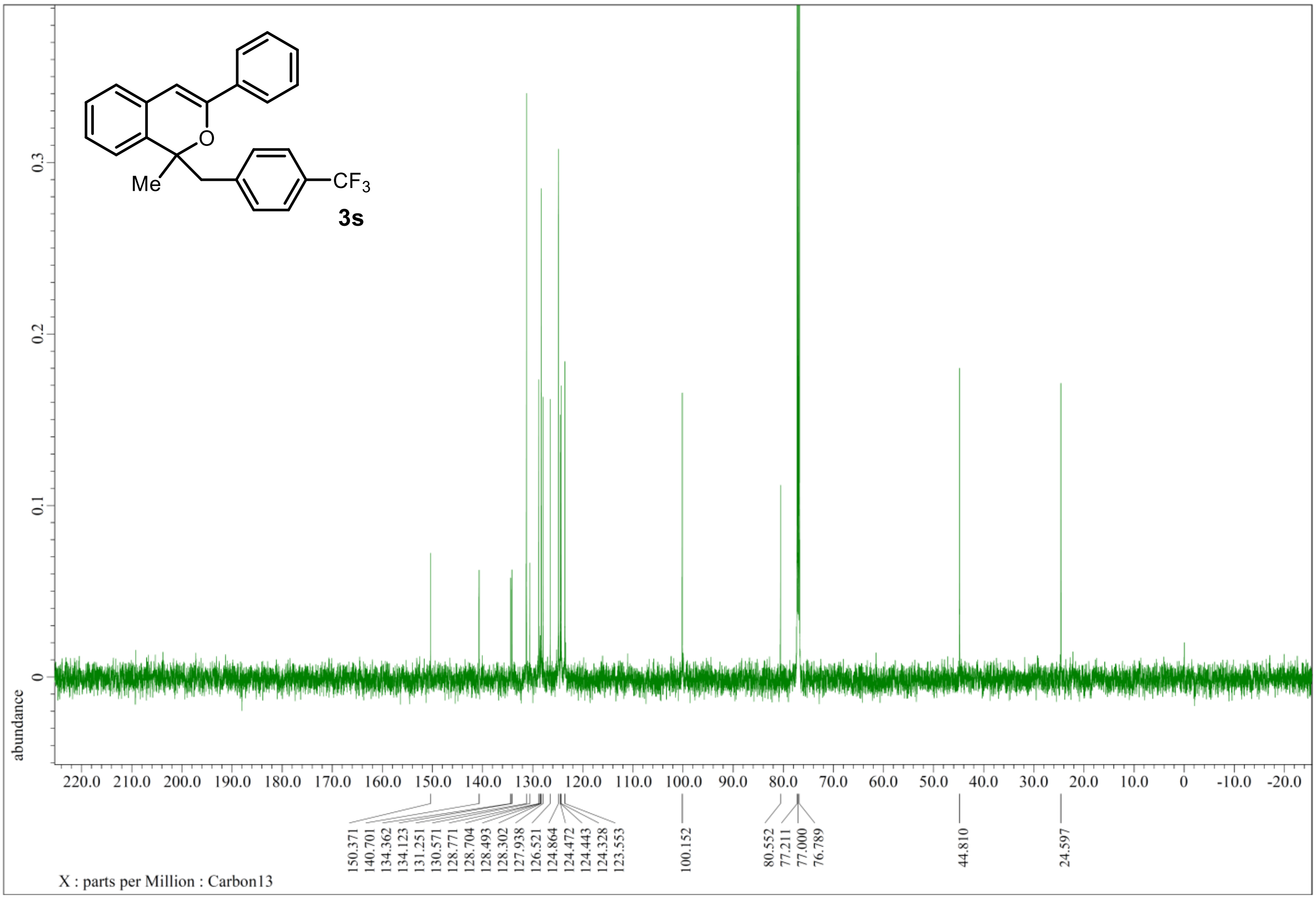

^1H NMR (600 MHz, $CDCl_3$) and $^{13}C\{^1H\}$ NMR (151 MHz, $CDCl_3$) spectra of **3t**.

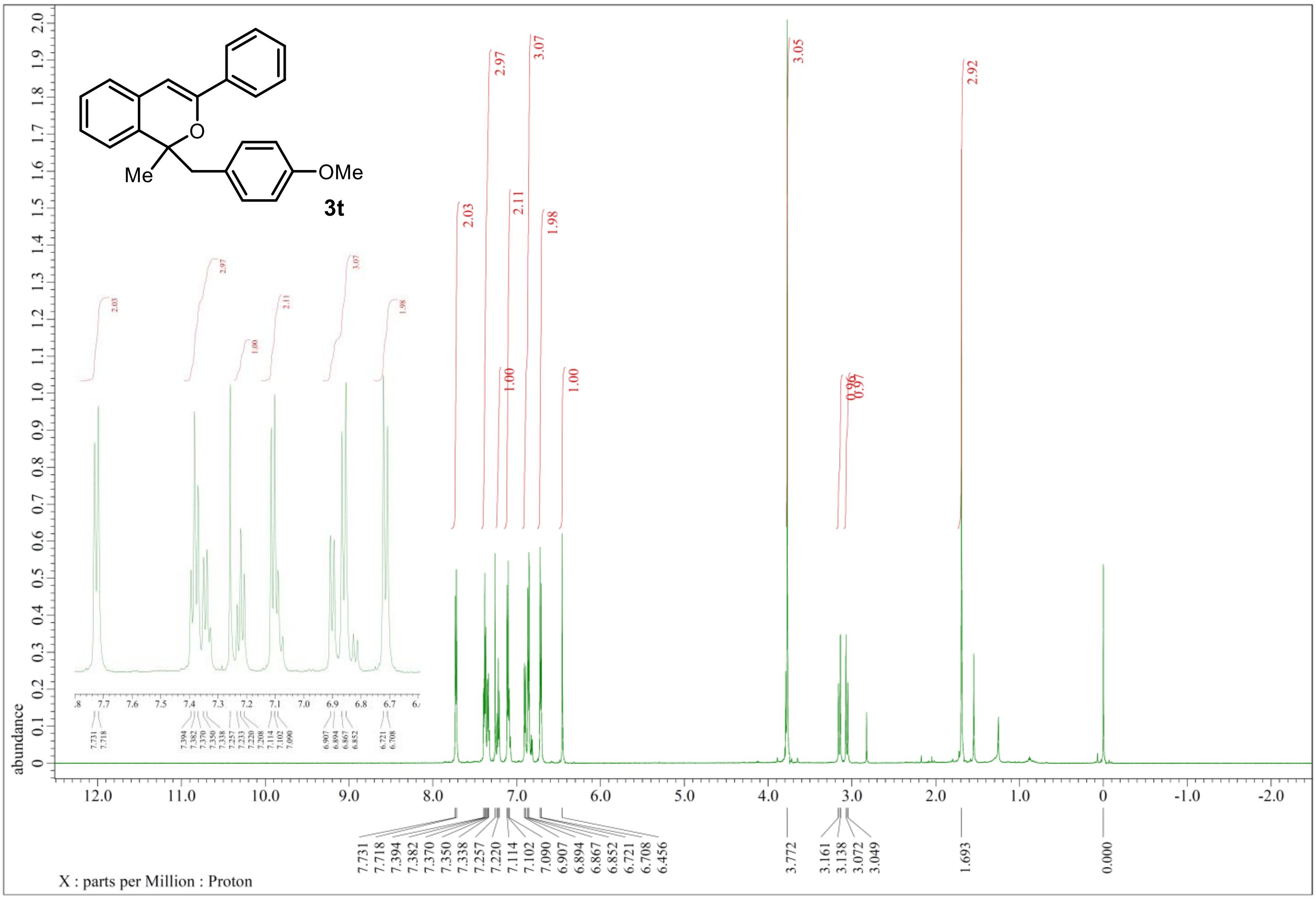

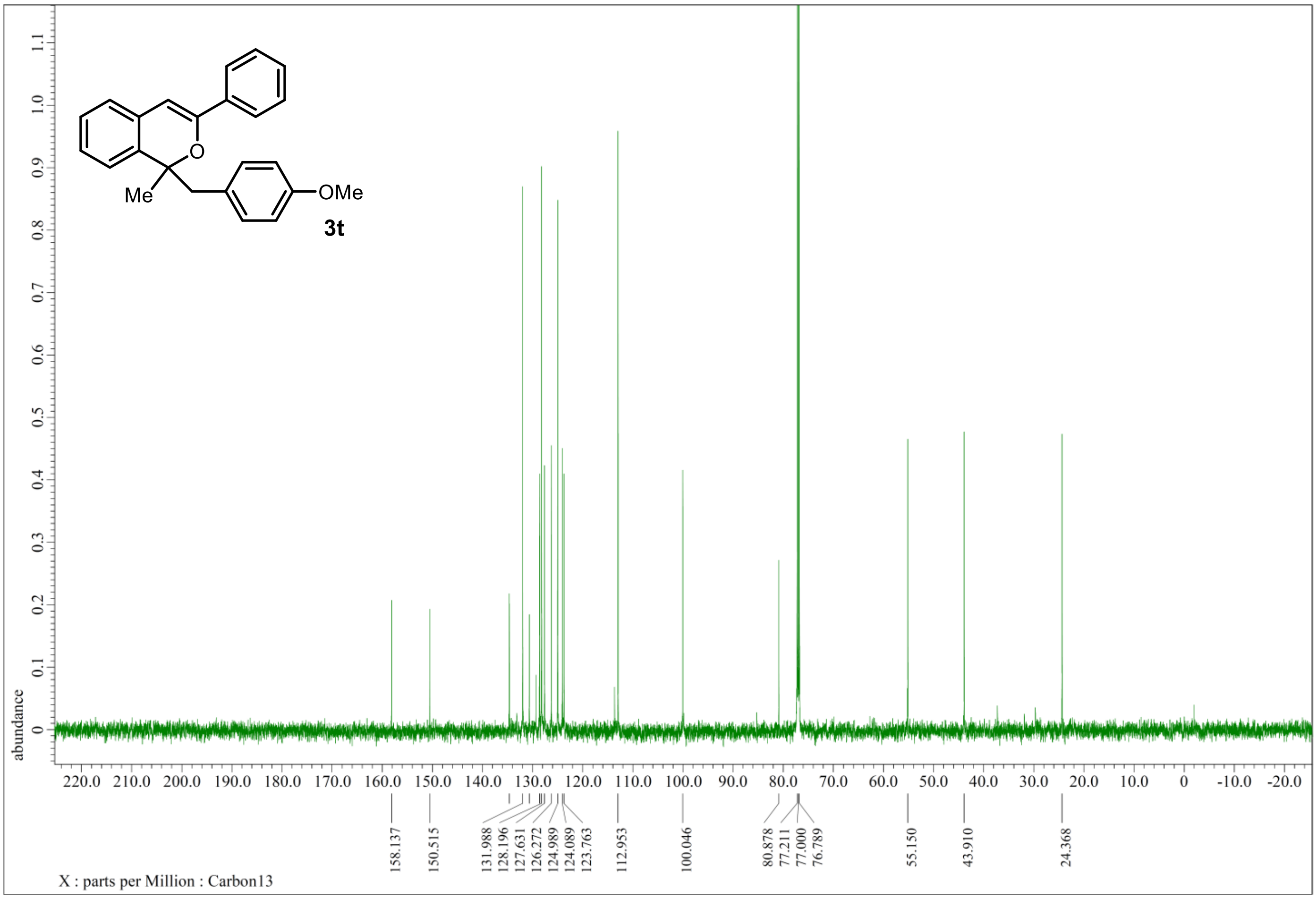

Supplement: File 2 — Copies of NMR spectra. [file Beilstein_J_Org_Chem-20-1973-s002.pdf]
